# High-throughput screen with the l,d-transpeptidase Ldt_Mt2_ of *Mycobacterium tuberculosis* reveals novel classes of covalently reacting inhibitors†

**DOI:** 10.1039/d2sc06858c

**Published:** 2023-05-30

**Authors:** Mariska de Munnik, Pauline A. Lang, Francisco De Dios Anton, Mónica Cacho, Robert H. Bates, Jürgen Brem, Beatriz Rodríguez Miquel, Christopher J. Schofield

**Affiliations:** a Chemistry Research Laboratory, Department of Chemistry, the Ineos Oxford Institute of Antimicrobial Research, University of Oxford 12 Mansfield Road Oxford OX1 3TA UK christopher.schofield@chem.ox.ac.uk; b Tres Cantos Medicines Development Campus, GlaxoSmithKline Calle Severo Ochoa 2, Tres Cantos Madrid Spain beatriz.rodriguez@gsk.com

## Abstract

Disruption of bacterial cell wall biosynthesis in *Mycobacterium tuberculosis* is a promising target for treating tuberculosis. The l,d-transpeptidase Ldt_Mt2_, which is responsible for the formation of 3 → 3 cross-links in the cell wall peptidoglycan, has been identified as essential for *M. tuberculosis* virulence. We optimised a high-throughput assay for Ldt_Mt2_, and screened a targeted library of ∼10 000 electrophilic compounds. Potent inhibitor classes were identified, including established (*e.g.*, β-lactams) and unexplored covalently reacting electrophilic groups (*e.g.*, cyanamides). Protein-observed mass spectrometric studies reveal most classes to react covalently and irreversibly with the Ldt_Mt2_ catalytic cysteine (Cys354). Crystallographic analyses of seven representative inhibitors reveal induced fit involving a loop enclosing the Ldt_Mt2_ active site. Several of the identified compounds have a bactericidal effect on *M. tuberculosis* within macrophages, one with an MIC_50_ value of ∼1 μM. The results provide leads for the development of new covalently reaction inhibitors of Ldt_Mt2_ and other nucleophilic cysteine enzymes.

## Abstract 2

10 000 electrophilic compounds were screened for inhibition of the essential l,d-transpeptidase (Ldt_Mt2_) of *Mycobacterium tuberculosis*. Potent covalently reacting inhibitors were identified and characterised by protein X-ray and MS studies.

## Graphical abstract

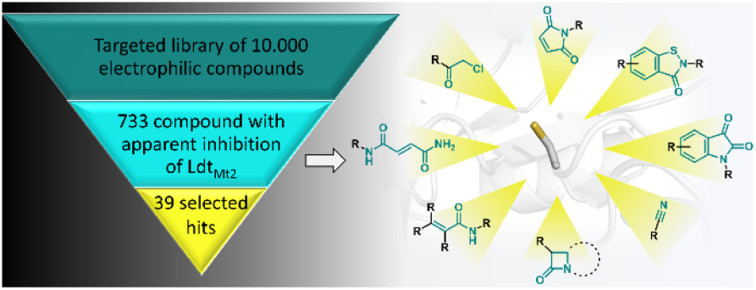

## Introduction

Tuberculosis (TB), caused by *Mycobacterium tuberculosis*, is estimated to account for 1.4 million deaths per year, imposing a major impact on global health.^1^ Current TB treatments typically comprise a combination of three antibiotics taken for 3–6 months, and are effective in ∼85% of cases.^2^ However, in resistant strains, which are endemic in eastern Europe and central Asia, therapeutic effectiveness is reduced to <60%.^3^ There is thus a clear need for improved treatments targeting *M. tuberculosis*.

β-Lactams are the most widely used antibacterials, though historically they have not been deemed to be generally useful for TB treatment, in part due to resistance mediated by the chromosomally encoded *M. tuberculosis* serine β-lactamase BlaC.^4,5^ β-Lactams are covalently reacting inhibitors of the transpeptidases (penicillin binding proteins, PBPs) that catalyse the essential *meso*-Dap-d-Ala (4 → 3) cross-linking transpeptidation step in peptidoglycan biosynthesis.^6^ A mechanistically related class of enzymes, the l,d-transpeptidases (Ldts), also catalyses the formation of cross-links, but rather than 4 → 3 cross-links the Ldts produce *meso*-Dap-*meso*-Dap (3 → 3) cross-links.^7^ By contrast with PBPs, which employ a nucleophilic serine-residue in catalysis, the Ldts employ a nucleophilic cysteine-residue.^7,8^ While only low levels of 3 → 3 cross-linking are evident in the exponential growth phase of *M. tuberculosis*, they comprise ∼80% of all cross-links in the stationary phase.^9^ Ldt_Mt2_ in particular has been identified as being essential for *M. tuberculosis* virulence, making it an attractive target for TB treatment.^10^

**Fig. 1:**
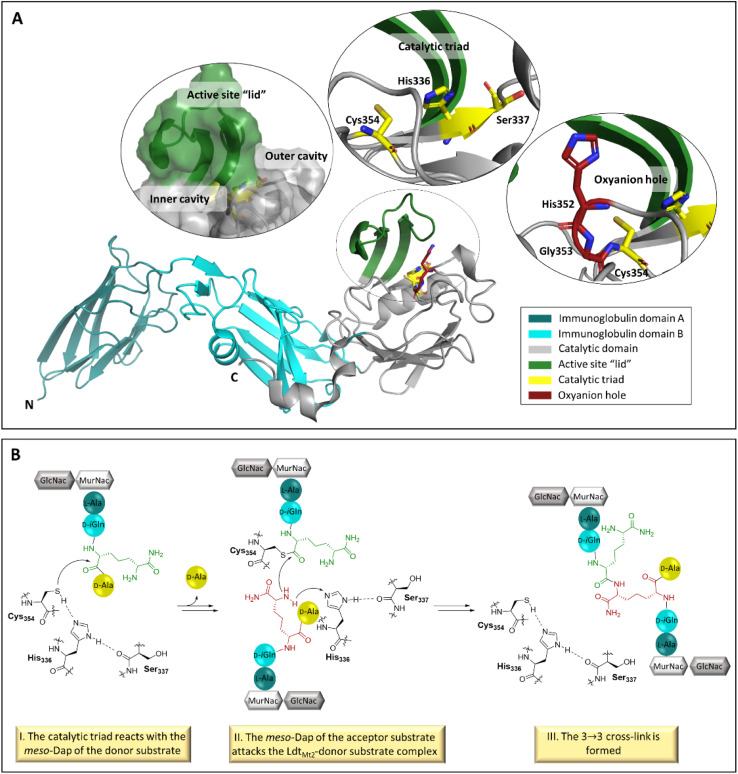
The structure and function of Ldt_Mt2_. (A) Views from a crystal structure of Ldt_Mt2_ (PDB 6RLG),^19^ showing entrances to the active site, the catalytic triad (consisting of residues Cys354, His336 and Ser337) and the oxyanion hole (consisting of the backbone NHs of His352, Gly353 and Cys354). The two Ldt_Mt2_ immunoglobulin-like domains are in teal and cyan, the catalytic domain is in grey, the active site “lid” is in green, the catalytic triad is in yellow, and the oxyanion hole is in red. (B) Outline mechanism for reaction of Ldt_Mt2_ and the disaccharide tetrapeptide monomer of the *M. tuberculosis* peptidoglycan. The Ldt_Mt2_ catalytic triad residues are in black, the *meso*-Dap residue of the donor substrate in green, and the *meso*-Dap residue of the acceptor substrate in red.

The extramembrane section of Ldt_Mt2_ consists of three domains, two of which have an immunoglobulin-related fold, and the catalytic domain which has an ErfK/YbiS/YhnG fold (Fig. 1A).^11^ The Ldt_Mt2_ active site within the catalytic domain is bordered by a flexible loop (residues 300–323), sometimes referred to as the active site “lid”, which creates two entrances to the active site: the inner and outer cavities.^11^ The Ldt mechanism is proposed to be analogous to that proposed for the PBPs, although employing a catalytic triad analogous to that observed in some cysteine-proteases, involving Cys354, His336 and Ser337, for the formation of the cross-link between the donor and acceptor stem substrates (Fig. 1A and B).^12^ The formation of a negatively charged tetrahedral enzyme-substrate intermediate is proposed to be stabilised by an oxyanion hole, consisting of the backbone NH groups of His352, Gly353 and Cys354 (Fig. 1A).^13^

In general, the Ldts are not reported to be efficiently inhibited by β-lactams, however, their nucleophilic cysteine reacts with the β-lactam rings of penems and carbapenems to form acyl–enzyme complexes.^14–16^ Efforts have been made to identify β-lactams which more efficiently inhibit Ldt_Mt2_, though to date with limited success.^17,18^ In an alternative strategy for Ldt_Mt2_ inhibition, we have reported on the inhibition of Ldt_Mt2_ by cysteine-reactive reagents such as ebselen.^19^ The covalent modification of nucleophilic cysteine residues is a validated method for clinically useful inhibition of various cysteine proteases, including the main protease (M^pro^) of SARS-CoV-2, highlighting a need for new functional groups targeting nucleophilic cysteine residues.^20–22^

Here we report on the development of a fluorescence based high-throughput screen (HTS) for Ldt_Mt2_ inhibition and its application to screen a library of potential nucleophilic cysteine reacting compounds. The results reveal the discovery of new mechanism-based nucleophilic cysteine enzyme targeting inhibitors.

## Results

### Optimisation and validation of the high-throughput screen for inhibition of Ldt_Mt2_ and BlaC

We optimised our fluorogenic assay for Ldt_Mt2_ that employs the cysteine-reactive probe 1 (ref. 23) (Fig. S1†) for application in HTS. Cross titrations identified the optimal protein and probe concentrations to be 300 nM and 15 μM, respectively (Fig. S2A and B),† giving a *Z*′ value^24^ of 0.87. Reaction volumes could be reduced to 10 μL without significant reduction in the *Z*′ value (Fig. S2C†). Addition of the detergent Tween-20 to the previously identified preferred buffer conditions (50 mM sodium phosphate, pH 7.5)^23^ improved assay robustness (Table S1†). The combined optimisation efforts (Fig. S2†) led to an assay with a *Z*′ of 0.87, well above the accepted cut-off value of 0.4.^25^

The assay manifested good tolerance for DMSO concentrations of ≤3% (v/v) (Fig. S2F†). Stock solutions of Ldt_Mt2_ and probe 1 in assay buffer were apparently stable over 7 hours when the reagents were kept on ice (Table S2†). No evidence for nonspecific plate patterns was observed during the first hour after reaction initiation under the optimised conditions (Fig. S3†). The assay was validated in dose–response analyses of independent repeats in quadruplicate using a set of 24 tool compounds with known inhibitory activity for Ldt_Mt2_ (Table S3†); the results showed high reproducibility and good correlation with reported values.^19,23^

A previously optimised and validated β-lactamase assay^26^ was optimised for our assay set-up with BlaC, which we envisioned as a secondary assay for the HTS output. Cross titrations showed the optimal BlaC concentration to be 2.5 nM and the optimal probe concentration to be 2.5 μM in an assay volume of 10 μL (Fig. S4†), leading to a robust *Z*′ of 0.84. A dose–response assay with the set of 24 tool compounds was performed in quadruplicate independent repeats to validate the assay, showing high reproducibility (Table S3†).

### Robustness validations throughout the high-throughput screen

To investigate the robustness and quality of the assay throughout the HTS for Ldt_Mt2_ and BlaC, *Z*′ was calculated for each analysed plate; only plates with *Z*′ ≥ 0.4 were accepted for analysis. Average *Z*′ values of 0.64 and 0.76 were obtained for the HTS with Ldt_Mt2_ and BlaC, respectively (Fig. S5†). Throughout the HTS, high reproducibility for the obtained pIC_50_ values of the tool compounds was obtained (Fig. S6, Table S4).† Further, good correlation between the results of independent, blind repeats was observed (Fig. S7†).

### High-throughput screen of a targeted library has been performed

**Fig. 2:**
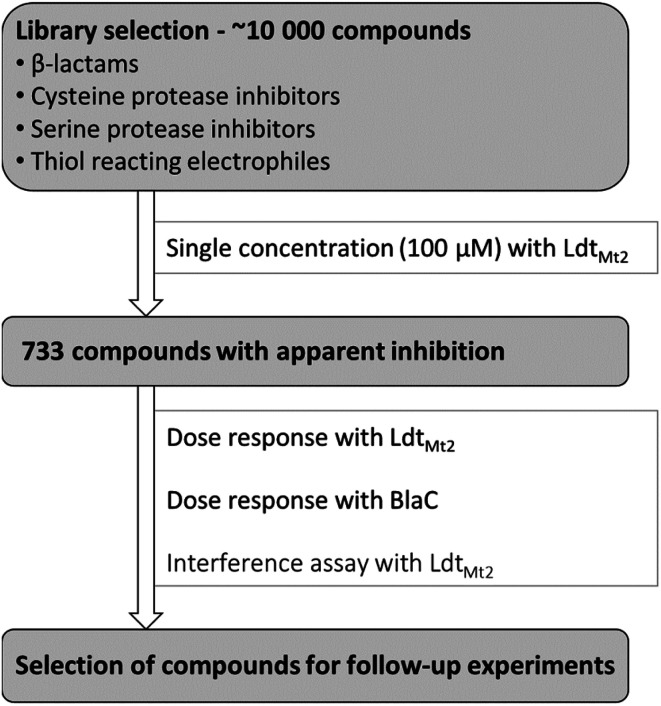
Approach for a high-throughput screen to identify Ldt_Mt2_ inhibitors. A targeted library of approximately 10 000 electrophilic compounds was selected, consisting of compounds with a β-lactam core, cysteine protease inhibitors, serine protease inhibitors and compounds containing known/potential thiol reacting electrophiles. Compounds were initially screened for inhibitory activity against Ldt_Mt2_ at a single concentration (100 μM), after which apparently active compounds (robust percent cut-off of ≥78.0% and ≥79.3% for respective independent repeats) were further analysed in a dose–response assay with Ldt_Mt2_ and in a secondary screen for dose–response with the β-lactamase BlaC. Identified active compounds were subjected to an interference assay for the Ldt_Mt2_ assay. Based on the combined results, compounds were selected for follow-up experiments.

A targeted library of ∼10 000 electrophilic compounds was constructed from the GlaxoSmithKline high-throughput screening (GSK HTS) collection, including compounds with a β-lactam core, cysteine protease inhibitors, serine protease inhibitors, and compounds containing known nucleophilic cysteine warheads (Fig. 2). These compounds were assessed for inhibition of Ldt_Mt2_ with two independent repeats at a single concentration of 100 μM. The results identified 733 potentially active compounds manifesting >78% apparent inhibition (an overall hit rate of 7.7%). The identified compounds were then subjected to dose–response analyses with Ldt_Mt2_ and BlaC (two independent repeats at concentrations 1.7 nM to 100 μM). In an effort to reduce false positives, an interference assay was performed, wherein the inhibitors were added after the completion of the reaction between Ldt_Mt2_ and probe 1 (5 hours at room temperature). Compounds with a pIC_50_ value >4.0 in the interference assay were excluded from further analyses. Compounds were then prioritised and selected for further studies based on their observed inhibitory potency for Ldt_Mt2_ and structural diversity, employing protein observed MS. The results for 39 selected compounds (1–39) are summarised in Table S5.†

### Hit compounds can be grouped into eight compound classes

**Fig. 3:**
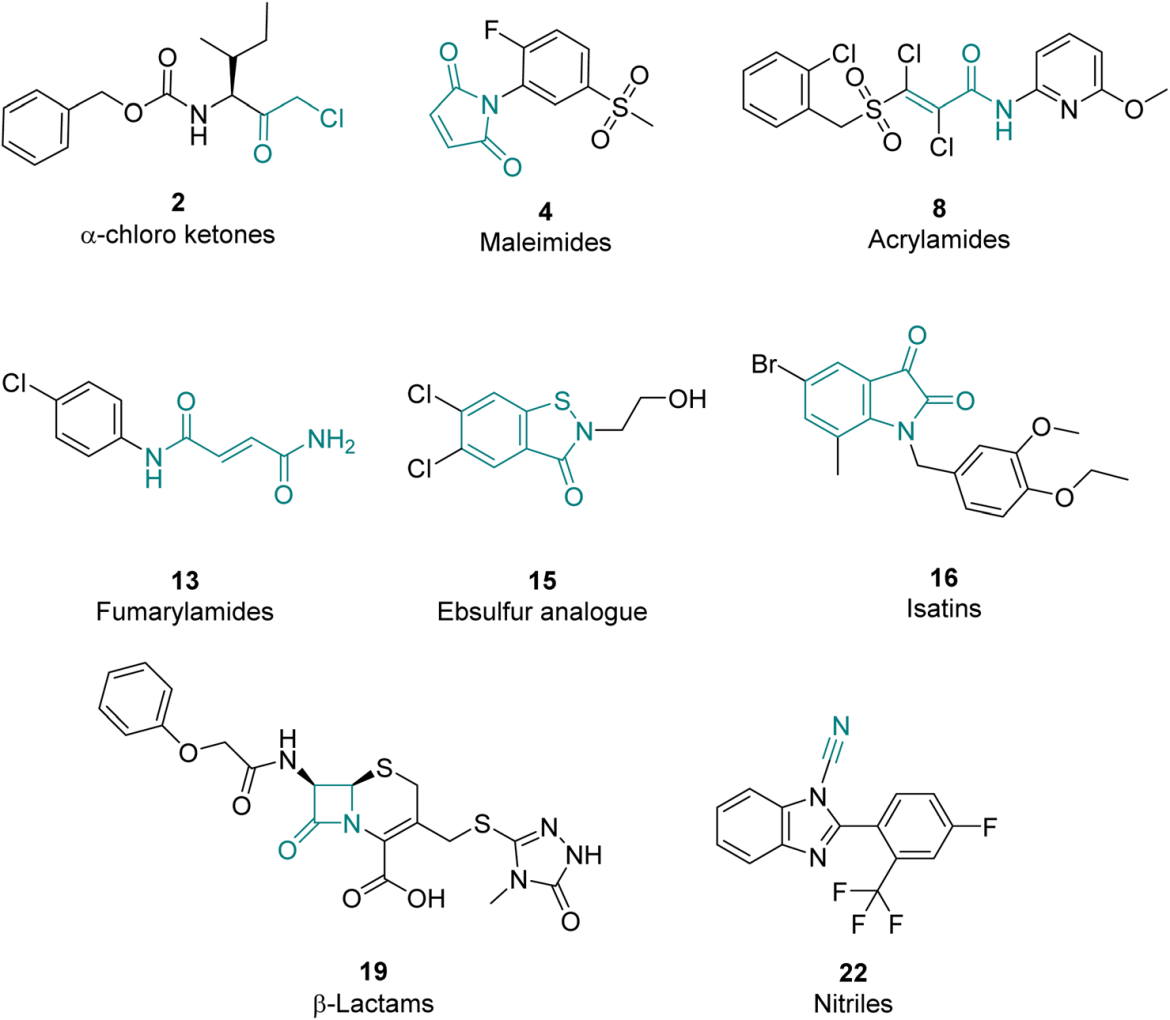
Representative examples of hits for the eight identified Ldt_Mt2_ inhibitor classes. The core electrophilic motif is shown in teal.

**Fig. 4:**
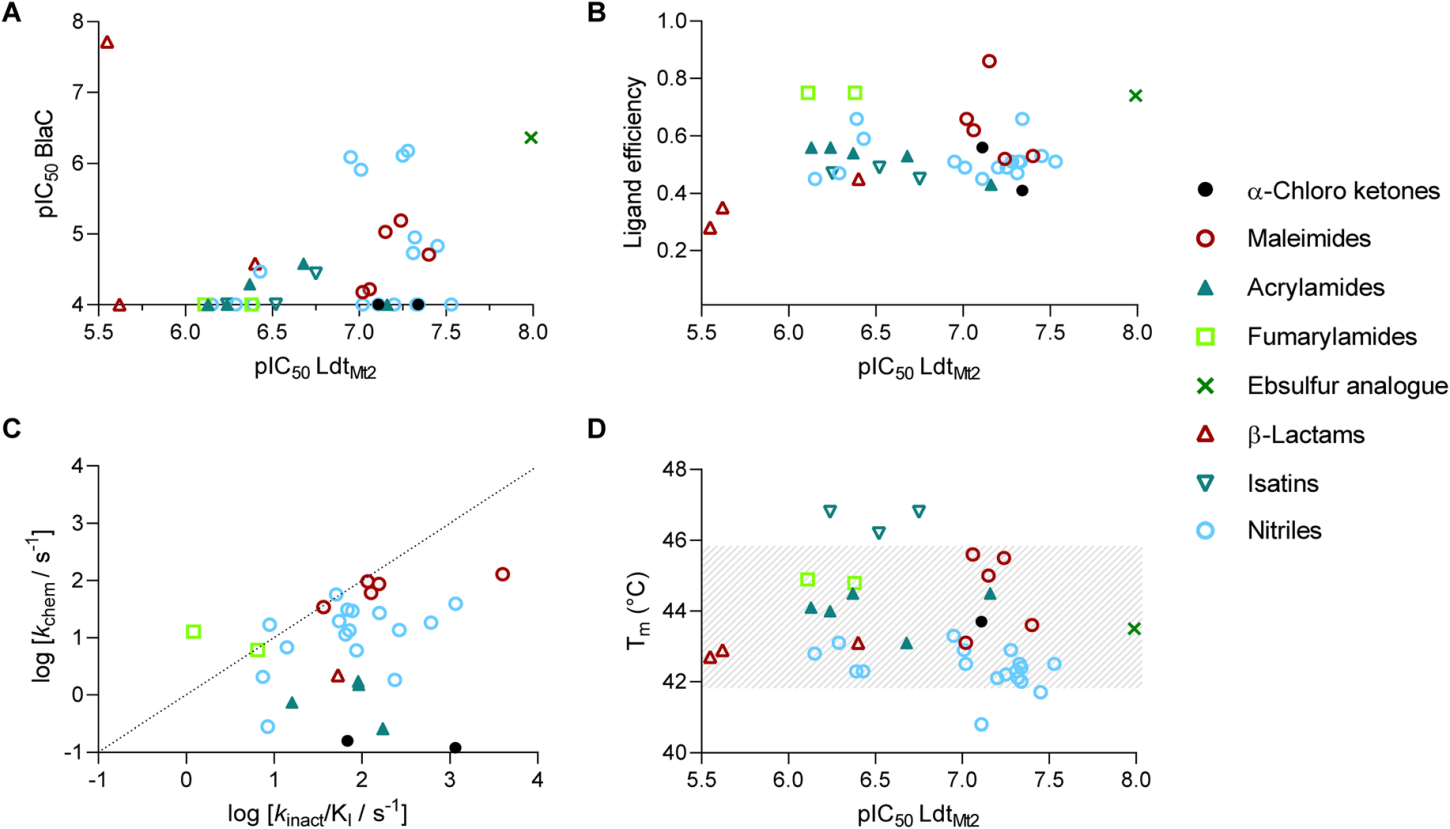
Evaluation of the Ldt_Mt2_ HTS inhibitor hits, clustered by compound class. (A) Potencies of inhibitors identified from the HTS for Ldt_Mt2_ and for BlaC. Most compounds show higher potency for Ldt_Mt2_ compared to BlaC. (B) Ligand efficiency of HTS hits plotted *versus* potency against Ldt_Mt2_. (C) *k*_chem_ of the identified compounds plotted *versus* the *k*_inact_/*K*_I_ for Ldt_Mt2_. Most compounds show higher reactivity with Ldt_Mt2_ compared to the intrinsic thiol reactivity. (D) Melting points (*T*_m_s) of Ldt_Mt2_ in the presence of HTS hits plotted *versus* potency against Ldt_Mt2_. Grey dashes represent the area of non-significant change with respect to the unmodified protein (43.8 ± 2 °C).

The 39 selected hit compounds (Table S5†) cluster into eight distinct groups based on their proposed cysteine reactive functional groups: α-chloro ketones, maleimides, acrylamides, fumaryl amides, an ebsulfur analogue, isatins, nitriles, and β-lactams (Fig. 3). Overall, pIC_50_ values of 5.55–7.99 were observed for the inhibition of Ldt_Mt2_ by the selected compounds (Fig. 4A, Table S5, Fig. S8†), with ebsulfur analogue 15 being most potent inhibitor (pIC_50_ 7.99). The majority of the hit compounds have molecular masses of 200 to 350 Da, and have a good ligand efficiency (0.28–0.86, Fig. 4B), leaving scope for further development.

Notably, two of the identified Ldt_Mt2_ inhibitors belong to the cephalosporin subclass of β-lactams (19, 20); this observation is interesting because cephalosporins have previously been deemed not to be of use for inhibition of Ldt_Mt2_, potentially because their stereochemistry mimicks the d-Ala-d-Ala substrate of PBPs rather than that of Ldts, which accept a substrate with C-terminal l,d-chirality.^23,27,28^ However, the Ldt_Mt2_ inhibition observed with cephalosporins 19–20 (pIC_50_ 5.55–5.62) is comparable to that observed for the carbapenems biapenem, doripenem and meropenem under the same assay conditions (pIC_50_ 5.54–5.87, Table S4†). The results thus imply that the cephalosporin scaffold is of considerable interest for inhibition of Ldt_Mt2_ and hence TB treatment. While most compounds showed no significant inhibition of BlaC, cephalosporin 20 was a potent inhibitor (pIC_50_ 7.72, Fig. S9†).

### Protein observed SPE-MS reveals covalent modification of Ldt_Mt2_

To investigate the mechanism of Ldt_Mt2_ inhibition by selected inhibitors, protein observed solid phase extraction mass spectrometry (SPE-MS)^29^ time course assays were carried out on the reactions of Ldt_Mt2_ with inhibitors 1–21 at varied protein-inhibitor ratios (Fig. S10, Table S6†). As the SPE-MS procedure involved Ldt_Mt2_ denaturation under acidic conditions (0.1% (v/v) aqueous formic acid), observation of adduct formation likely reflects covalent reaction. The results therefore imply that most of the tested compounds (1–11, 13–15 and 19–21) covalently modify Ldt_Mt2_, in most cases leading to an adductcorresponding to apparent irreversible reaction with a single inhibitor molecule (Fig. S10†). An exception was maleimide 6, which apparently modified Ldt_Mt2_ with the reaction involving multiple inhibitor molecules (Fig. S10† entry 6).

The SPE-MS results suggest that α-chloro ketones 1–2 react with Ldt_Mt2_ with loss of a chloride ion, consistent with a nucleophilic substitution involving Cys354 (Fig. S10† entries 1–2). The maleimide derivatives 3–7 reacted with Ldt_Mt2_ without fragmentation, resulting in adducts comprising the intact inhibitor mass (Fig. S10† entries 3–7). Over the course of 24 hours, partial hydrolysis of the ester sidechain of 7 was observed, as manifested by a mass decrease of 106 Da (relative to the modified Ldt_Mt2_, Fig. S10† entry 7).

Acrylamide 8 (MW 436 Da), a *trans*-dichloro substituted Michael acceptor, reacted to give covalently modified Ldt_Mt2_ (+399 Da), corresponding to the addition of a single molecule accompanied with loss of one chloride ion (Fig. S10† entry 8). 9 (MW 306 Da) covalently modified Ldt_Mt2_ (+322 Da, Fig. S10† entry 9), corresponding to the mass of the inhibitor plus an additional 16 Da. A further +16 Da adduct was observed to accumulate over 21 hours, potentially reflecting slow oxidation of the Cys354 sulphur derived thioester following reaction with the inhibitor. While acrylamides 10–12 are structurally closely related, only 10 and 11 were observed to manifest substantial modification of Ldt_Mt2_ (+186 and +298 for 10 and +286 Da for 11, Fig. S10† entries 10–11). By contrast, reaction with 12 (MW 268 Da) manifested only low amounts of a +268 Da Ldt_Mt2_ modification (Fig. S10† entry 12).

Although the fumaryl amides 13 and 14 (MW 225 and 204 Da, respectively) contain a potential Michael acceptor, the SPE-MS experiments solely showed +207 Da and +189 Da adducts on reaction of Ldt_Mt2_ with 13 and 14, respectively, in agreement with substitution of the terminal amide resulting in loss of NH_3_ (Fig. S10† entries 13–14). Ebsulfur analogue 15 (MW 264 Da) reacted without apparent fragmentation to give a +264 Da adduct (Fig. S10† entry 15). Isatin derivatives 16 and 17 (MW 404 and 390 Da, respectively) showed only low levels of modification of Ldt_Mt2_ manifesting +404 and +390 Da mass increments, respectively, but addition of 2 molecules of 17 was observed (Fig. S10† entries 16, 17). 18 (MW 366 Da) was not observed to modify Ldt_Mt2_ (Fig. S10† entry 18). β-Lactams 19–21 exhibited complex fragmentation patterns, which were observed to vary with time and inhibitor concentration (Fig. S10† entries 19–21). However, a +626 Da adduct, corresponding to the addition of a non-fragmented molecule of 20 (MW 626 Da), dominated at lower inhibitor to enzyme ratios.

### Most hits react selectively with Cys354

Ldt_Mt2_ contains only a single cysteine residue (Cys354). However, as several identified electrophiles are able to react with residues other than cysteine,^30,31^ it was assessed whether covalent modification of Ldt_Mt2_ by the inhibitors is related to reaction with Cys354. Thus, Ldt_Mt2_ was preincubated with the non-reversible covalent inhibitor ebselen, which has been shown to react selectively with Cys354.^19^ Apparently complete Ldt_Mt2_ modification by a single molecule of ebselen (+274 Da) was observed. Inhibitors 1–21 were then added to the ebselen derived Ldt_Mt2_ complex (Fig. S11†). With Cys354 unavailable, only 6, 16 and 17 were observed to react with the Ldt_Mt2_-ebselen complex. By contrast, 1–6, 7–15, and 19–21 no longer reacted with Ldt_Mt2_, supporting selective binding to Cys354 (or close-by residues in the active site).

### Isatins 16–18 are reversibly binding Ldt_Mt2_ inhibitors

As the isatins 16–18 showed a lack of clear evidence for irreversible covalent binding to Ldt_Mt2_ in the SPE-MS studies, their mechanism of inhibition was further assessed. By contrast with the SPE-MS studies, nondenaturing MS analysis of Ldt_Mt2_ in the presence of 16–18 showed substantial binding, resulting in mass increments of +404 Da and +366 Da for 16 and 18, respectively, corresponding to binding of a single inhibitor molecule (Fig. S12A†). On incubation with 17, +390 Da and +780 Da mass adducts were observed, corresponding to binding of 1 or 2 inhibitor molecules, respectively. On increasing the cone voltage from 100 V to 200 V, adducts indicating binding of 16–18 were no longer observed, supporting the reversible binding of the isatin inhibitors.

In thermal shift assays^32^ stabilisation by isatins 16–18 was observed, increasing the melting temperature of Ldt_Mt2_ by 2.4–3 °C (Fig. 4D and S12B†). By contrast, the other hits did not show a substantial change in the melting temperatures of Ldt_Mt2_, apart from nitriles 26 and 36, which lowered the melting temperature by 2.1 and 3.0 °C, respectively (Table S5†). The reversibility of isatin binding was assessed by jump dilution studies.^33^ While inhibition of the inhibitors 1–11, 13–15 and 19–39 was found to be irreversible, inhibition by isatins 16–18 was reversible with *k*_off_ values of 103 s^−1^, 84.6 s^−1^, and 160 s^−1^, respectively, resulting in half-lives of the Ldt_Mt2_ inhibitor complexes of 24.6 s, 29.4 s, and 15.6 s, respectively (Fig. S12C, Table S7†). Dose–response assays at varying inhibitor pre-incubation times (ranging between 0 min and 2 h) manifested no time-dependency for potency of inhibition by the isatins 16–18 (Fig. S12D, Table S8†). The reversible binding mode of the isatins, either *via* reversible noncovalent binding, or reversible covalent binding involving reaction of the nucleophilic Cys354 with the C3 isatin ketone, differ from most of the classes identified, which involve irreversible inhibition *via* alkylation, substitution, or acylation mechanisms.

### Nitriles 22–36 are electrophilic cyanating agents

**Fig. 5:**
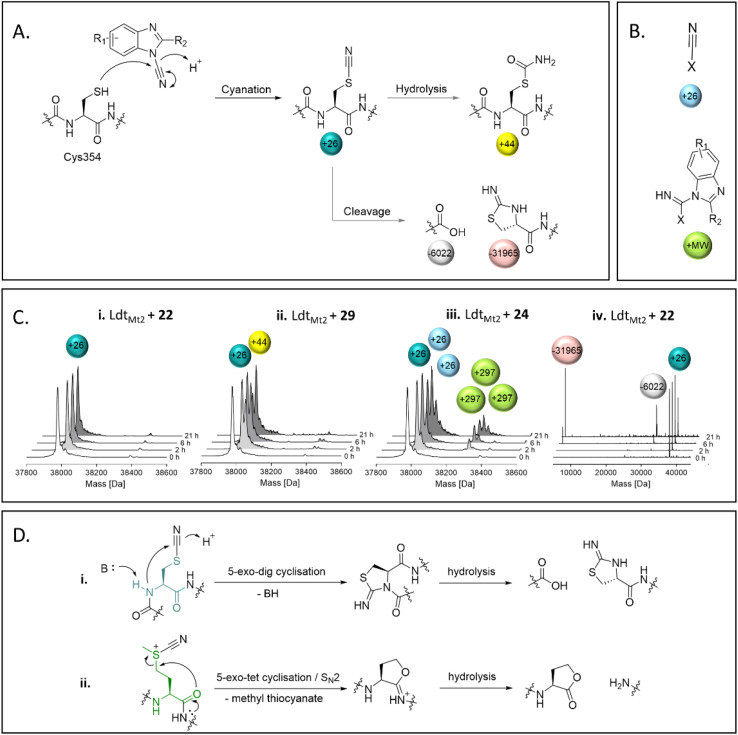
Reaction of Ldt_Mt2_ with cyanamides 22–36 as observed by mass spectrometry. (A) Cys354 of Ldt_Mt2_ is cyanated by cyanamides 22–36, which can be followed by hydrolysis, or cleavage adjacent to Cys354. (B) Apparent adducts occasionally observed that cannot be related to reaction with Cys354. The non-Cys354 reacting residue is represented by X. (C) Representative deconvoluted mass spectra from the reaction between the cyanamides and Ldt_Mt2_, showing evidence for (i) cyanation, (ii) cyanation followed by hydrolysis, (iii) reaction with multiple molecules of the inhibitor, and (iv) cleavage of Ldt_Mt2_ at Cys354 following cyanation. (D) Mechanisms for the cleavage of proteins through cyanation of (i) cysteine (N-terminal side; the cysteine residue is in teal) or (ii) methionine (C-terminal side; the methionine residue is in green) residues.

SPE-MS assays of the reaction between nitriles 22–39 and Ldt_Mt2_ identified cyanamides 22–36 as electrophilic cyanating agents (Fig. 5, Fig. S13 entries 1–15, Table S9†), as apparent by a mass shift of +26 Da, reflecting a transfer of a nitrile group. In certain cases, this was accompanied by an additional modification of +17 Da, which likely reflects the addition of water to the transferred nitrile group to give an *S*-carbamoyl cysteine residue. By contrast, cyanamide 37 – which does not share the benzimidazole core of 22–36 – was not observed to cyanate Ldt_Mt2_, and instead reacted initially without fragmentation, followed by hydrolysis of the ester of 37 (Fig. S13† entry 16). Reaction of Ldt_Mt2_ with nitriles 38 and 39, both of which have a benzonitrile core, resulted in complex adduct formation (Fig. S13† entries 17–18).

Most of the identified cyanamide inhibitors apparently reacted once with Ldt_Mt2_, predominantly with Cys354; however, 24, 33, 34, 35 and 37 were observed to react with Ldt_Mt2_ multiple times (Fig. S13 and S14†). The latter observation likely relates to reaction with non-cysteine residues, as evidenced by SPE-MS experiments with the Ldt_Mt2_-ebselen complex (Fig. 5B and S14†). The precise reason for the differences in reactivity of the cyanamides is unknown, but it is notable that 33, 34 and 35 all contain a pyridine ring, and 24 has an analogous pyridazine ring. The extent of cyanation increased with increasing pH (as shown with 22, 27, 31 and 36 at pH 5.5–7.5, Fig. S15†). Cyanation appeared to be stable for ∼6 hours, after which time backbone cleavage of Ldt_Mt2_ at Cys354 was observed, as previously reported for the known cysteine cyanating agents 2-nitro-5-thiocyanobenzoic acid (NTCB) and 1-cyano-4-dimethylaminopyridinium tetrafluoroborate (CDAP) (Fig. 5).^34,35^ However, β-elimination, which is reported as a side reaction to protein cleavage by NTCB and CDAP,^35,36^ was not observed. Additionally, cleavage of methionine residues, as reported for treatment of proteins with cyanogen bromine (Fig. 5D),^37^ was not observed.

The cyanating activity of 22–36 was compared to a selection of known electrophilic cyanation agents, including *N*-based cyanating reagents 40–43 (including CDAP) and *S*-based cyanating reagents NTCB (44) and CDTP (45) (Table S10†).^38,39^ SPE-MS studies on Ldt_Mt2_ reaction with 40–44 showed comparable results to those for cyanamides 22–36, with predominantly single- or double-cyanation being observed, while 45 did not modify Ldt_Mt2_ (Fig. S16†). The amount of cleavage of Ldt_Mt2_ at Cys354 was comparable for 22–36 and 40–44 (Fig. S17†). While most of the cyanamides 22–36 potently inhibited Ldt_Mt2_ (pIC_50_ values >7.0), cyanating agents 40–44 were apparently less potent inhibitors (pIC_50_ values 5.3–6.5), while 45 was inactive (Table S10†). The intrinsic thiol reactivity of 40–42 was generally lower (*k*_chem_ 0.90 M^−1^ s^−1^, 0.90 M^−1^ s^−1^, and 3.69 M^−1^ s^−1^, respectively, Table S10†) than observed with 22–36 (exceptions being 28 and 32). Due to assay interference, the intrinsic thiol reactivities of for 43–45 could not be determined.

### Most hits are more reactive towards Ldt_Mt2_ compared to the intrinsic thiol reactivity

With 1–11, 13–14 and 19–39 identified as irreversibly binding Ldt_Mt2_ inhibitors, the second-order rate constant *k*_inact_/*K*_I_, which describes the efficiency of irreversible reaction,^40^ was determined for them. A modified form of a previously reported high-throughput endpoint assay,^41^ based on competition for reaction with the active site cysteine between an irreversibly reacting covalent probe and test compounds, was used employing probe 2 (Fig. S18†). The *k*_inact_/*K*_I_ values were obtained for all identified irreversible inhibitors (Fig. S19, Table S5†), with the exceptions of 15, 19, 20 and 38, due to assay interference. Similarly to the results of the HTS assay, compound classes exhibiting high reactivity were the cyanamides, in particular 23 (265 M^−1^ s^−1^) and 29–31 (1164 ± 105 M^−1^ s^−1^, 236 ± 9.5 M^−1^ s^−1^ and 609 ± 11 M^−1^ s^−1^, respectively) and the maleimides 3–7 (ranging from 36.5 ± 7.9 M^−1^ s^−1^ to 3991 ± 356 M^−1^ s^−1^).

The intrinsic reactivity (*k*_chem_) with a thiol group was assessed for all the selected compounds 1–39 (Table S5†). Following the same principle as used for the assay determining *k*_inact_/*K*_I_, a modified form of a previously reported high-throughput endpoint assay,^42^ based on competition for reaction with glutathione between an irreversibly reacting covalent probe and test compounds, was used employing probe 2 (Fig. S20A†).^43^ The assay was validated using a small number of thiol-reactive compounds (Fig. S20B†), which show good correlation with reported *k*_chem_ values (Fig. S20C, Table S11†).^42^ The irreversibility of the reaction of glutathione with probe 2 was shown over the duration of the measurements (>24 hours) using a displacement assay with *N*-ethylmaleimide (Fig. S20D and E†), as previously reported.^42^

The majority of the compounds showed low to moderate intrinsic thiol (glutathione) reactivity under the tested conditions (*k*_chem_ < 0.08–13.0 M^−1^ s^−1^). The maleimide derivatives (3–7) exhibited relatively higher *k*_chem_ values (34.2–129.9 M^−1^ s^−1^), as anticipated based on their known reactivity as alkylating agents and application in thiol-coupling reactions.^44,45^ A selection of the nitrile derivatives, *i.e.* those containing a cyanamide functional group (22–36), also displayed relatively high *k*_chem_ values (2.12–61.6 M^−1^ s^−1^). Comparison of the obtained *k*_inact_/*K*_I_ values with the *k*_chem_ values, implies an increased reactivity for Ldt_Mt2_ compared to the intrinsic thiol reactivity for the majority of inhibitors (Fig. 4C), implying that with appropriate derivatisation, selective inhibition of Ldt_Mt2_ should be possible.^46^

### X-ray crystallographic studies

To investigate the molecular interactions between the hits and Ldt_Mt2_, X-ray crystallographic studies were carried out using reported Ldt_Mt2_ crystallisation conditions.^19^ As reported, in most cases, Ldt_Mt2_ crystallised in the *P*12_1_1 space group with two molecules (chain A and B) in the asymmetric unit (ASU), though the complex with compound 8 was an exception (see below). Crystal structures of Ldt_Mt2_ reacted with α-chloro ketone 2 (PDB 8A1L,^47^ 2.30 Å resolution), maleimides 3 (PDB 8A1J, 2.55 Å resolution) and 4 (PDB 8A1M, 2.30 Å resolution), acrylamide 8 (PDB 8A1O, 2.05 Å resolution), fumaryl amide 13 (PDB 8A1N, 2.05 Å resolution), ebsulfur analogue 15 (PDB 8A1K, 1.75 Å resolution), and nitrile 31 (PDB 8AHO, 2.30 Å resolution) were obtained through soaking and were solved by molecular replacement (using PDB 6RRM,^19^ as the search model). In all cases, the inhibitors were observed to be covalently bonded to Cys354, and the observed adducts agreed with the MS studies (Fig. S10 and S13†).

**Fig. 6:**
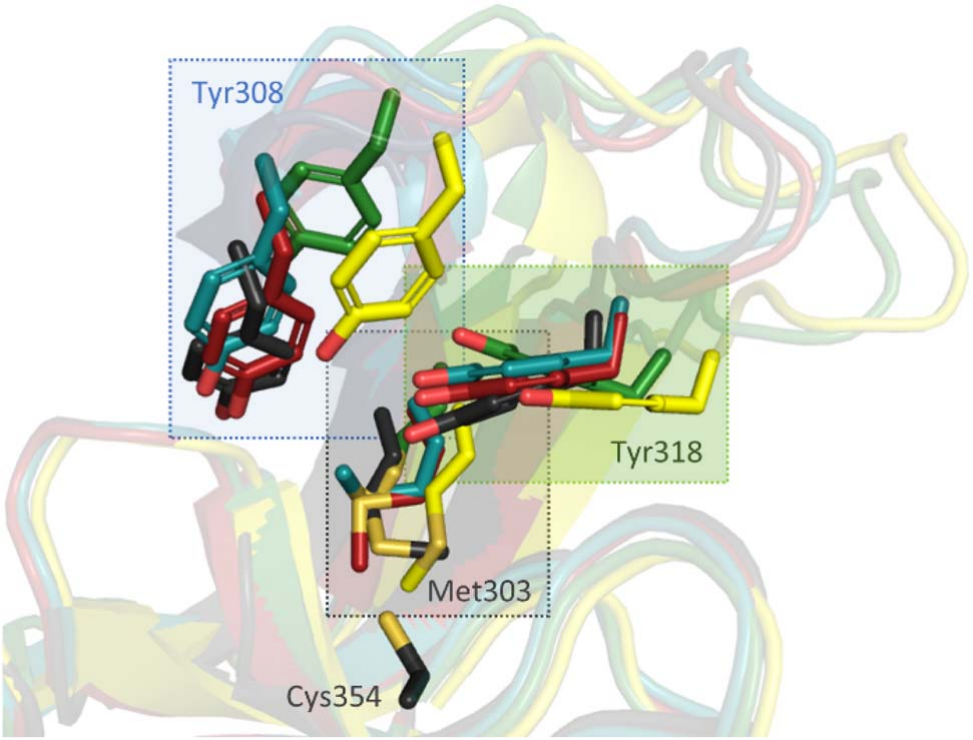
Inhibitor binding induces conformational changes in the active site “lid” (residues 300–323) of Ldt_Mt2_, resulting in reorientation of certain active site residues, in particular Met303, Tyr308, and Tyr318. Superimposition of unmodified Ldt_Mt2_ (dark grey, PDB 6RLG)^19^ and Ldt_Mt2_ reacted with 2 (yellow, PDB 8A1L), 4 (teal, PDB 8A1M), 8 (green, PDB 8A1O) and 13 (red, PDB 8A1N). Note that the conformation of Cys354 (shown only for unmodified Ldt_Mt2_) is very similar in each complex.

Overall, the Ldt_Mt2_ fold in the structures of the complexes strongly resembles that of unmodified Ldt_Mt2_ (PDB 6RLG;^19^ main chain RMSD: 0.54 Å, 0.37 Å, 0.45 Å, 0.86 Å, 0.7103 Å, and 0.77 Å, for unmodified Ldt_Mt2_ compared to Ldt_Mt2_ reacted with 2, 3, 4, 8, 13, and 15, respectively). As a notable exception, substantial variation of the position of the active site lid (residues 300–323) was observed, resulting in apparent reorientation of various active site residues involved in the binding of the inhibitors, in particular of Tyr308, Met303 and Tyr318 (Fig. 6).

**Fig. 7:**
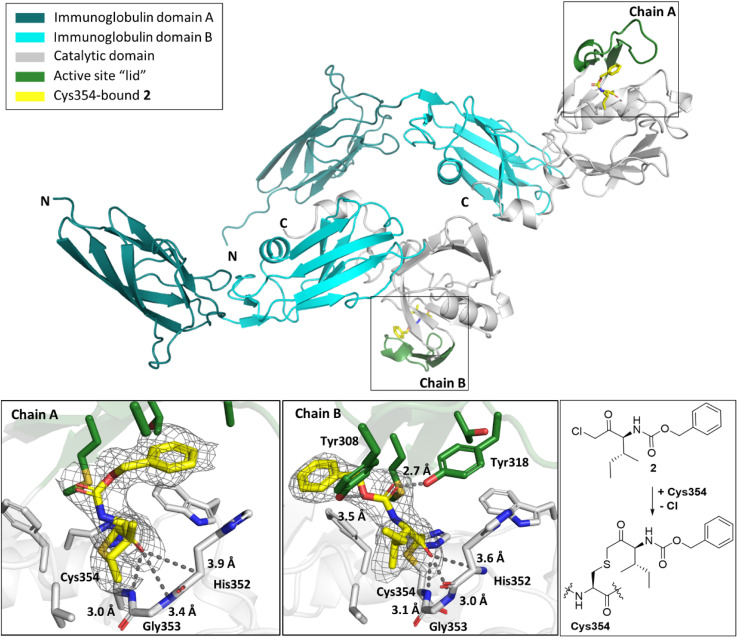
Views from a crystal structure derived by reaction of Ldt_Mt2_ with α-chloromethyl ketone 2 (yellow, PDB 8A1L). The immunoglobulin-like domains are in teal and cyan. The catalytic domain is grey, with the active site lid in green. Two different conformations of Ldt_Mt2_-2 were observed in chains A and B. *mF*_0_ − *DF*_c_ polder OMIT maps^59^ are contoured at 3.0*σ*, carved around Cys354 bound 2 (shown in grey mesh). Polar interactions are shown in dark grey dashes. The crystallographically assigned product is consistent with that observed by mass spectrometry (Fig. S10†).

While the overall folds of the two Ldt_Mt2_ molecules in the structure of the Ldt_Mt2_-2 complex overlay well (main chain RMDS: 0.72 Å), the reacted 2 is observed in two distinct binding modes (Fig. 7). In the conformation observed in chain B, the ester group of 2 is positioned to engage in polar interactions with the sidechains of Tyr308 (3.5 Å) and Tyr318 (2.7 Å). These residues are disordered in chain A, an observation which can be rationalised by a potential steric clash with the phenyl ring of 2 in chain A. In both binding modes, the carbonyl group of 2 is positioned in the oxyanion hole (the respective distances to the backbone nitrogens of His352, Gly353 and Cys354 in chain A are 3.9 Å, 3.4 Å and 3.4 Å and in chain B are 3.6 Å, 3.0 Å and 3.1 Å). The *sec*-butyl- and phenyl-groups of the inhibitor are oriented towards a hydrophobic pocket made up of the active site residues: Met303, Thr320, Val333, Phe334, His336, Trp340, and His352.

**Fig. 8:**
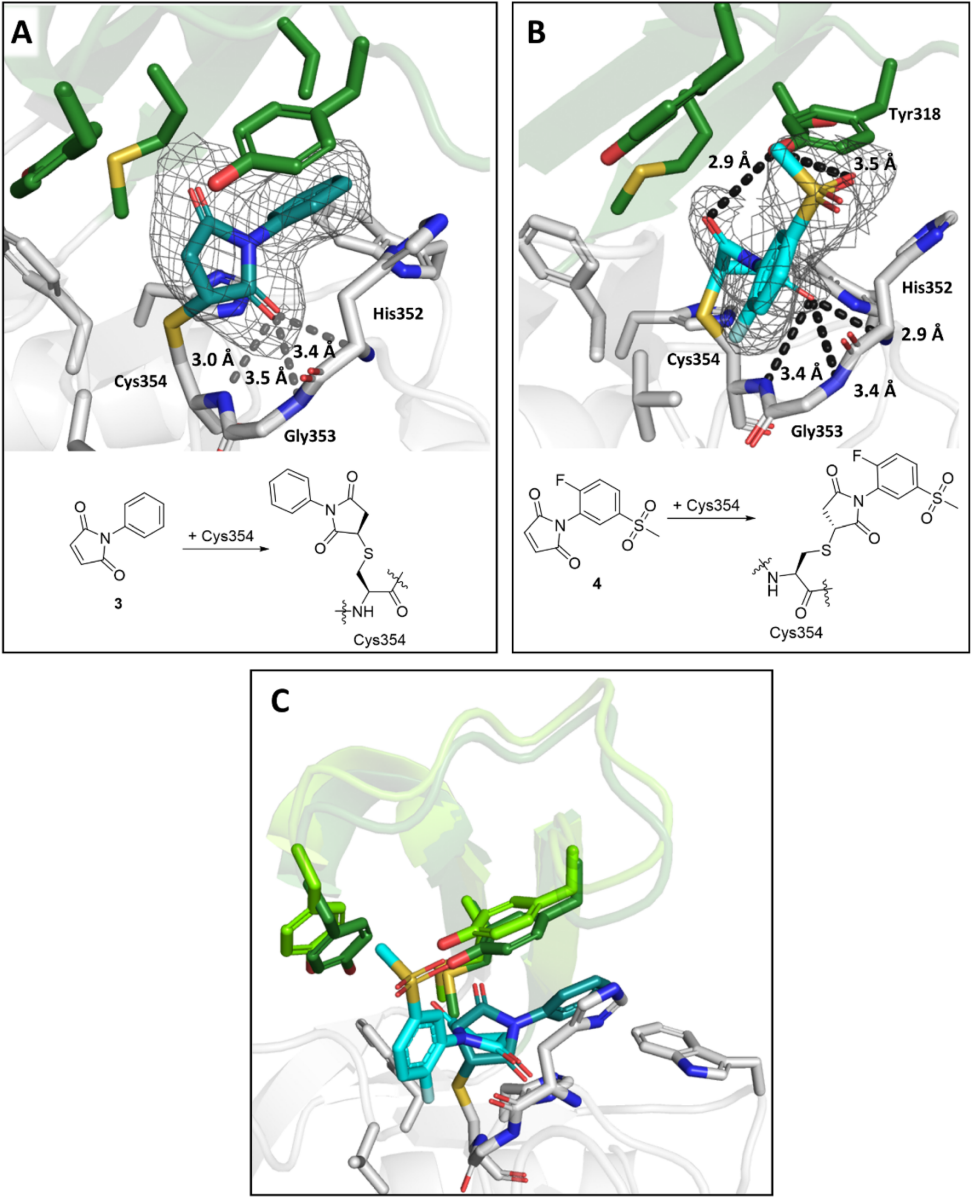
Views from crystal structures of the complex derived by reaction of Ldt_Mt2_ with maleimides 3 and 4. (A) Active site view of Ldt_Mt2_ reacted with 3 (teal; PDB 8A1J). (B) Active site view of Ldt_Mt2_ reacted with 4 (cyan; PDB 8A1M). (C) Superimposition of Ldt_Mt2_ complexes with 3 (teal) and 4 (cyan). Despite close structural similarity between 3 and 4, different conformations were observed, with variations in the positions of active site “lid” residues Met303, Tyr308 and Tyr318 (dark green for the Ldt_Mt2_-3 complex, light green for the Ldt_Mt2_-4 complex). Active site views from (A), (B) and (C) are shown as observed in chain A. *mF*_0_ − *DF*_c_ polder OMIT maps^59^ are contoured at 3.0*σ*, and carved around Cys354 bound 3 and 4. (shown in grey mesh). Polar interactions are shown in dark grey dashes. The crystallographically assigned products are consistent with those observed by mass spectrometry (Fig. S10†).

The maleimides 3 and 4 are structurally related, but their binding modes differ (Fig. 8). While the sulfone group on the *N*-phenyl ring of 4 engages in polar interactions with Tyr318 (3.5 Å, Fig. 8B), the *N*-phenyl ring of 3 is positioned for hydrophobic interactions with Met303, Tyr318, Thr320, and Trp340 (Fig. 8A). In both binding modes, one of the maleimide carbonyls is positioned in the oxyanion hole (the respective distances to the backbone nitrogens of His352, Gly353 and Cys354 for 3 are 3.4 Å, 3.5 Å and 3.0 Å and for 4 are 2.9 Å, 3.4 Å and 3.4 Å). Additional polar interactions were observed between the second maleimide carbonyl of 4 and the phenolic OH of Tyr318 (2.9 Å). Despite differences in their binding modes, both maleimides bind to Ldt_Mt2_ with the same stereochemical outcome, that is forming the (*R*)-enantiomer.

**Fig. 9:**
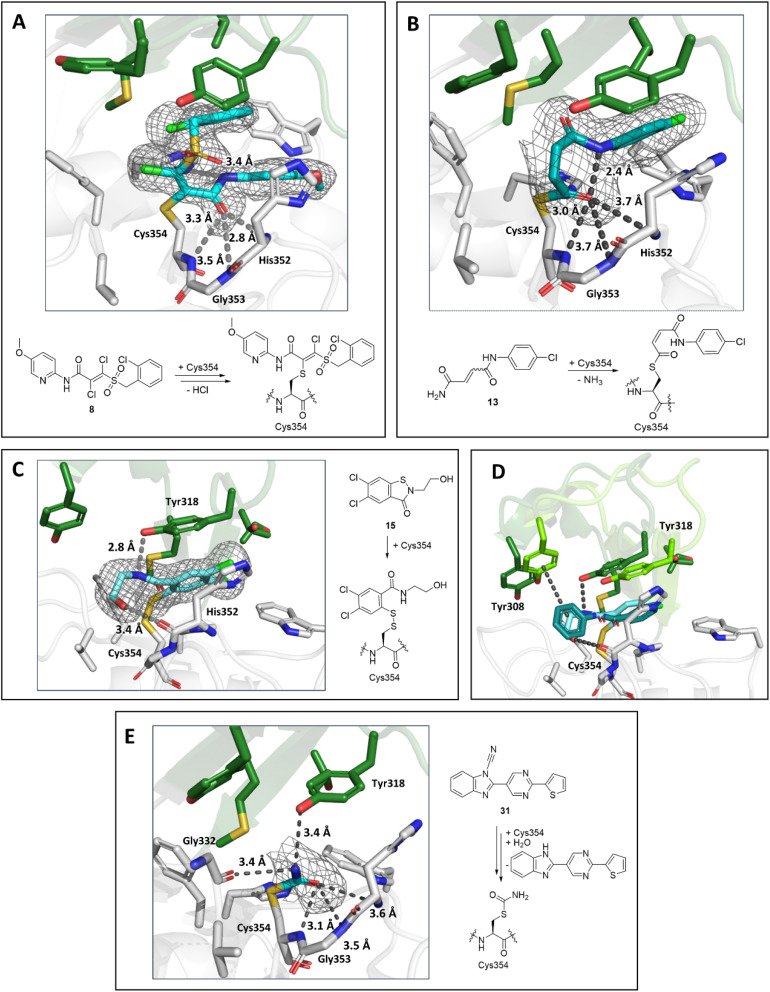
Views from crystal structures of the complex derived by reaction of Ldt_Mt2_ with inhibitors 8, 13, 15 and 31. (A) Active site view of Ldt_Mt2_ reacted with 8 (cyan, PDB 8A1O). (B) Active site view of Ldt_Mt2_ reacted with 13 (teal, PDB 8A1N). (C) Active site view of Ldt_Mt2_ reacted with 15 (cyan; PDB 8A1K). (D) Superimposition view from crystal structures of Ldt_Mt2_ in complex with 15 (Ldt_Mt2_-bound 15 in cyan, active site “lid” in dark green; PDB 8A1K) and in complex with ebselen (Ldt_Mt2_-bound ebselen in teal, active site “lid” in light green; PDB 6RRM).^19^. (E) Active site view of Ldt_Mt2_ reacted with 31 (cyan; PDB 8AHO). Active site views in (A–E) are shown as observed in chain A. *mF*_0_ − *DF*_c_ polder OMIT maps^59^ contoured at 3.0*σ*, carved around Cys354 bound 8, 13, 15 and 31 are shown in grey mesh. Polar interactions are shown in grey dashes. The crystallographically assigned products are consistent with those observed by mass spectrometry (Fig. S10 and S13†).

By contrast with the other Ldt_Mt2_ structures reported here, a crystal structure of Ldt_Mt2_ in complex with acrylamide 8 was solved in the *P*2_1_2_1_2 space group with a single molecule in the ASU. Inhibitor 8 contains an asymmetric Michael acceptor warhead and has the potential to covalently react with Ldt_Mt2_ either *via* Michael addition to its acrylamide or *via* reaction of its vinyl sulphone group. The obtained structure implies that Ldt_Mt2_ reacts regioselectively by Michael reaction with the vinyl sulphone of 8 (Fig. 9A). Polar interactions were observed between the amide carbonyl and backbone nitrogen of His352 (2.8 Å) and the amide nitrogen and the side chain of His336 (3.4 Å). The amide carbonyl of 8 is positioned in the oxyanion hole (the respective distances to the backbone nitrogens of His352, Gly353 and Cys354: 2.8 Å, 3.5 Å and 3.5 Å).

A structure of Ldt_Mt2_ reacted with 13 manifests clear additional electron density at the Ldt_Mt2_ active site in chain A and partial density in chain B. 13 was thus only modelled in chain A. Notably, analysis of the electron density in chain A showed that, in accord with the SPE-MS experiments, 13 covalently modifies Ldt_Mt2_ through reaction of Cys354 with its terminal amide, rather than by Michael reaction with its fumaryl group (Fig. 9B). Whilst in solution 13 exists as a mixture of *E*/*Z* isomers, the obtained structure implies (at least predominantly) selective reaction of Ldt_Mt2_ with the (*Z*)-stereoisomer of 13. The nitrogen of 13 is positioned for polar interaction with the Cys354-13 acyl (2.4 Å), and the Cys354-13 acyl carbonyl group is positioned in the oxyanion hole (the respective distances of the inhibitor carbonyl oxygen to the backbone nitrogens of His352, Gly353 and Cys354: 3.7 Å, 3.7 Å and 3.0 Å).

A structure of Ldt_Mt2_ reacted with the ebsulfur analogue 15 revealed the inhibitor covalently bonded to Cys354 *via* reaction of the N–S bond, analogous to the reaction of Ldt_Mt2_ with ebselen.^19^ The inhibitor hydroxy group of the species is positioned to engage in hydrogen-boding interactions with the backbone carbonyl of His352 (3.4 Å) and the inhibitor amide with the sidechain of Tyr318 (2.8 Å, Fig. 9C). Comparison of the binding mode of 15 with that of ebselen (PDB 6RRM),^19^ implies that while both compounds are positioned similarly in the active site, the phenyl ring of ebselen is positioned for π-interactions with Tyr318, while the hydroxy group of 15 is positioned for polar interactions with the backbone carbonyl of His352 (Fig. 9D), accompanied by reorientation of the active site lid.

A crystal structure of Ldt_Mt2_ reacted with cyanamide 31 manifested covalent modification of Cys354 with formation of a carbamoyl group, likely resulting from initial formation of cyanated cysteine followed by hydrolysis (Fig. 9E). The carbamoyl group was directed towards the oxyanion hole (the respective distances of the carbonyl oxygen to the backbone nitrogens of His352, Gly353 and Cys354: 3.6 Å, 3.5 Å and 3.1 Å). Further polar interactions were observed between the nitrogen of the carbamoyl group and the sidechain of Tyr318 (3.4 Å in chain A) and the backbone carbonyl of Gly332 (3.4 Å in chain A).

### Selected hit compounds are active in *M. tuberculosis* residing in macrophages

The hit compounds were evaluated for inhibition of *M. tuberculosis*, both in medium under replicating conditions and whilst residing in macrophages. The results reveal α-chloro ketone 1, maleimides 5 and 7, ebsulfur analogue 15, isatins 17 and 18, β-lactam 20, cyanamides 23, 30, 36 and nitrile 39 as inhibitors of *M. tuberculosis* residing in macrophages, with MIC_50_ values <50 μM (MIC_50_ values represent the concentration of inhibitor that inhibited bacterial growth by 50%, as obtained from extrapolation of the dose–response curve). None of the tested compounds showed inhibition >100 μM when assessed in medium under replicating conditions (Table S12†). The most potent inhibitor was nitrile 30 (MIC_50_ 0.98 μM), followed by ebsulfur analogue 15 (MIC_50_ 7.08 μM), cyanamide 23 (MIC_50_ 12.0 μM) and isatin 17 (MIC_50_ 15.1 μM). The MIC_50_ values of the remaining compounds ranged between 25.7–39.8 μM. However, cytotoxicity was observed in HepG2 cells (IC_50_ 2.51–53.5 μM) for all compounds shown in Table S12,† apart from β-lactam 20 and nitrile 39 (IC_50_ > 100 μM).

## Discussion

Ldt_Mt2_ is reported to be essential for the virulence of *M. tuberculosis*,^10^ and is mechanistically and functionally related to bacterial PBPs, which are the targets of probably the most important class of all clinically used antibacterials, that is the β-lactams. Ldt_Mt2_ is thus of considerable interest as a drug target for the treatment of *M. tuberculosis* infections.

The results of our HTS for inhibitors of Ldt_Mt2_ with ∼10 000 electrophilic compounds identified multiple classes of compounds that react covalently with Ldt_Mt2_, that is: α-chloro ketones, maleimides, acrylamides, fumaryl amides, an ebsulfur analogue, isatins, nitriles (among which a subset of cyanamides), and β-lactams. Several of these functional groups are present in human drugs.^48^ The results show that unexpected reaction modes can occur as observed, amongst other compounds, for the fumaryl amides 13 and 14. Among the most potent identified Ldt_Mt2_ inhibitors were the ebsulfur analogue 15 (pIC_50_ 7.99) and the cyanamides 22–36 (pIC_50_ 6.39–7.53).

In most cases inhibition can be related to irreversible covalent reaction with the nucleophilic Cys354, as evidenced by protein observed MS analyses, with an exception being the isatins, which likely react *via* a reversible covalent mechanism. X-ray crystallographic results suggests that, in at least some cases, induced fit occurs during inhibitor binding. In particular, differences in the conformation of the loop that borders the active site (residues 300–323) are observed, leading to variations in the positions of some active site residues (in particular Tyr308, Tyr318 and Met303) that modulate both polar and hydrophobic interactions with the inhibitors. These observations are in accord with previous observations regarding changes in the conformation of the flexible loop upon binding of β-lactams and ebselen.^13,14,19^ At present, the exact relationship between inhibitor reactions and binding and changes in the conformation of the flexible loop and related residues remain unclear. However, the previous reported structural data^13,14,19^ combined with data reported here forms a basis for detailed biophysical and modelling studies to understand the role of the flexible loop in Ldt_Mt2_ catalysis and inhibition.

Efforts to inhibit Ldt_Mt2_ have thus far focussed on inhibition by β-lactams, in particular by the carbapenem subclass.^14–18^ In our HTS campaign we aimed to build on the observation that Ldt_Mt2_ can be inhibited by alternative types of cysteine-reactive compounds.^19^ Indeed, the majority of hit compounds that we identified are non-β-lactam covalent inhibitors of Ldt_Mt2_. Importantly, we identified Ldt_Mt2_ inhibitors from the cephalosporin subclass of β-lactams, which have previously been deemed to be ineffective in the inhibition of Ldt_Mt2_.^23,27^ The identified cephalosporins manifested pIC_50_ values comparable to the observed potencies of carbapenems such as meropenem, doripenem and biapenem (which were included in the HTS as control compounds). This is a notable observation, given that cephalosporins are very widely used antibacterials that are, unlike the intravenously used carbapenems, orally active.^49^

Of the Ldt_Mt2_ inhibitor classes identified here, the cyanamides 22–36 are of particular mechanistic interest, as they are electrophilic agents that cyanate Cys354 with high inhibitory potency. The cyanamide series exhibit increased reactivity over the reported cyanating agents 40–45,^34,35,38,39^ whilst most derivatives remain selective for reaction with Cys354. Interestingly, in some cases we observed hydrolysis of the *S*-nitrile group to give an *S*-carbamoyl group, as validated by crystallography in the case of 31. Additionally, we observed cleavage of Ldt_Mt2_ following *S*-cyanation. Such cleavage likely occurs *via* 5-*exo*-dig cyclisation of the Cys354 amide nitrogen onto the carbon of the *S*-nitrile group, followed by hydrolysis on the *N*-terminal side of the cysteine residue.^50^ Although not the focus of our work, the results presented here suggest that developing cyanamide type reagents for cleavage at cysteine residues in proteins should be possible. Although it may be challenging to develop cyanamides as drugs due to their reactivity, we also identified nitrile inhibitors (38, 39). The potential of nitriles for the inhibition of nucleophilic cysteine enzymes is highlighted by the recent development of cysteine reacting inhibitor of the main protease (M^pro^) of SARS-CoV-2.^21^

The HTS approach is validated by the observation that out of the 39 selected hit compounds, 11 were found to have a bactericidal effect on *M. tuberculosis* in macrophages. The most potent identified compound was the cyanamide 30 (MIC_50_ of 0.98 μM). Due to environmental factors, such as phagosomal acidification and presence of reactive nitrogen species, *M. tuberculosis* residing in macrophages is typically in the nonreplicating or replication-inhibited phase.^51–53^ The observed bactericidal effect of the selected hit compounds on *M. tuberculosis* within macrophages, but not *M. tuberculosis* under replicating conditions, is thus in accord with the anticipated behaviour of Ldt_Mt2_ inhibition, which is proposed to be essential during the nonreplicating stage.^9,10^ However, further research is necessary to confirm the mechanism of action of these compounds.

## Conclusion

The overall results reveal novel classes of electrophilic inhibitors for the promising anti-TB target Ldt_Mt2_. They provide mechanistic data on the modes of inhibition, so providing leads for the development of potent and selective Ldt_Mt2_ inhibitors. Amongst the inhibitor classes identified, the cephalosporins, the nitriles and the isatins are of particular interest due to their chemical interest and bactericidal effect on *M. tuberculosis* residing in macrophages. The results also reveal the power of HTS followed by subsequent protein observed MS analysis focussed on electrophiles to unveil new classes of inhibitors of Ldt_Mt2_ and potentially other classes of nucleophilic enzymes. This aspect of the results, as exemplified by the cyanamides, suggests that there is considerable potential for identifying new types of electrophilic warheads for inhibition of nucleophilic enzymes.

## Methods

### Materials

Recombinant Ldt_Mt2_ and BlaC were produced in *E. coli* and purified to <95% purity by SDS-PAGE as described.^54^ Probes 1 and 2 and FC-5 were synthesised as reported.^23,26^ The HTS library of ∼10 000 electrophilic compounds was constructed from the GSK compound collection of ∼4 million compounds, by means of 21 different substructure searches using Smart Queries, and a molecular weight filter (MW < 450). Substructures consisting of known, or potential cysteine, binding warheads were identified from literature searches, and datasets from other cysteine and serine protease programmes within GSK.

### Ldt_Mt2_ fluorogenic assays

Compounds (in DMSO) were dispensed into a black polystyrene, flat-bottomed, small volume, clear bottomed 384-well microplate (Greiner Bio-One, part number 784076). Single shot assays were performed at a compound concentration of 10 μM (100 nL at 10 mM) and dose–response assays were performed in concentrations ranging from 100 μM to 1.69 nM (11 dilutions of factor 3). Two types of controls were used. In control 1, inhibitors were substituted with neat DMSO (100 nL) (expected 0% inhibition; 100% fluorescence signal). In control 2, the addition of Ldt_Mt2_ was omitted (expected 100% inhibition; 0% fluorescence signal). Ldt_Mt2_ (final concentration 300 nM) in assay buffer (50 mM sodium phosphate, pH 7.5, 0.007% (v/v) Tween-20) was added to all wells (except control 2) using a MultiDrop Combi dispenser (ThermoFisher Scientific). In the case of control 2, the Ldt_Mt2_ mixture was substituted with the assay buffer. This mixture was incubated for 30 minutes at room temperature without shaking. Probe 1 (final concentration 15 μM, final assay volume 10 μL) was added using a MultiDrop Combi dispenser (ThermoFisher Scientific). The plate was incubated for 10 minutes at room temperature. The fluorescence intensity was then measured every 5 minutes for 50 minutes using an Envision 2104 Multilabel Reader (PerkinElmer) with *λ*_ex_ = 485 nm, *λ*_em_ = 540 nm and an FITC mirror. Data were analysed using ActivityBase (IDBS).

### BlaC fluorogenic assays

Compounds (in DMSO, concentrations ranging from 100 μM to 1.69 nM) were dispensed into a black polystyrene, flat-bottomed, small volume, clear bottomed 384-well microplate (Greiner Bio-One, part number 784076). Two types of controls were used. In control 1, inhibitors were substituted with neat DMSO (100 nL) (expected 0% inhibition; 100% fluorescence signal). In control 2, the addition of BlaC was omitted (expected 100% inhibition; 0% fluorescence signal). BlaC (final concentration 2.5 nM) in assay buffer (100 mM sodium phosphate pH 7.5 with 0.01% (v/v) Triton X-100) was added to all wells (except control 2) using a MultiDrop Combi dispenser (ThermoFisher Scientific). In control 2, the BlaC mixture was substituted with the assay buffer. This mixture was incubated for 30 minutes at room temperature without shaking. FC5 (final concentration 2.5 μM, final assay volume 10 μL) was added using a MultiDrop Combi dispenser (ThermoFisher Scientific). The fluorescence intensity was measured every minute for 10 minutes using an Envision 2104 Multilabel Reader (PerkinElmer) with *λ*_ex_ = 340 nm, *λ*_em_ = 480 nm and a general dual mirror. Data were analysed using ActivityBase (IDBS).

### HTS interference assays

Compounds (in DMSO, concentrations ranging from 100 μM to 1.69 nM) were dispensed into a black polystyrene, flat-bottomed, small volume, clear bottomed 384-well microplate (Greiner Bio-One, part number 784076). Two types of controls were used. In control 1, inhibitors were substituted with neat DMSO (100 nL) (expected 0% inhibition, 100% fluorescence signal). In control 2, the addition of Ldt_Mt2_ was omitted (expected 100% inhibition, 0% fluorescence signal). Prior to the assay, Ldt_Mt2_ (final concentration 300 nM) was reacted with probe 1 (final concentration 15 μM) in assay buffer (50 mM sodium phosphate, pH 7.5, 0.007% (v/v) Tween-20) for 5 hours at room temperature. To all wells except control 2, 10 μL of the reacted Ldt_Mt2_ and the probe 1 mixture were added, using a MultiDrop Combi dispenser (ThermoFisher Scientific). In control 2, the Ldt_Mt2_ mixture was substituted with 5 μL of the assay buffer. The plate was incubated for another 10 minutes at room temperature. The fluorescence intensity was then measured every 5 minutes for 50 minutes using an Envision 2104 Multilabel Reader (PerkinElmer) with *λ*_ex_ = 485 nm, *λ*_em_ = 540 nm and an FITC mirror. Data were analysed using ActivityBase (IDBS).

### Protein observed solid phase extraction mass spectrometry

Ldt_Mt2_ (1 μM) in buffer (50 mM tris, pH 7.5) was incubated with an inhibitor (100 μM, 10 μM, or 2 μM, as indicated). Mass spectrometry was performed using a RapidFire200 integrated autosampler/solid phase extraction (SPE) system (Agilent Technologies) coupled to an API40000 triple quadrupole mass spectrometer (Applied Biosystems) operating in the positive ionisation mode. Samples were taken after the specified incubation periods, loaded onto a C4 cartridge (Agilent Technologies), and eluted with organic phase (85% (v/v) acetonitrile, 15% (v/v) water, 0.1% (v/v) formic acid). The cartridge was washed with both the organic phase and an aqueous phase (water, 0.1% (v/v) formic acid). Data were analysed using MassHunter Qualitative Analysis B.07.00 (Agilent Technologies). Spectra were deconvoluted using the maximum entropy algorithm in the same programme.

### Rate constant of covalent target inactivation (*k*_inact_/*K*_I_)

The second-order rate constant for irreversible inhibition (*k*_inact_/*K*_I_) was determined using irreversibly reacting probe 2.^41^ The *k*_inact_/*K*_I_ of probe 2 was determined through measurement of the fluorescence intensity of the reaction between Ldt_Mt2_ (100 nM) and probe 2 (concentrations ranging between 100 nM and 10 μM) in 50 mM HEPES, pH 7.2, 0.01% (v/v) Triton X-100 using a PHERAstar plate reader (BMG Labtech) with *λ*_ex_ = 480 nm and *λ*_em_ = 520 nm. The observed kinetic rate constant (*k*_obs_) was determined using eqn (1).

1 *Y* = (*Y*_∞_ − *Y*_0_)[1 − e^(−*k*_obs_×*t*)^] + *Y*_0_

The (*k*_inact_/*K*_I_)_probe_ was derived from *k*_obs_ using eqn (2), yielding a value of 39.8 M^−1^ s^−1^.

2 *k*_obs_ = (*k*_inact_/*K*_I_)_probe_[probe **2**]

The half-life (*t*_1/2_) of probe 2 (10 μM), was determined using eqn (3) to be 28.7 min. Therefore, an incubation time of 2.4 h should ensure <97% receptor occupancy. With this knowledge, an incubation time of 3 h was selected for the determination of (*k*_inact_/*K*_I_)_inhibitor_ values.

3 *t*_1/2_ = ln 2/*k*_obs_

Ldt_Mt2_ (100 nM) added to a mixture of varying concentrations of inhibitor (400 μM to 20.3 nM) for 10 min in assay buffer (50 mM HEPES, pH 7.2, 0.01% (v/v) Triton X-100) and probe 2 (10 μM), and incubated for 3 h prior to determination of the fluorescence intensity using a PHERAstar plate reader (BMG Labtech) with *λ*_ex_ = 480 nm and *λ*_em_ = 520 nm. Dose–response analyses were performed using a variable slope nonlinear regression model in GraphPad Prism 9 (GraphPad Software). The (*k*_inact_/*K*_I_)_inhibitor_ values were derived from the IC_50_ values using eqn (4).4
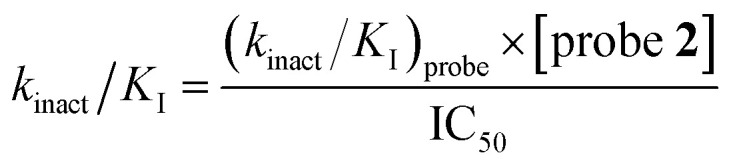


### Intrinsic thiol reactivity

In order to obtain the rate constant of probe 2 for reaction with l-glutathione (*k*_probe_), l-glutathione (final concentration 500 nM) and probe 2 (final concentration 10 μM) in the assay buffer (50 mM HEPES pH 7.2, 0.01% (v/v) Triton X-100) were added to a 384-well plate. The fluorescence signal was measured using a PHERAstar plate reader (BMG Labtech) with *λ*_ex_ = 480 nm and *λ*_em_ = 520 nm. Readings were taken until apparent completion of the reaction (15 hours). The signal was fitted with a single exponential curve using GraphPad Prism 9 (GraphPad Software) to give the observed kinetic rate constant (*k*_obs_), which was then converted to *k*_probe_ using eqn (5).

5 *k*_obs_ = *k*_probe_[probe **2**]

Inhibitors were serially diluted (by 1/3) across columns 2–10 of a 96-well plate (starting concentration 10 mM). The final two columns of the microplate (11 and 12) were used as inhibitor-free controls and contained only DMSO. To a 384-well plate was added assay buffer (14 μL) using a MultiDrop Combi dispenser (ThermoFisher Scientific). The inhibitor or blank (1 μL) solutions were added using a CyBio liquid handling system (Analytik Jena AG), with 4 replicates per inhibitor concentration. To each well was added probe 2 (5 μL, final concentration 10 μM) using a MultiDrop Combi dispenser (ThermoFisher Scientific). l-Glutathione (5 μL, final concentration 500 nM, final volume 25 μL) was added and the plate was sealed and incubated for 15 h at room temperature. The fluorescence signal was measured using a PHERAstar plate reader (BMG Labtech) with *λ*_ex_ = 480 nm and *λ*_em_ = 520 nm and normalised against the mean average of no inhibitor controls and the mean average of no enzyme controls. Nonlinear regression analyses of dose–response were conducted using GraphPad Prism 9 (GraphPad Software) with a variable slope model. The *k*_chem_ of inhibitors was calculated from the obtained IC_50_ value using eqn (6).6
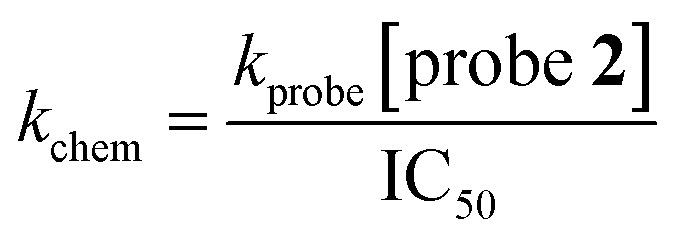


### Differential scanning fluorimetry (DSF) thermal shift assays

Ldt_Mt2_ (5 μM) was incubated with the inhibitor (500 μM) in assay buffer (50 mM tris pH 7.5) for 30 minutes at room temperature in 96-well white PCR plates (25 μL final volume, Starlab). SYPRO Orange (final concentration 6×, according to manufacturer's definition, Invitrogen) was then added and the plate was sealed using a transparent plastic film (Polyolefin StarSeal, Starlab). Fluorescence was monitored (with *λ*_ex_ = 492 nm and *λ*_em_ = 610 nm) using a CFX96 Touch Real-Time PCR Detection System (Bio-Rad), while the temperature was increased from 20 to 95 °C in increments of 0.2 °C. Melting temperatures (*T*_m_s) were determined using the CFX Maestro Software (Bio-Rad).

### Jump dilution assays

Ldt_Mt2_ (10 μM final concentration) was incubated with the inhibitor (100 μM final concentration) in the assay buffer (50 mM HEPES, pH 7.2, 0.01% (v/v) Triton X-100) for 2 hours at room temperature, then serially diluted 1000× with assay buffer (final Ldt_Mt2_ concentration 10 nM). 20 μL of the diluted mixture was added to a black polystyrene, flat-bottomed 384-well μ-clear plate (clear bottomed, Greiner Bio-One, part number 781096). Probe 2 (final concentration 15 μM) was added using a MultiDrop Combi dispenser (ThermoFisher Scientific). Fluorescence was measured using a PHERAstar plate reader (BMG Labtech) with *λ*_ex_ = 480 nm and *λ*_em_ = 520 nm. Readings were taken every 15 s for a period of 5 minutes, using bottom optic measurements. The off rate (*k*_off_) was determined using eqn (7).7
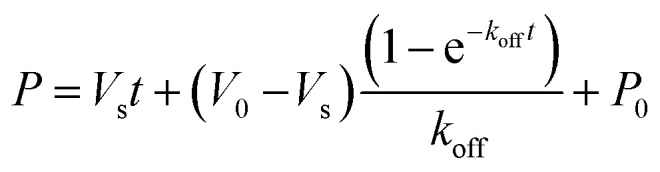
where, *P* = formed product; *V*_s_ = velocity of no-inhibitor control; *V*_0_ = velocity of no-enzyme control, and *P*_0_ = initial fluorescence.

The half-life of the enzyme–inhibitor complex (*t*_1/2_) was calculated using eqn (8).8
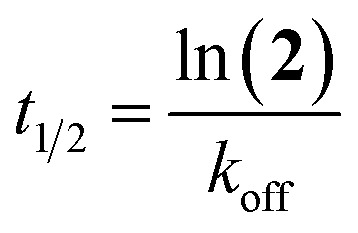


### Nondenaturing MS assays

Protein samples were prepared by buffer exchange into 500 mM ammonium acetate by dialysis using a Slide-A-Lyser 0.5 mL Dialysis Cassette (Thermo Scientific). Ldt_Mt2_ (final concentration 5 μM) was combined with the inhibitors of interest (final concentration 50 μM). Mass spectra were acquired using a Waters Synapt Q-TOF machine operating in the positive ionisation mode with extended *m*/*z* range connected to an Advion Triversa Nanomate chip-based electrospray ionisation and autosampler. The cone voltage was as specified, and spectra were accumulated over approximately 50 scans. Data were analysed using MassLynx v4.1 (Waters).

### X-ray crystallography

Ldt_Mt2_ (Δ1-55; with the N-terminal HisTag removed, in 50 mM tris, pH 8.0, 100 mM NaCl) was crystallised according to a modified reported procedure.^19^ The well solution was composed of 0.2 M ammonium nitrate and 20% (w/v) PEG 3350. Sitting drop vapor diffusion crystallization plates (low reservoir Intelli-Plate 93–3) were prepared using a Rigaku Phoenix RE Drop setter instrument (Art Robbins Instrument). Crystallisation plates were stored at 4 °C. Inhibitors were introduced through soaking. The crystals were then mounted on nylon loops, cryocooled and stored in liquid nitrogen. Datasets were collected using the MX beamline i03 at Diamond Light Source. Structures were solved by molecular replacement with Phaser,^55^ using PDB entry 6RRM,^19^ as the search model. Alternating cycles of refinement using PHENIX,^56^ and manual model building using COOT,^57^ were performed until *R*_work_ and *R*_free_ converged. Data collection and refinement statistics are given in Table S13.†

### Bacterial strain

*M. tuberculosis* H37Rv cells expressing the luciferase *luc* gene from *Photinus pyralis* (GenBank Accession Number M15077) inserted in a mycobacterial shuttle plasmid derived from pACE-1 (ref. 58) (Mtb H37Rv-Luc) was routinely propagated at 37 °C in Middlebrook 7H9 broth (Difco) supplemented with 10% Middlebrook albumin-dextrose-catalase (ADC, Difco), 0.2% glycerol and 0.05% (v/v) tyloxapol or on Middlebrook 7H10 agar plates (Difco) supplemented with 10% (v/v) oleic acid-albumin-dextrose-catalase (OADC, Difco). Hygromycin B was added to the medium (50 μg mL^−1^) to ensure plasmid maintenance when propagating Mtb H37Rv-Luc.

### Intracellular assays

Frozen stocks of macrophage THP-1 cells (ATCC TIB-202) were thawed in RPMI-1640 medium (Sigma) supplemented with 10% fetal bovine serum (FBS, Gibco), 2 mM l-glutamine (Sigma) and 1 mM sodium pyruvate (Sigma). THP-1 cells were passaged only 5 times and maintained without an antibiotic between 2–10 × 105 cells per mL at 37 °C in a humidified, 5% CO_2_ atmosphere. THP-1 cells (3 × 108) were simultaneously differentiated with phorbol myristate acetate (PMA, 40 ng mL^−1^, Sigma) and infected for 4 hours at a multiplicity of infection (MOI) of 1 : 1 with a single cell suspension of Mtb H37Rv-Luc cells. After incubation, infected cells were washed four times to remove extracellular bacilli and resuspended (2 × 105 cells per mL) in RPMI medium supplemented with 10% FBS (Hyclone), 2 mM l-glutamine and pyruvate and dispensed in white, flat bottom 384-well plates (Greiner) in a final volume of 50 μL containing test compounds (11 serial dilutions of 1/3 with starting concentration 100 μM, max. 0.5% DMSO). Plates were incubated for 5 days under 5% CO_2_ atmosphere, 37 °C, 80% relative humidity before growth assessment. The Bright-Glo™ Luciferase Assay System (Promega, Madison, WI) was used as cell growth indicator for the Mtb H37Rv-Luc strain. Luminescence was measured in an Envision Multilabel Plate Reader (PerkinElmer) using the opaque 384-plate Ultra-Sensitive luminescence mode, with a measurement time of 50 ms. A reduction in light production was considered growth inhibition and the IC_50_ or IC_90_ value was interpolated from the dose response curve.

### Extracellular assays

*M. tuberculosis* H37Rv cells were cultured in Middlebrook 7H9 medium supplied with 10% (v/v) ADC and 0.025% (v/v) Tyloxapol, then incubated at 37 °C for approximately 10 days. Following a purity check, subculture was performed in Middlebrook 7H9 medium supplied with 10% (v/v) ADC and 0.025% (v/v) Tyloxapol up to OD_600_ = 0.01 and incubated at 37 °C for 4–6 days. The inoculum was adjusted to OD_600_ = 0.00125 (equivalent to 1 × 105 cfu mL^−1^). 50 μL of the inoculum was dispensed in all wells of a 384-well plate, containing test compounds (11 serial dilutions of 1/3 with starting concentration 100 μM). Plates were placed in a sealed box and incubated at 37 °C for 8 days. The lids were then removed, and the plates were covered with a seal. Absorption was measured at 590 nm using an Envision 2104 Multilabel Reader (PerkinElmer). If the window between negative control (Rifampicin) and positive control (no inhibitor) was not greater than or equal to 3 times, plates were incubated at 37 °C for one or two additional days.

### HepG2 cytotoxicity assays

Solutions of the test compounds (250 nL per well; 11 serial dilutions of 1/3 with starting concentration 100 μM) were dispensed in tissue-culture treated black clear-bottomed 384-well plates (Greiner, cat. no. 781091) with an Echo 555 instrument. 25 μL of HepG2 (ATCC HB-8065) cells (∼3000 cells per well) grown to confluency in Eagle's Minimal Essential Medium (MEM) supplemented with 10% (v/v) heat-inactivated FBS, 1% (v/v) non-essential amino acids (NEAA), and 1% (v/v) penicillin/streptomycin were added to each well. Plates were incubated at 37 °C with 20% O_2_ and 5% CO_2_ for 48 h. The plates were then equilibrated to room temperature. ATP levels, measured with CellTiter Glo kit (Promega), were used as cell viability read-out. 25 μL of CellTiter Glo substrate dissolved in the buffer was added to each well. Plates were incubated at room temperature for 10 min and read on ViewLux (PerkinElmer) with excitation and emission filters of 613 and 655 nm, respectively. The HepG2 IC_50_ value corresponds to the concentration of the compound necessary to inhibit 50% of cell growth.

## Data availability

Crystallographic data has been deposited at the PDB under accession codes 8A1L, 8A1J, 8A1M, 8A1O, 8A1N, 8A1K and 8AHO. Additional figures and tables can be found in the ESI.†

## Author contributions

M. d. M. conceptualization, methodology, formal analysis, investigation, writing – original draft, writing – review & editing, visualization. P. A. L. conceptualization, methodology, investigation, writing – review & editing. F. D. D. A. conceptualization, methodology, investigation. M. C. conceptualization, methodology. R. B. conceptualization, supervision. J. B. conceptualization, methodology, supervision. B. R. M. conceptualization, methodology, data curation, supervision. C. J. S. conceptualization, methodology, supervision, writing – review & editing.

## Conflicts of interest

There are no conflicts to declare.

## Supplementary Material

SC-014-D2SC06858C-s001
