# Supplementary material for: High-throughput screen with the l,d-transpeptidase LdtMt2 of Mycobacterium tuberculosis reveals novel classes of covalently reacting inhibitors
Source: Chem Sci. 2023 May 30;14(26):7262–78. doi: 10.1039/d2sc06858c (PMC10321483; doi:10.1039/d2sc06858c)
Supplement: SC-014-D2SC06858C-s001 [file SC-014-D2SC06858C-s001.pdf]

Electronic Supplementary Material (ESI) for Chemical Science.
This journal is © The Royal Society of Chemistry 2023

Supplementary information

High-Throughput Screen with the L,D-transpeptidase Ldt_{Mt2} of *Mycobacterium tuberculosis* Reveals Novel Classes of Covalent Inhibitors

Mariska de Munnik[1], Pauline A. Lang[1], Francisco De Dios Anton[2], Monica Cacho[2], Robert Bates[2], Jürgen Brem[1, 3], Beatriz Rodríguez Miquel[2*] and Christopher J. Schofield[1*]

[1] Chemistry Research Laboratory, Department of Chemistry and the Ineos Oxford Institute of Antimicrobial Research, University of Oxford, 12 Mansfield Road, Oxford, OX1 3TA, United Kingdom.

[2] Tres Cantos Medicines Development Campus GlaxoSmithKline, Calle Severo Ochoa 2, Tres Cantos, Madrid, Spain.

[3] Present address: Janssen Research & Development, Janssen Pharmaceutica, B-2340 Beerse, Belgium.

[*] Email: beatriz.rodriguez@gsk.com and christopher.schofield@chem.ox.ac.uk

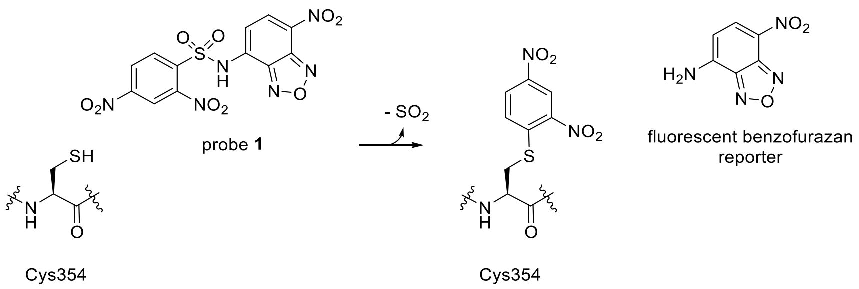

Figure S1. Basis of the high-throughput screen assay for $\mathbf{Ldt_{Mt2}}$ inhibitors. Reaction of the Ldt_{Mt2} nucleophilic Cys354 with probe **1** releases a fluorescent benzofurazan reporter molecule.[1]

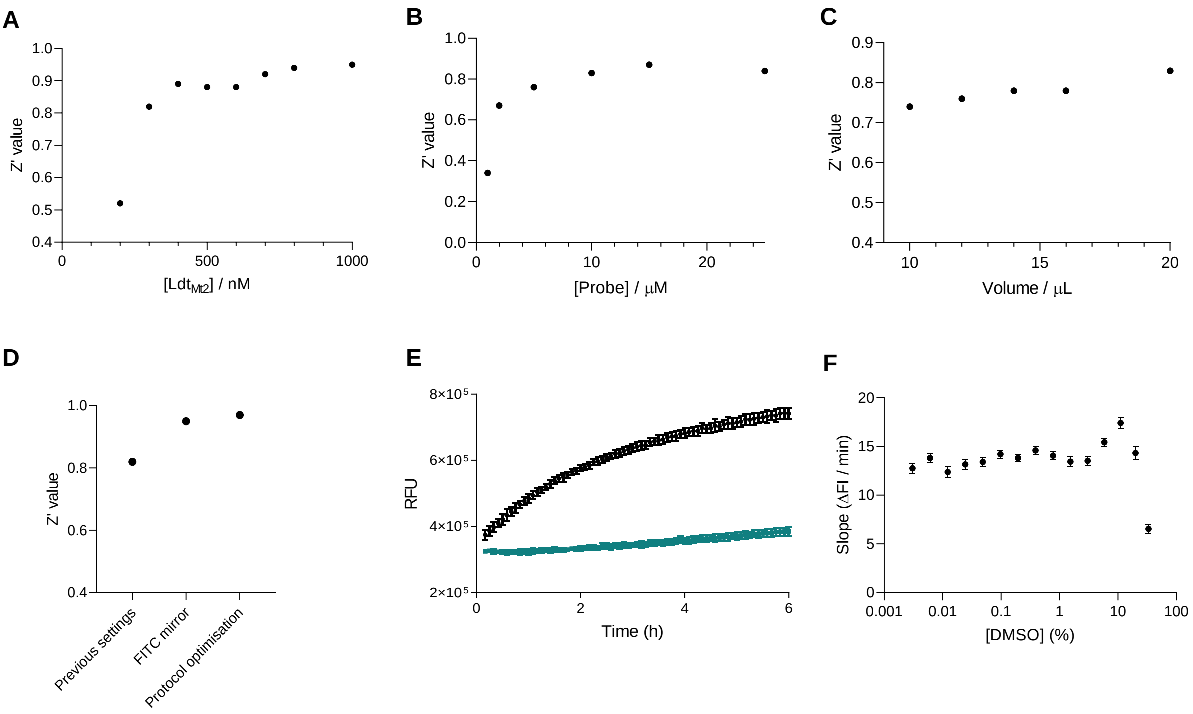

Figure S2. Optimisation of the HTS assay for Ldt_{Mt2}. (A) Z' values obtained with varying concentrations of Ldt_{Mt2} at a fixed probe **1** concentration (20 µM). The Z' values were ≥ 0.8 for Ldt_{Mt2} concentrations ≥ 300 nM. **(B)** Z' values obtained with varying concentrations of probe **1** at a fixed Ldt_{Mt2} concentration (300 nM). The optimal Z' value (0.87) was obtained with probe **1** at 15 µM. **(C)** Z' values obtained with varying reaction volumes, using fixed Ldt_{Mt2} (300 nM) and probe **1** (15 µM) concentrations. A lower reaction volume decreased the Z' value, but up to a volume of 10 µL, the Z' values are sufficiently high (≥ 0.40). **(D)** Optimisation of the plate reader settings improved the Z' values of the assay. The optimisation protocol included exchanging the general dual mirror to a FITC mirror increases the Z' value from 0.82 to 0.95. Optimising the plate reader protocol in terms of coordinates of corners, measurement height and gain further improved the Z' value to 0.97. **(E)** Time-course of the relative fluorescence units (RFU) measured on reaction of probe **1** (15 µM) with (300 nM, black) and without (teal) Ldt_{Mt2} at room temperature in (error bars; SD, n=32). The reaction between probe **1** and Ldt_{Mt2} was shown to reach completion after 5 hours, with the initial linear phase lasting 1 hour. **(F)** DMSO interference assay. Assays were performed at varied DMSO concentrations (error bars; SD, n=4). Up to a concentration of 3% (*v/v*) DMSO did not affect the assay. Assays were carried out with 300 nM Ldt_{Mt2} and 15 µM probe **1** at room temperature. Assays were carried out at room temperature in 50 mM sodium phosphate pH 7.5 with 0.01% (*v/v*) Triton X-100. Assays in (A) and (B) used reaction volumes of 25 µL. Assays in (D) - (F) used reaction volumes of 10 µL.

Table S1. Influence of detergents on Z' and signal to background ratio (S/B). The reaction between Ldt_{Mt2} (300 nM) and probe **1** (15 µM) was monitored at room temperature in 50 mM sodium phosphate pH 7.5 in the presence of the indicated detergents. The maximum tolerated concentrations of each detergent were determined in interference assays at varying detergent concentrations and were defined as the highest tested concentration at which no interference was observed.

**Detergent**	**Maximum tolerated concentration (*v/v*)**	**Z'**	**S/B**
Triton X-100	0.03%	0.77	2.5
Triton X-114	0.02%	0.77	2.3
Tween-20	0.007%	0.82	3.2
Tween-80	0.001%	0.66	2.2
MEGA-8	1.25%	0.77	3.3
CHAPS	0.245%	0.76	2.8
BRIJ-35	0.000054%	N.D.	N.D.
IPEGAL-630	0.00028%	N.D.	N.D.
DDM	0.00014%	N.D.	N.D.
BSA	<0.00064%	N.D.	N.D.
dBSA	<0.00064%	N.D.	N.D.

Table S2. Stability analysis of Ldt_{Mt2} and probe 1 stock solutions. The stabilities of stock solutions of Ldt_{Mt2} and probe **1** were assessed by comparing the Z' and signal to background ratios (S/B) of the assay using fresh and aged (7 h, on ice) stock solutions. The results show very similar Z' and S/B values. Assays were carried out with 300 nM Ldt_{Mt2} and 15 µM probe **1** at room temperature in 50 mM sodium phosphate pH 7.5 with 0.007% (*v/v*) Tween-20.

	**Z'**	**S/B**
Fresh stocks	0.97	14.5
7 hours old stocks	0.96	14.4

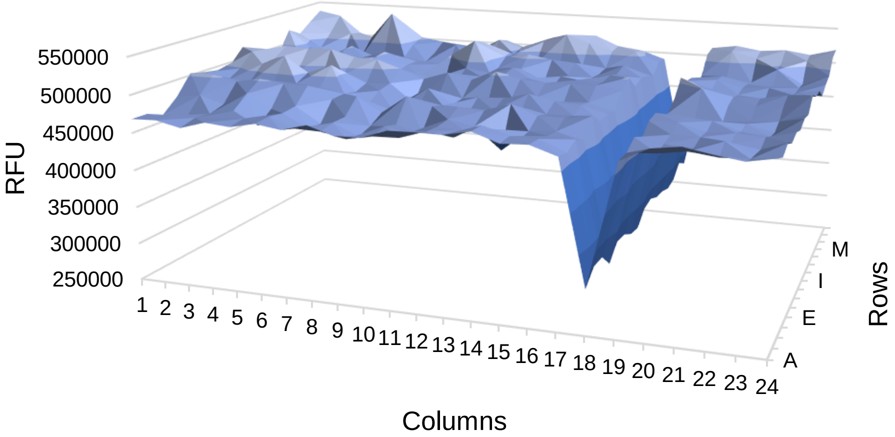

Figure S3. Analysis of a 384-well plate to test for variance in plate patterns. The full plate analysis using final assay conditions (300 nM Ldt_{Mt2} and 15 µM probe **1** at room temperature in 50 mM sodium phosphate pH 7.5 with 0.007% (*v/v*) Tween-20) shows no evidence for non-specific patterns across the assay plate, as measured 1 h after start of the reaction. Column 18 represents the no enzyme control.

Table S3. Dose-response analysis results for tool compounds. Compounds were incubated with Ldt_{Mt2} or Blac (10 min.), then assayed using the optimised assay conditions (see Experimental Details). Inhibition assays of Ldt_{Mt2} were carried out with 300 nM Ldt_{Mt2} and 15 µM probe **1** at room temperature in 50 mM sodium phosphate pH 7.5 with 0.007% (*v/v*) Tween-20. Inhibition assays of BlaC were carried out with 2.5 nM BlaC and 2.5 µM FC5 at room temperature in 100 mM sodium phosphate pH 7.5 with 0.01% (*v/v*) Triton X-100. pIC_{50}s were determined via non-linear regression in Graphpad Prism. (n=4, mean ± SD)

Compound	Structure	pIC_{50} Ldt_{Mt2} (mean ± SD)	pIC_{50} BlaC (mean ± SD)
Faropenem	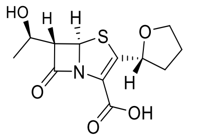	6.5 ± 0.04	6.35 ± 0.03
Sulopenem	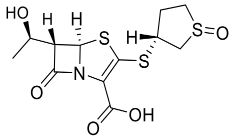	6.2 ± 0.03	6.60 ± 0.12
Panipenem	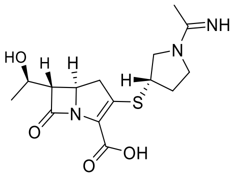	6.5 ± 0.05	6.78 ± 0.01
Ertapenem	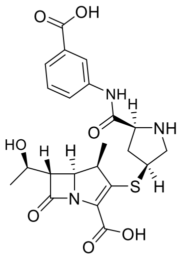	5.8 ± 0.08	6.75 ± 0.03
Doripenem	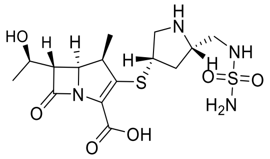	5.3 ± 0.16	7.38 ± 0.04
Meropenem	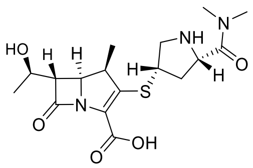	5.0 ± 0.11	6.85 ± 0.05
Biapenem	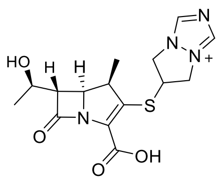	4.1 ± 0.03	5.51 ± 0.09
Flucloxacillin	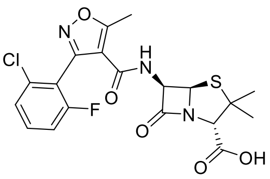	4.6 ± 0.09	6.87 ± 0.03

Compound	Structure	pIC$_{50}$ Ldt$_{Mt2}$ (mean ± SD)	pIC$_{50}$ BlaC (mean ± SD)
Oxacillin	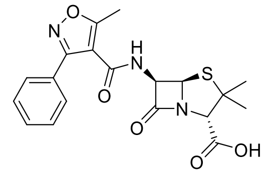	4.2 ± 0.03	6.80 ± 0.01
Ticarcillin	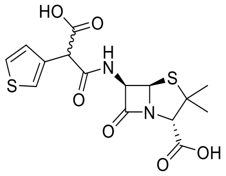	<4.0	4.93 ± 0.01
Carbenicillin	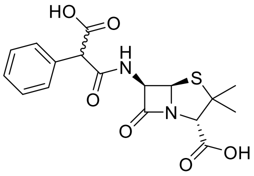	<4.0	<4.0
Sulbactam	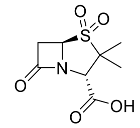	<4.0	5.12 ± 0.01
Zidebactam	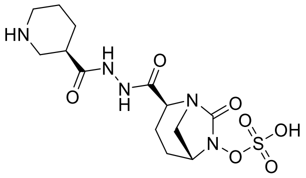	4.0 ± 0.02	4.15 ± 0.10
Relebactam	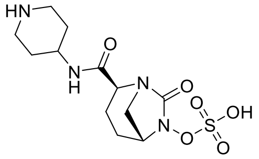	<4.0	4.48 ± 0.01
Ceftazidime	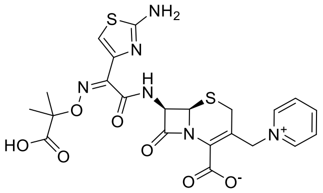	<4.0	4.40 ± 0.01
Cefminox	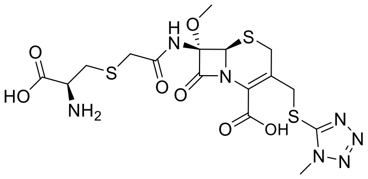	<4.0	4.50 ± 0.01
Cefotaxime	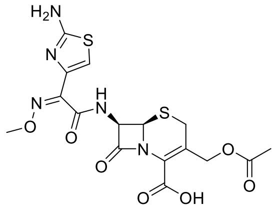	<4.0	<4.0
Ceftiofur	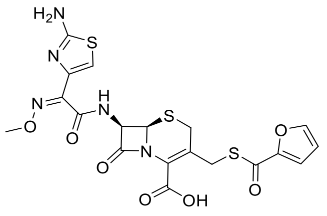	<4.0	<4.0

Compound	Structure	pIC_{50} Ldt_{Mt2} (mean ± SD)	pIC_{50} BlaC (mean ± SD)
Iodoacetamide	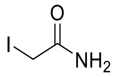	5.8 ± 0.03	<4.0
Maleimide	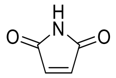	5.4 ± 0.04	<4.0
Ebselen	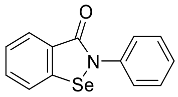	6.8 ± 0.03	5.82 ± 0.09
Benzeneseleninic acid	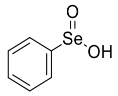	5.0 ± 0.06	<4.0
Thiram	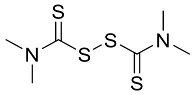	6.8 ± 0.03	<4.0
PX-12	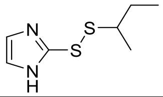	5.9 ± 0.01	<4.0

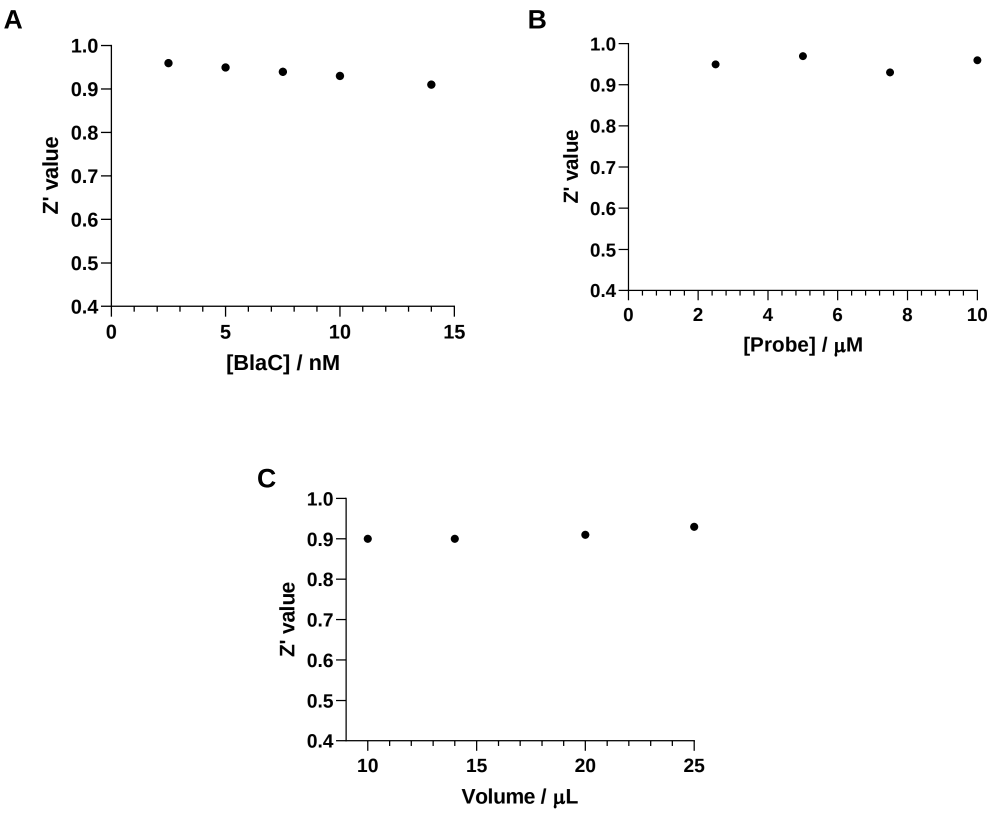

Figure S4. Optimisation of the HTS assay for BlaC. (A) Z' values obtained with varying concentrations of BlaC using a fixed concentration of probe FC5[2] (10 μM). **(B)** Z' values obtained with varying concentrations of FC5 using a fixed BlaC concentration (2.5 nM). A Z' value of 0.95 was obtained at a FC5 concentration of 2.5 μM. **(C)** Z' values obtained with varying reaction volumes using fixed BlaC and FC5 concentrations (2.5 nM and 2.5 μM, respectively). Assays in (A) and (B) used reaction volumes of 25 μL. Assays were performed at room temperature in 100 mM sodium phosphate pH 7.5 with 0.01% (*v/v*) Triton X-100.

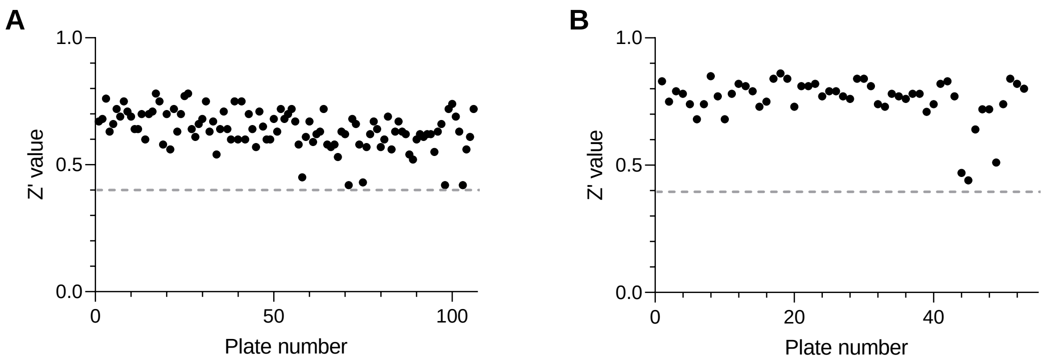

Figure S5. Z' values of the plates throughout the HTS campaign. (A) Z' values for the Ldt_{Mt2} HTS assay. **(B)** Z' values for the HTS assay for the BlaC HTS assay. The cut-off value for plate acceptance is indicated at Z' > 0.4.

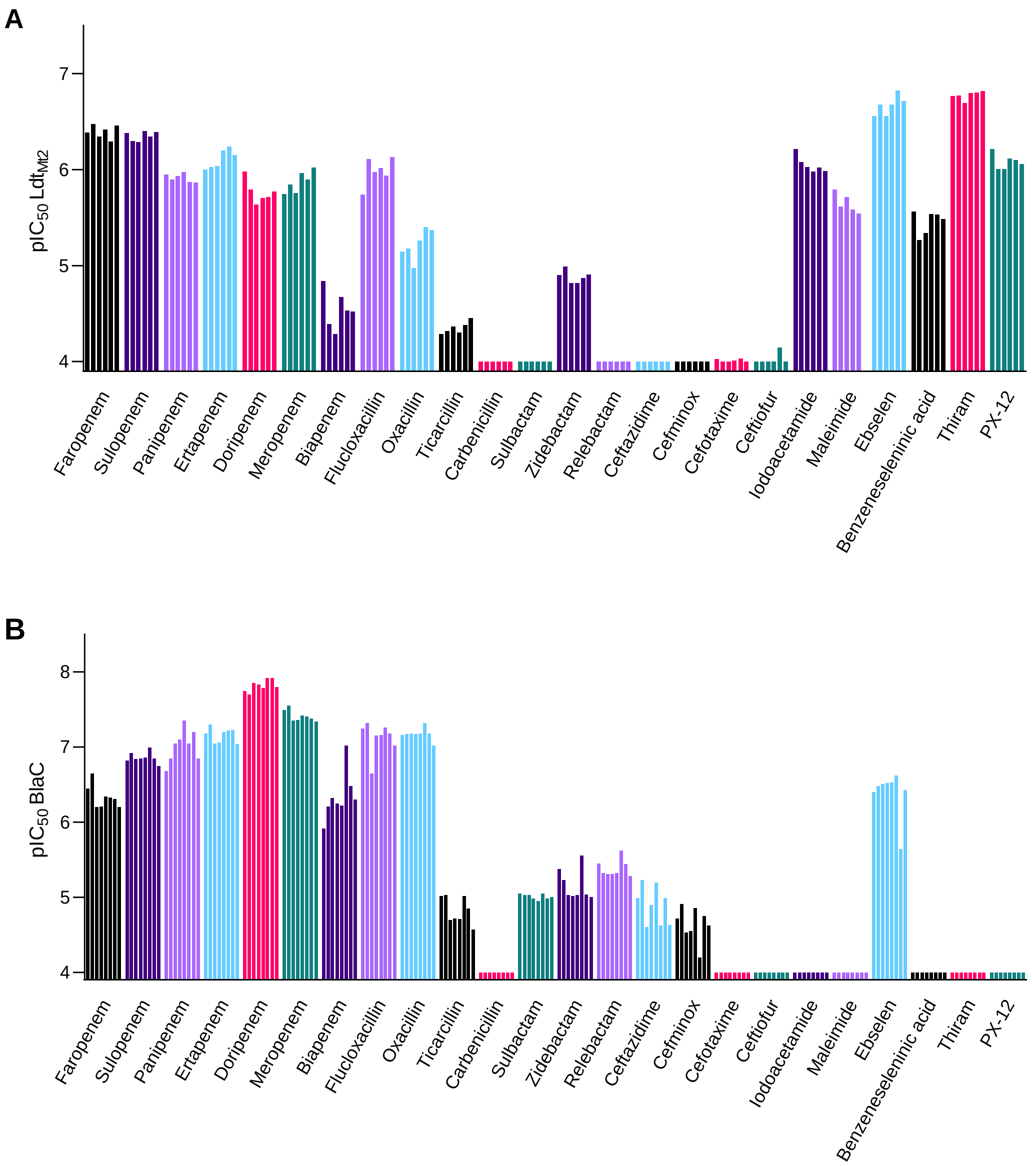

Figure S6. Dose response assay results of tool compounds as HTS validation. pIC_{50} values for Ldt_{Mt2} inhibition **(A)** and BlaC inhibition **(B)** were measured on different days, and show good reproducibility for the tested compounds. Inhibition assays of Ldt_{Mt2} were carried out with 300 nM Ldt_{Mt2} and 15 µM probe **1** with 30 minutes inhibitor pre-incubation at room temperature in 50 mM sodium phosphate pH 7.5 with 0.007% (*v/v*) Tween-20. Inhibition assays of BlaC were carried out with 2.5 nM BlaC and 2.5 µM FC5 with 30 minutes inhibitor pre-incubation at room temperature in 100 mM sodium phosphate pH 7.5 with 0.01% (*v/v*) Triton X-100. Average pIC_{50} values and compound structures are given in Table S4.

Table S4. Validation of the HTS assay using tool compounds. pIC_{50} values for Ldt_{Mt2} and BlaC inhibition, as obtained from independent repeats of technical duplicates during the HTS validation. Errors given are SD of the independent repeats (Ldt_{Mt2} assay n=6, BlaC assay n=8). Inhibition assays of Ldt_{Mt2} were carried out with 300 nM Ldt_{Mt2} and 15 µM probe **1** with 30 minutes inhibitor pre-incubation at room temperature in 50 mM sodium phosphate pH 7.5 with 0.007% (*v/v*) Tween-20. Inhibition assays of BlaC were carried out with 2.5 nM BlaC and 2.5 µM FC5 with 30 minutes inhibitor pre-incubation at room temperature in 100 mM sodium phosphate pH 7.5 with 0.01% (*v/v*) Triton X-100.

**Compound**	**Structure**	**pIC_{50} Ldt_{Mt2} (mean ± SD)**	**pIC_{50} BlaC (mean ± SD)**
Faropenem	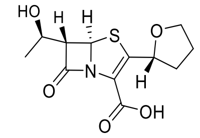	6.39 ± 0.03	6.33 ± 0.06
Sulopenem	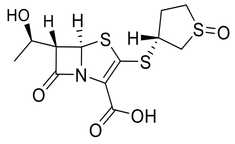	6.35 ± 0.02	6.86 ± 0.02
Panipenem	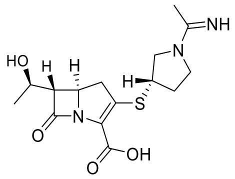	5.91 ± 0.02	7.03 ± 0.06
Ertapenem	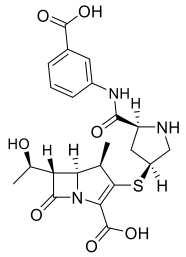	6.11 ± 0.04	7.17 ± 0.03
Doripenem	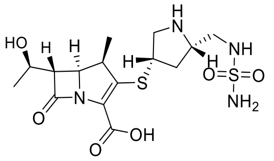	5.77 ± 0.04	7.85 ± 0.02
Meropenem	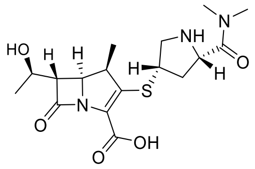	5.87 ± 0.04	7.39 ± 0.03
Biapenem	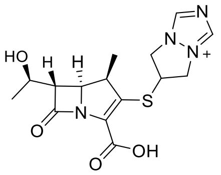	5.54 ± 0.07	6.32 ± 0.11
Flucloxacillin	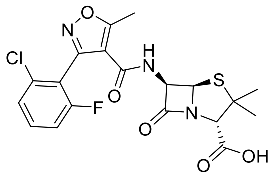	5.98 ± 0.06	7.11 ± 0.07

Compound	Structure	pIC_{50} Ldt_{Mt2} (mean ± SD)	pIC_{50} BlaC (mean ± SD)
Oxacillin	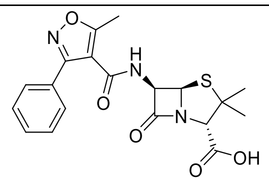	5.22 ± 0.06	7.17 ± 0.03
Ticarcillin	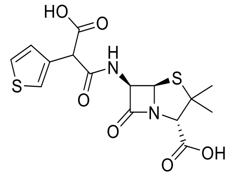	4.35 ± 0.02	4.84 ± 0.06
Carbenicillin	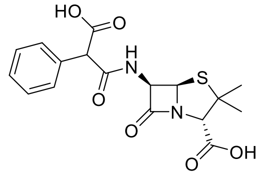	<4.0	<4.0
Sulbactam	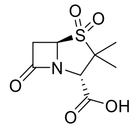	<4.0	5.02 ± 0.03
Zidebactam	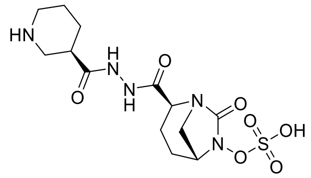	4.88 ± 0.02	5.14 ± 0.10
Relebactam	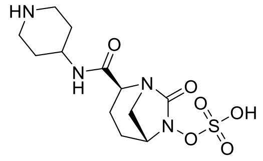	<4.0	5.40 ± 0.04
Ceftazidime	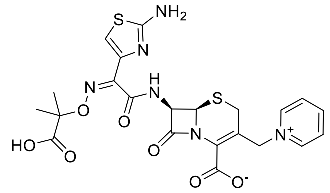	<4.0	4.92 ± 0.07
Cefminox	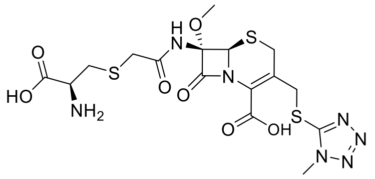	<4.0	4.66 ± 0.08
Cefotaxime	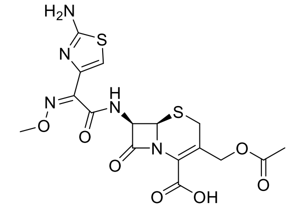	<4.0	<4.0
Ceftiofur	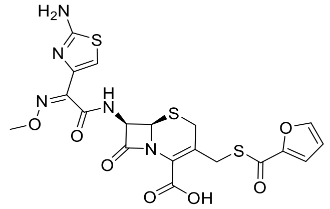	<4.0	<4.0

Compound	Structure	pIC_{50} Ldt_{Mt2} (mean ± SD)	pIC_{50} BlaC (mean ± SD)
Iodoacetamide	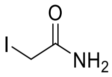	6.05 ± 0.03	<4.0
Maleimide	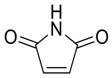	5.65 ± 0.04	<4.0
Ebselen	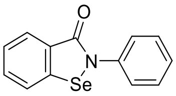	6.67 ± 0.04	6.52 ± 0.04
Benzeneseleninic acid	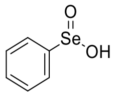	5.45 ± 0.05	<4.0
Thiram	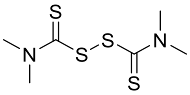	6.77 ± 0.02	<4.0
PX-12	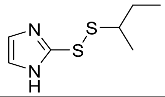	6.09 ± 0.03	<4.0

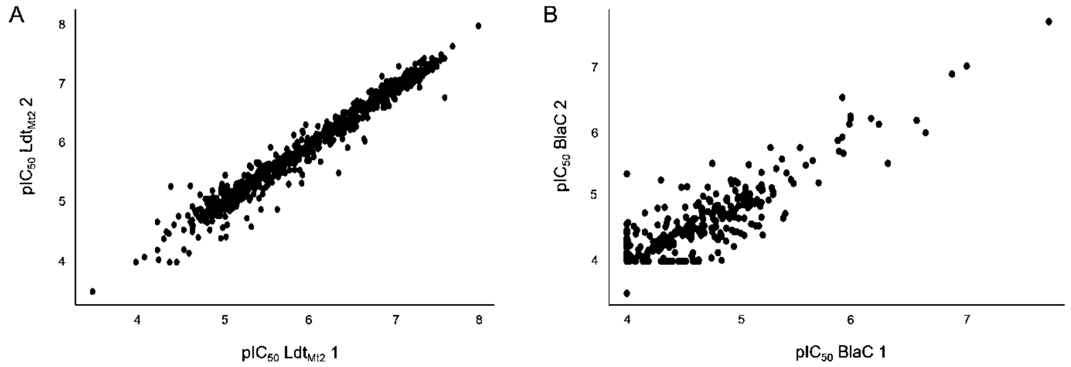

Figure S7. Correlation between pIC_{50} values from two independent repeats in the HTS. (A) for Ldt_{Mt2} and **(B)** for BlaC of the two independent repeats using the standard assay conditions (Figure S2E, Figure S4C).

Table S5. Eight classes of compounds identified from the Ldt_{Mt2} HTS. MW: Molecular weight. LE: Ligand efficiency. T_m: Melting point (°C) of Ldt_{Mt2} after pre-incubation with the compound as measured using SYPRO Orange (6 x concentrated, according to the manufacturer's (Invitrogen) protocol) in 50 mM Tris pH 7.5. ΔT_m: Difference in melting point (T_m) of Ldt_{Mt2} after pre-incubation with the compound relative to unreacted Ldt_{Mt2} (43.8 °C). k_{chem}: apparent intrinsic thiol reactivity as measured with *L*-glutathione and probe **2** (Figure S20).

Class		Compound	MW (Da)	pIC_{50}		LE	T_m (ΔT_m)	k_{inact}/K_I (M^{-1} s^{-1})	k_{chem} (M^{-1} s^{-1})
				Ldt_{Mt2}	BlaC				
**α-chloro ketone**	**1**	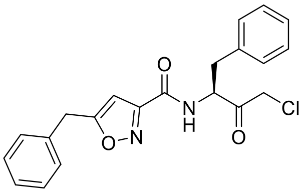	382.85	7.34 ± 0.045	<4.0	0.41	N.D.	1152 ± 87	0.11 ± 0.02
	**2**	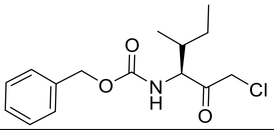	297.78	7.11 ± 0.010	<4.0	0.56	43.7 ± 0.09 (-0.1)	68.4 ± 3.5	0.15 ± 0.02
**Maleimides**	**3**	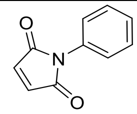	173.17	7.15 ± 0.030	5.03 ± 0.045	0.86	45.0 ± 0.16 (+1.2)	3991 ± 356	129.9 ± 9.9
	**4**	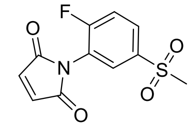	269.25	7.06 ± 0.035	4.22 ± 0.060	0.62	45.6 ± 0.28 (+1.8)	36.5 ± 7.9	34.2 ± 1.0
	**5**	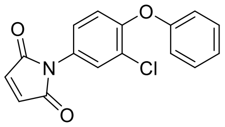	299.71	7.40 ± 0.010	4.71 ± 0.055	0.53	43.6 ± 0.16 (-0.2)	116 ± 6.2	91.8 ± 1.8
	**6**	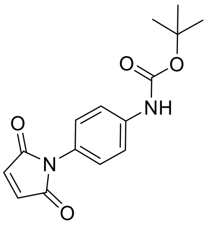	288.30	7.24 ± 0.005	5.19 ± 0.005	0.52	45.5 ± 0.09 (+1.7)	127 ± 7.7	61.6 ± 0.78
	**7**	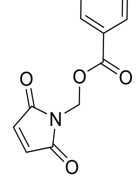	231.21	7.02 ± 0.000	4.18 ± 0.175	0.66	43.1 ± 0.09 (-0.7)	156 ± 5.1	91.7 ± 4.5
**Acrylamides**	**8**	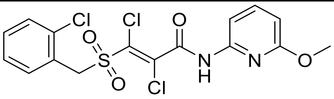	435.71	7.16 ± 0.035	<4.0	0.43	44.5 ± 1.23 (+0.7)	91.6 ± 2.0	1.53 ± 0.15
	**9**	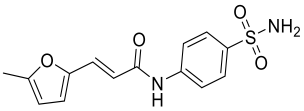	306.34	6.68 ± 0.025	4.58 ± 0.010	0.53	43.1 ± 0.09 (-0.7)	90.7 ± 4.7	1.76 ± 0.20
	**10**	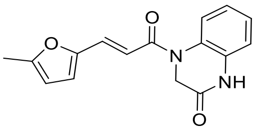	282.30	6.37 ± 0.005	4.29 ± 0.290	0.54	44.5 ± 0.19 (-0.7)	16.0 ± 0.75	0.79 ± 0.02

Class		Compound	MW (Da)	pIC_{50} Ldt$_{Mt2}$	pIC_{50} BlaC	LE	T_m (ΔT_m)	k_{inact}/K_I (M^{-1} s^{-1})	k_{chem} (M^{-1} s^{-1})
**Acrylamides (continued)**	**11**	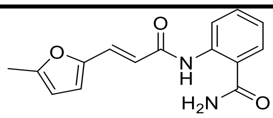	270.29	6.24 ± 0.030	<4.0	0.56	44.0 ± 0.16 (+0.2)	172 ± 6.0	0.27 ± 0.05
	**12**	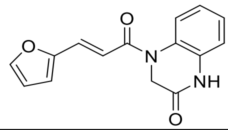	268.27	6.13 ± 0.115	<4.0	0.56	44.1 ± 0.09 (+0.3)	N.D.	0.55 ± 0.11
**Fumaryl-amides**	**13**	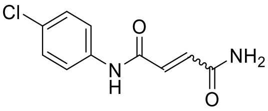	224.65	6.38 ± 0.080	<4.0	0.75	44.8 ± 0.00 (+1.0)	6.48 ± 0.11	6.1 ± 0.52
	**14**	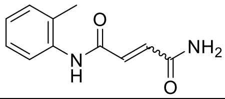	204.23	6.11 ± 0.020	<4.0	0.75	44.9 ± 0.09 (+1.1)	1.20 ± 0.15	13.0 ± 1.0
**Ebsulfur analogue**	**15**	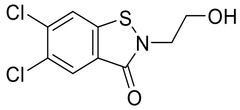	264.13	7.99 ± 0.040	6.37 ± 0.175	0.74	43.5 ± 0.09 (-0.3)	N.D.	N.D.
**Isatins**	**16**	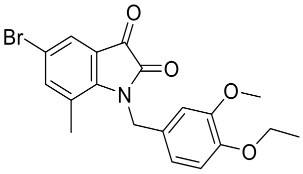	404.26	6.75 ± 0.015	4.44 ± 0.015	0.45	46.8 ± 0.00 (+3.0)	N.D.	<0.08
	**17**	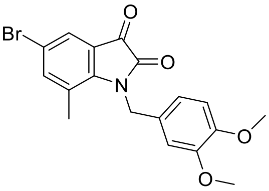	390.23	6.24 ± 0.140	<4.0	0.47	46.8 ± 0.00 (+3.0)	N.D.	<0.08
	**18**	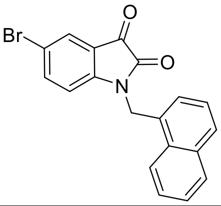	366.21	6.52 ± 0.005	<4.0	0.49	46.2 ± 0.00 (+2.4)	N.D.	<0.08
**β-Lactams**	**19**	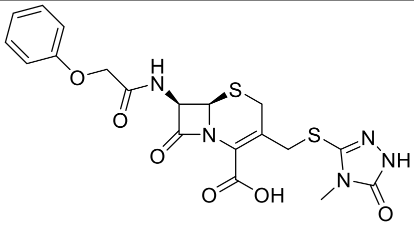	477.52	5.62 ± 0.100	<4.0	0.35	42.9 ± 0.09 (-0.9)	N.D.	N.D.
	**20**	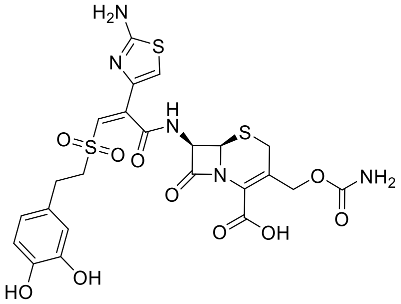	625.66	5.55 ± 0.045	7.72 ± 0.015	0.28	42.7 ± 0.25 (-1.1)	N.D.	N.D.

Class		Compound	MW (Da)	pIC_{50} Ldt$_{Mt2}$	pIC_{50} BlaC	LE	T_m (ΔT_m)	k_{inact}/K_I ($M^{-1} s^{-1}$)	k_{chem} ($M^{-1} s^{-1}$)
**β-Lactams (continued)**	**21**	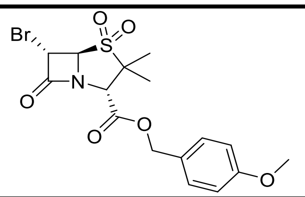	432.29	6.40 ± 0.035	4.58 ± 0.195	0.45	43.1 ± 0.09 (-0.7)	53.2 ± 4.7	2.03 ± 0.76
**Nitriles**	**22**	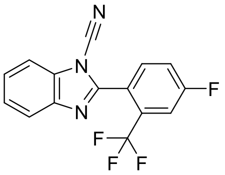	305.23	7.53 ± 0.010	<4.0	0.51	42.5 ± 0.09 (-1.3)	55.1 ± 3.0	20.3 ± 0.9
	**23**	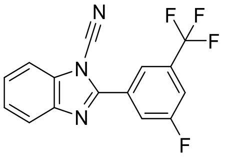	305.23	7.33 ± 0.065	<4.0	0.51	42.5 ± 0.09 (-1.3)	265 ± 17	15.0 ± 0.41
	**24**	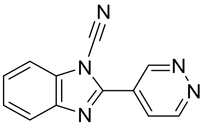	221.22	7.34 ± 0.030	<4.0	0.66	42.0 ± 0.00 (-1.8)	158 ± 5.7	27.1 ± 2.3
	**25**	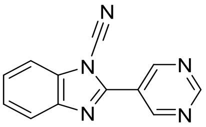	221.22	7.34 ± 0.050	<4.0	0.66	42.4 ± 0.00 (-1.8)	77.5 ± 6.0	28.7 ± 1.9
	**26**	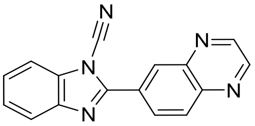	271.28	7.45 ± 0.015	4.83 ± 0.110	0.53	41.7 ± 0.09 (-2.1)	65.0 ± 4.4	11.4 ± 0.62
	**27**	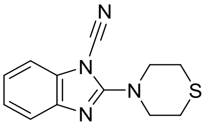	244.32	7.02 ± 0.025	<4.0	0.66	42.5 ± 0.09 (-1.3)	14.0 ± 0.73	6.50 ± 0.25
	**28**	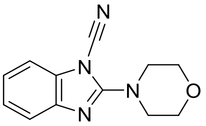	228.25	6.39 ± 0.120	<4.0	0.66	42.3 ± 0.09 (-1.5)	7.41 ± 0.40	2.12 ± 0.15
	**29**	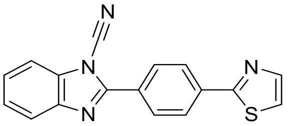	302.36	7.28 ± 0.010	6.18 ± 0.040	0.51	42.9 ± 0.19 (-0.9)	1164 ± 105	42.1 ± 0.82
	**30**	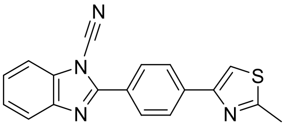	316.39	7.25 ± 0.065	6.11 ± 0.150	0.49	42.2 ± 0.00 (-1.6)	236 ± 9.5	N.D.
	**31**	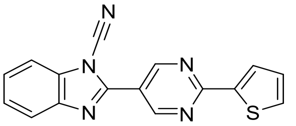	303.35	7.32 ± 0.055	4.95 ± 0.070	0.51	42.1 ± 0.10 (-1.7)	609 ± 11	21.0 ± 1.4
	**32**	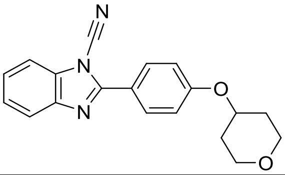	319.36	7.31 ± 0.045	4.73 ± 0.080	0.47	42.3 ± 0.09 (-1.5)	86.3 ± 2.6	3.58 ± 0.19

Class		Compound	MW (Da)	pIC_{50}		LE	T_m (ΔT_m)	k_{inact}/K_I (M^{-1} s^{-1})	k_{chem} (M^{-1} s^{-1})
				Ldt_{Mt2}	BlaC				
Nitriles (continued)	**33**	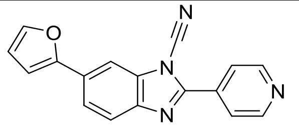	286.29	6.95 ± 0.045	6.09 ± 0.125	0.51	43.3 ± 0.09 (-0.5)	71.8 ± 1.1	12.9 ± 0.67
	**34**	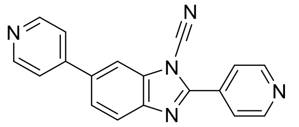	297.32	7.20 ± 0.080	<4.0	0.49	42.1 ± 0.09 (-1.7)	68.4 ± 2.7	31.0 ± 1.6
	**35**	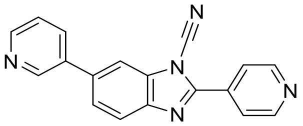	297.32	7.01 ± 0.015	5.91 ± 0.385	0.49	42.9 ± 0.34 (-0.9)	8.88 ± 0.39	16.8 ± 0.61
	**36**	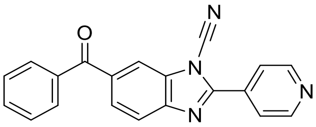	324.34	7.11 ± 0.050	<4.0	0.45	40.8 ± 0.59 (-3.0)	51.0 ± 3.4	61.6 ± 2.7
	**37**	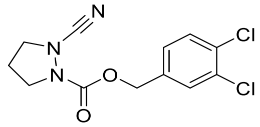	300.14	6.43 ± 0.025	4.47 ± 0.075	0.59	42.3 ± 0.09 (-1.5)	8.44 ± 1.2	0.30 ± 0.03
	**38**	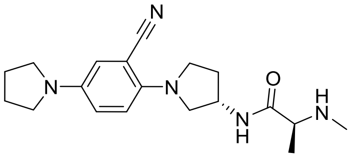	341.46	6.15 ± 0.010	<4.0	0.45	42.8 ± 0.00 (-1.0)	N.D.	N.D.
	**39**	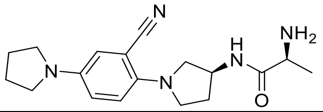	327.43	6.29 ± 0.065	<4.0	0.47	43.1 ± 0.10 (-0.7)	N.D.	N.D.

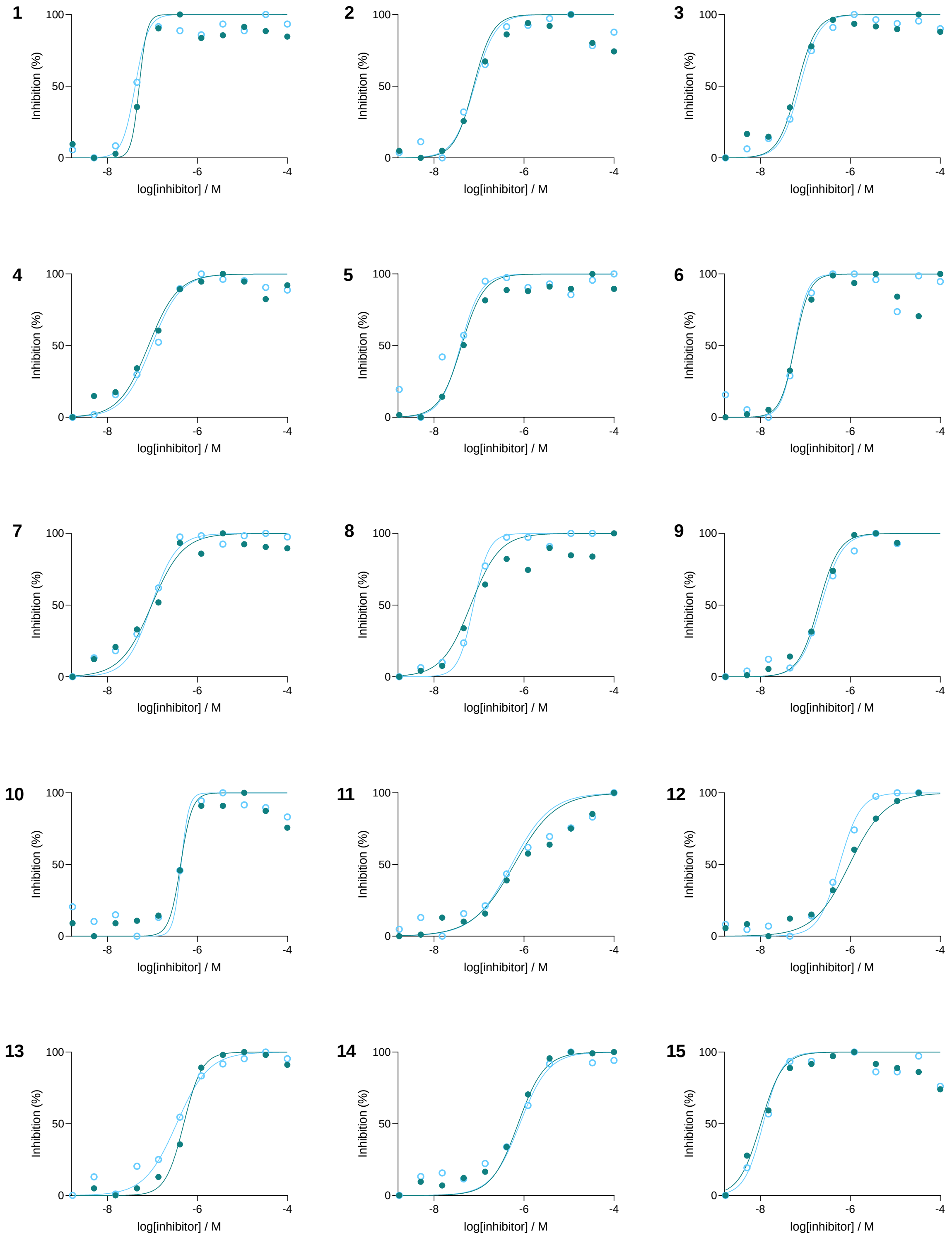

Figure S8. Dose-response curves for compounds 1-39 with Ldt_{Mt2} (figure continues).

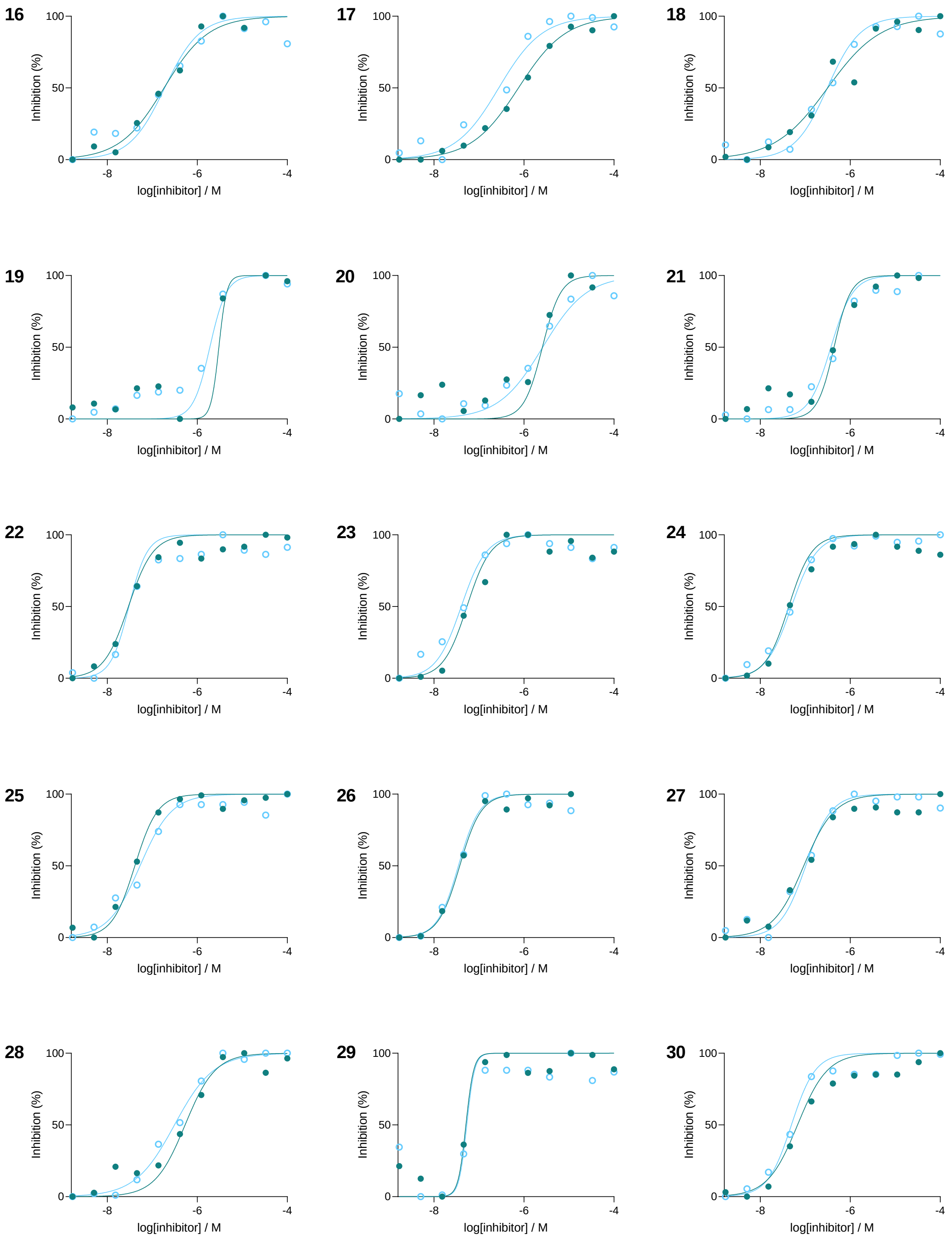

Figure S8. Dose-response curves for compounds 1-39 with Ldt$_{Mt2}$ (figure continues).

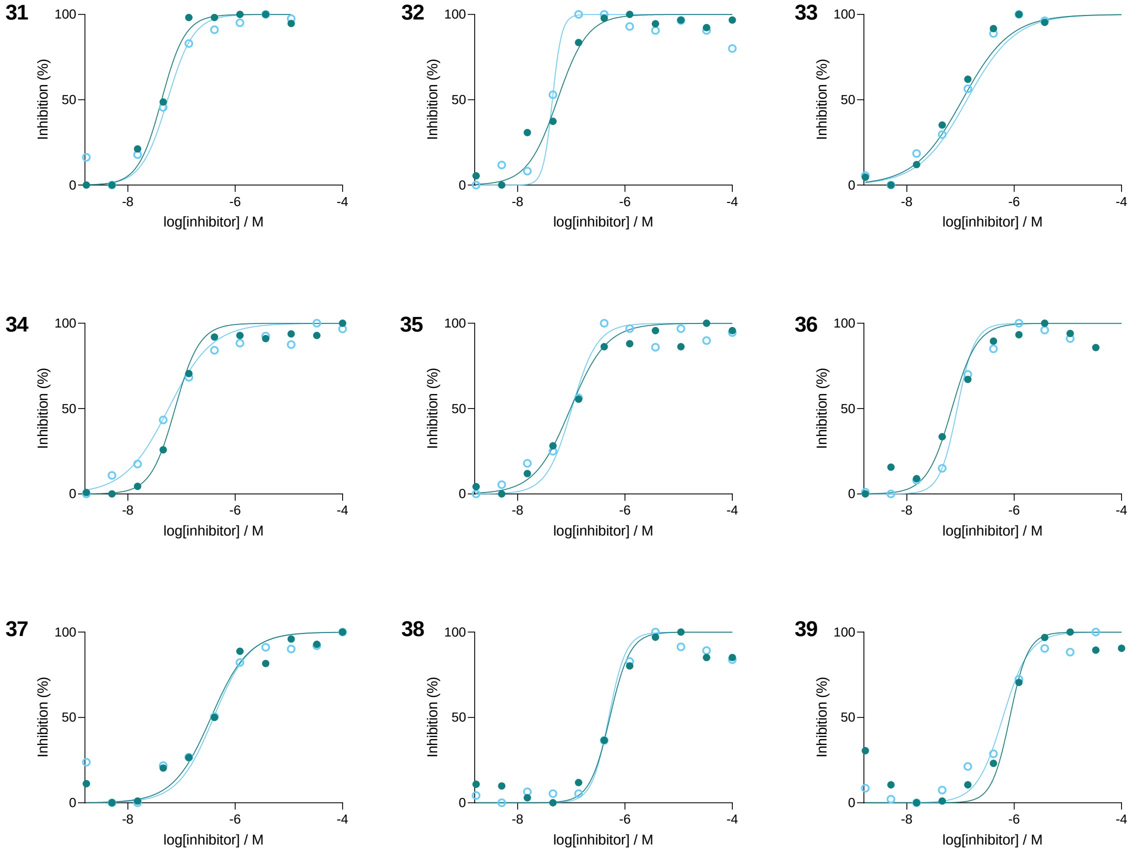

Figure S8. Dose-response curves for compounds 1-39 with $\mathbf{Ldt_{Mt2}}$. Inhibition assays of Ldt_{Mt2} were carried out with 300 nM Ldt_{Mt2} and 15 µM probe **1** with a 30 minute inhibitor pre-incubation at room temperature in 50 mM sodium phosphate pH 7.5 with 0.007% (*v/v*) Tween-20. Two independent repeats are shown in teal and cyan. Average pIC_{50} values and compound structures are given in Table S5.

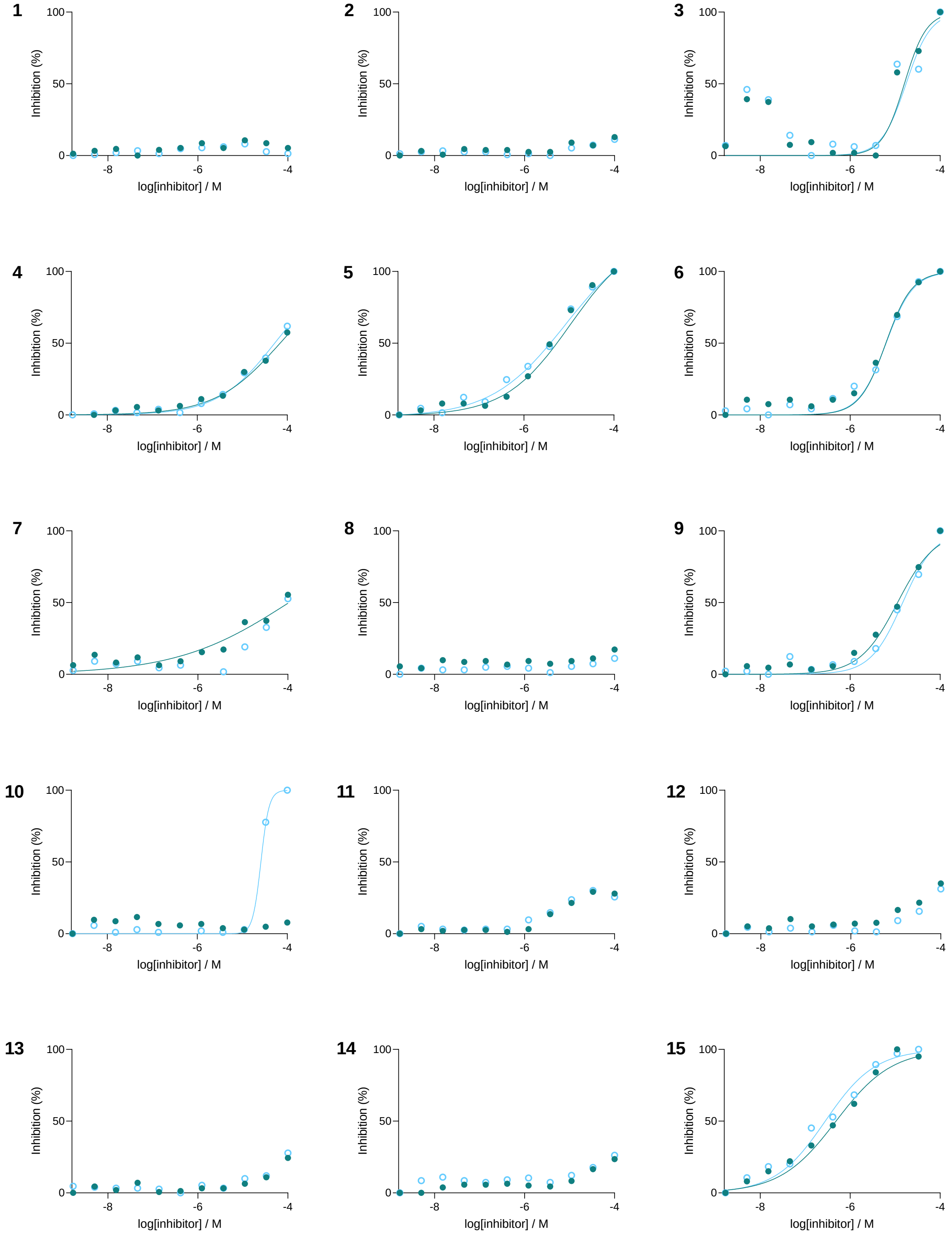

Figure S9. Dose-response curves for compounds 1-39 with BlaC (figure continues).

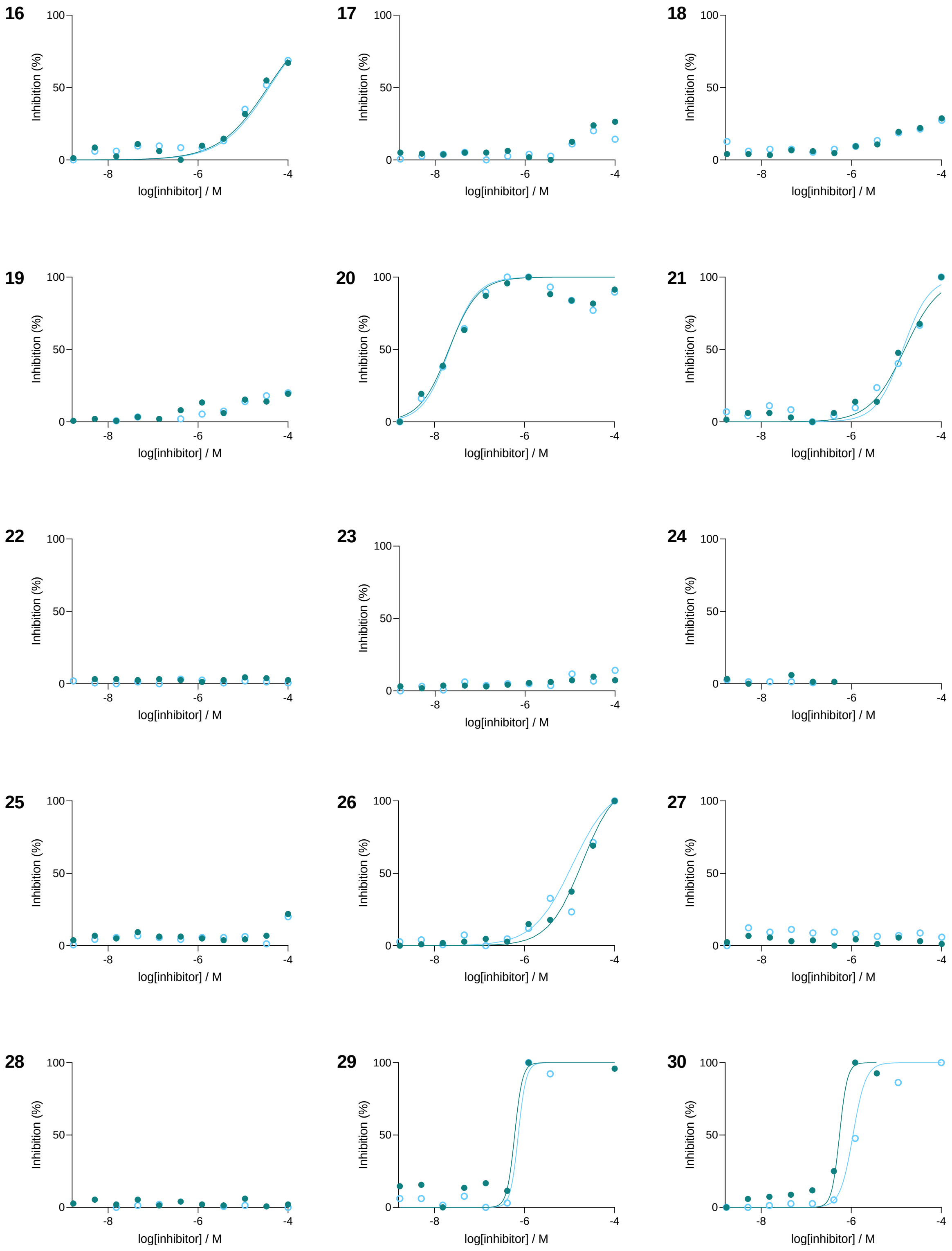

Figure S9. Dose-response curves for compounds 1-39 with BlaC (figure continues).

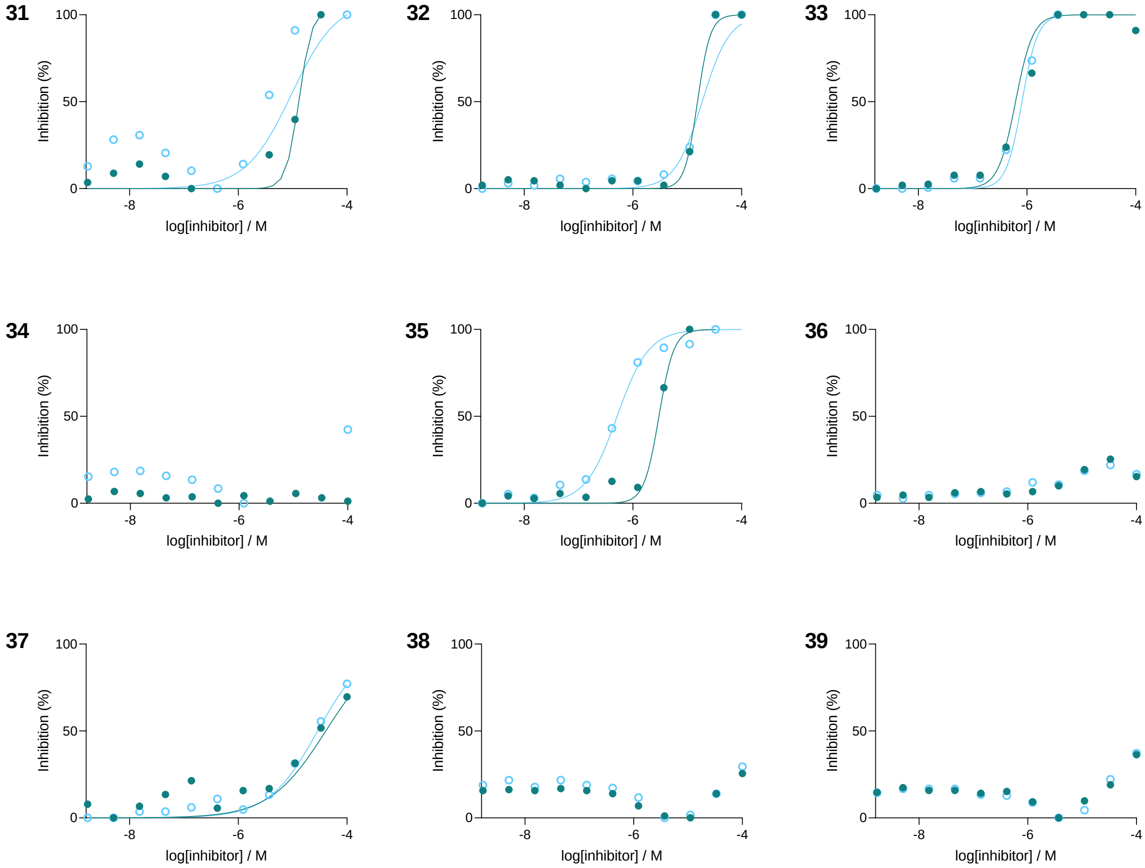

Figure S9. Dose-response curves for compounds 1-39 with BlaC. Inhibition assays of BlaC were carried out with 2.5 nM BlaC and 2.5 µM FC5 with a 30 minute inhibitor pre-incubation at room temperature in 100 mM sodium phosphate pH 7.5 with 0.01% (*v/v*) Triton X-100. Two independent repeats are shown in teal and cyan. Average pIC_{50} values and compound structures are given in Table S5.

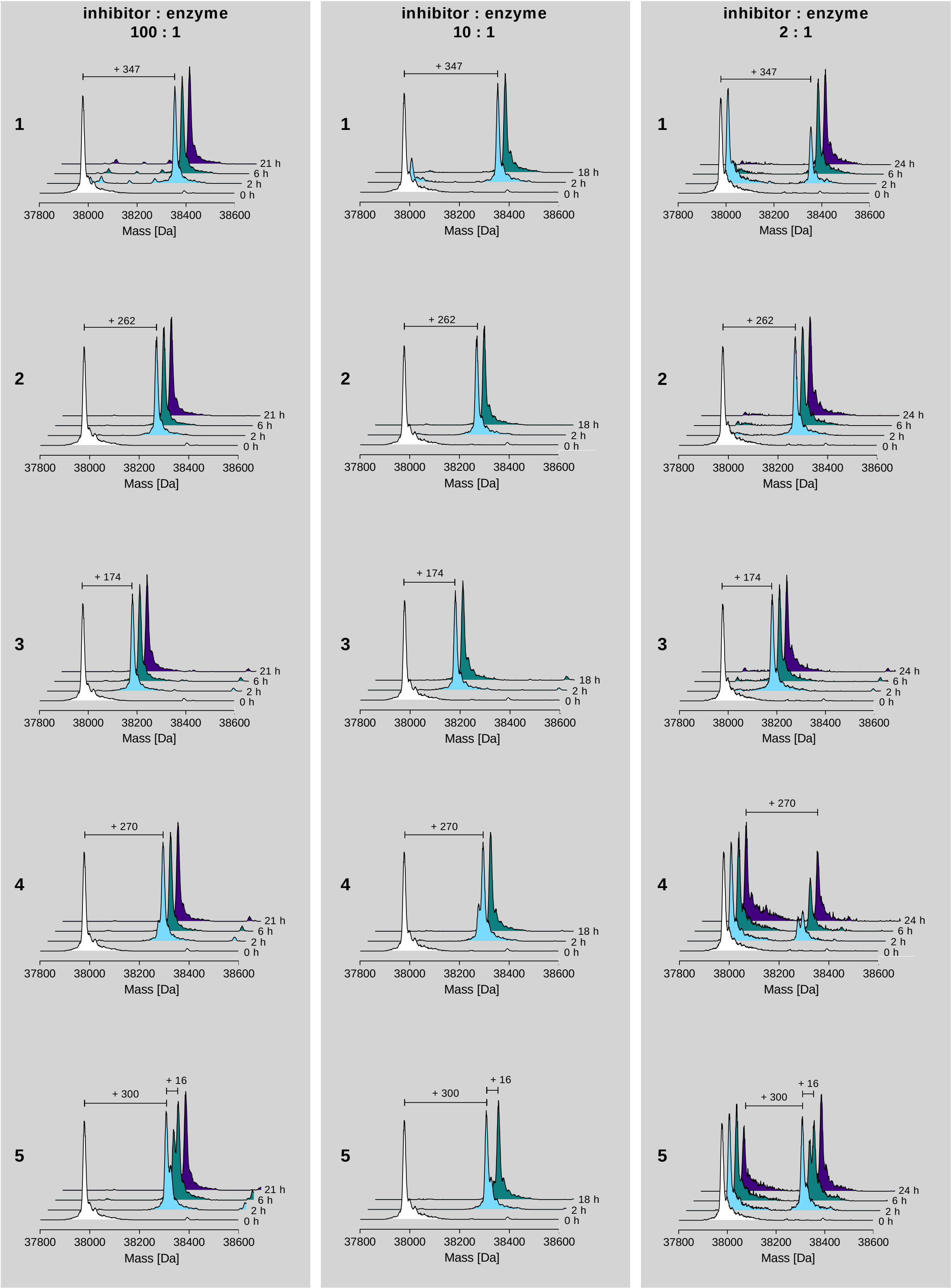

Figure S10. Protein observed SPE-MS analysis for the reaction of Ldt$_{Mt2}$ with inhibitors 1-21 (figure continues).

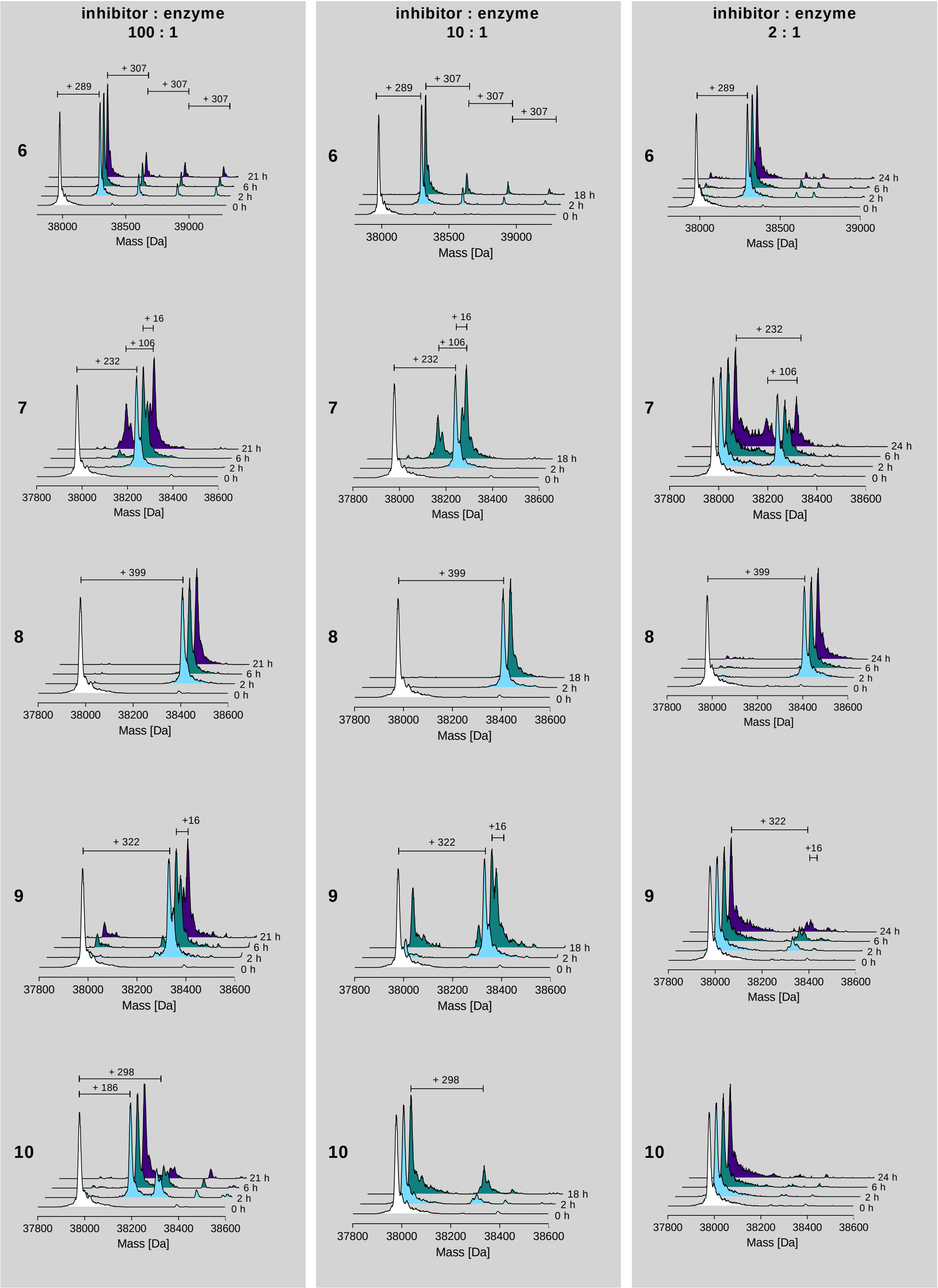

Figure S10. Protein observed SPE-MS analysis for the reaction of Ldt_{Mt2} with inhibitors 1-21 (figure continues).

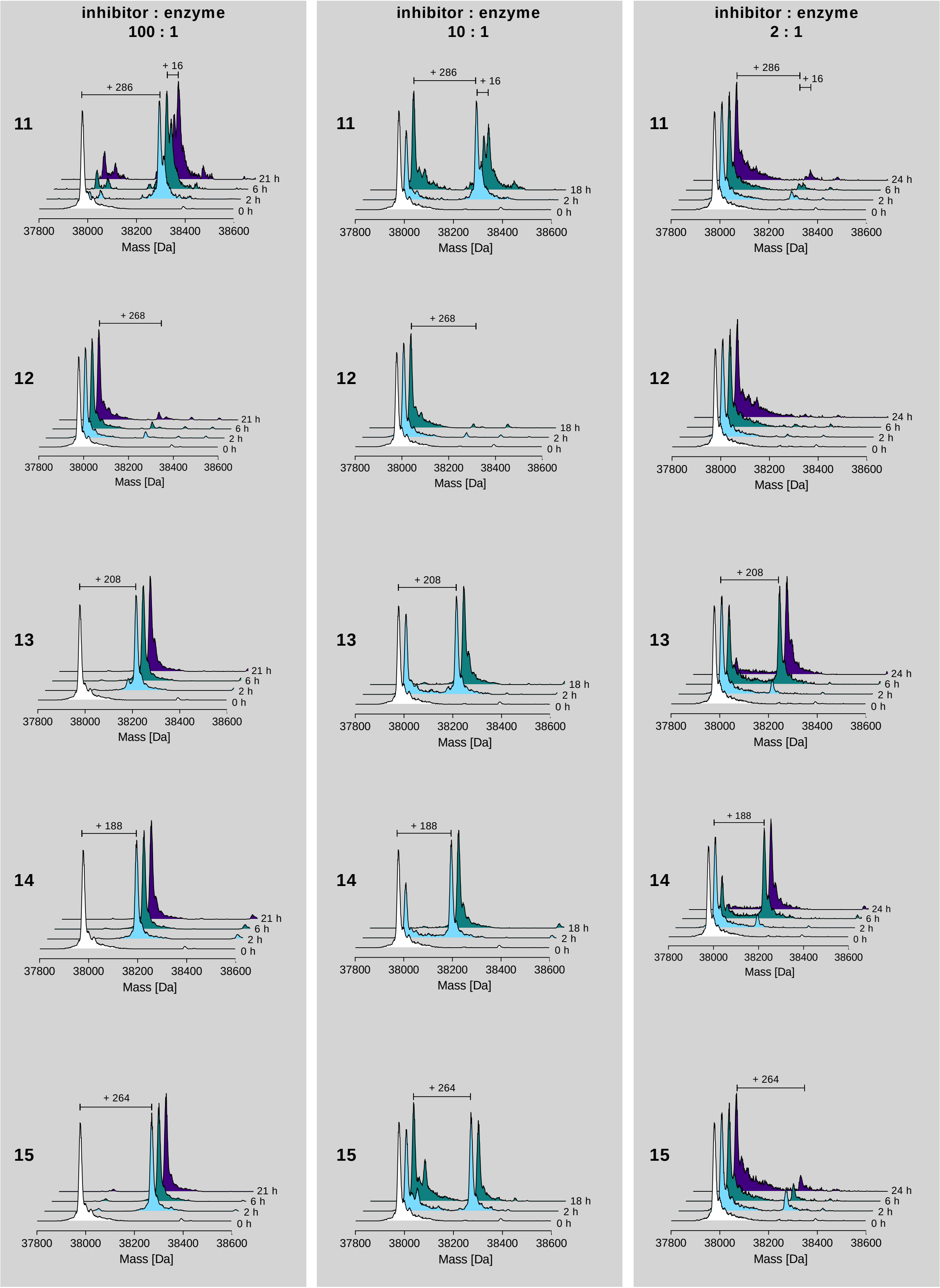

Figure S10. Protein observed SPE-MS analysis for the reaction of Ldt$_{Mt2}$ with inhibitors 1-21 (figure continues).

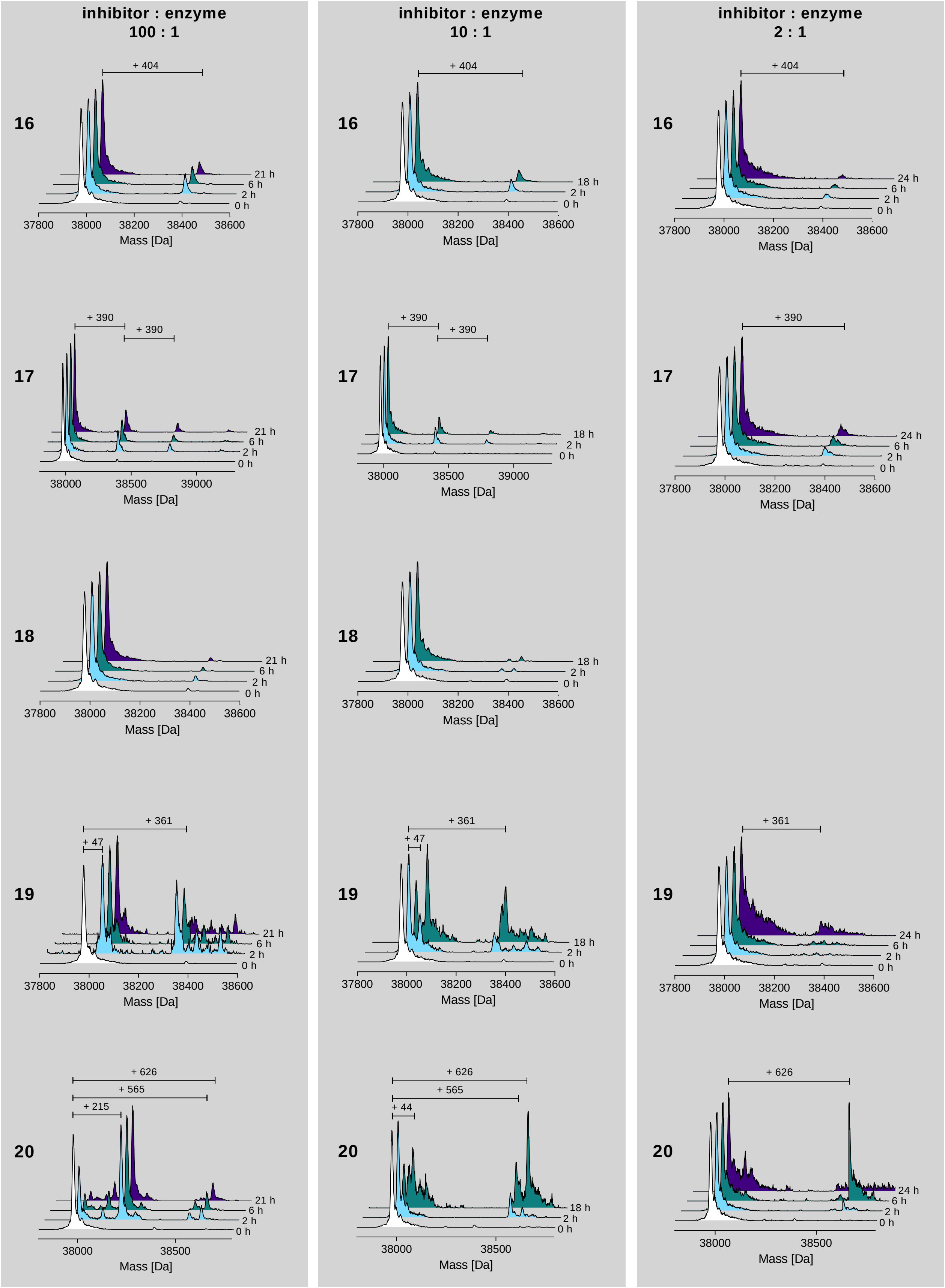

Figure S10. Protein observed SPE-MS analysis for the reaction of Ldt_{Mt2} with inhibitors 1-21 (figure continues).

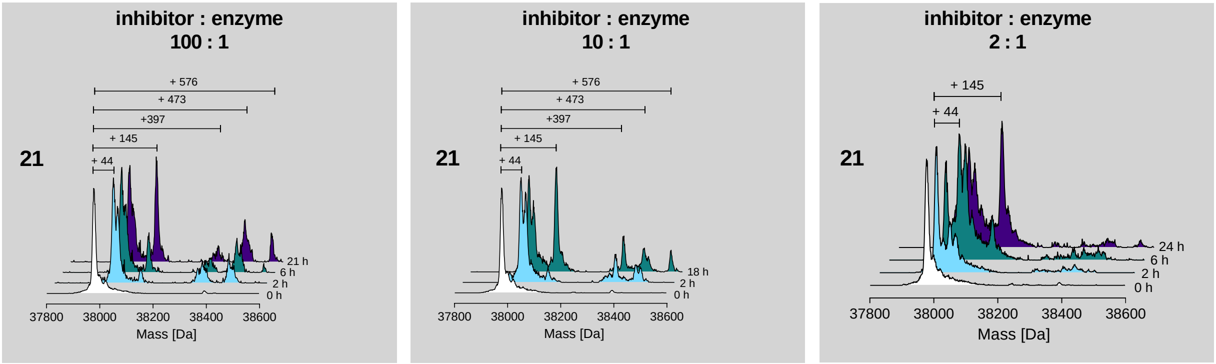

Figure S10. Protein observed SPE-MS analysis for the reaction of Ldt$_{Mt2}$ with inhibitors 1-21. 1 µM Ldt$_{Mt2}$ was incubated with the inhibitors at the specified ratio at room temperature in 50 mM Tris, pH 7.5. Samples were analysed after the indicated times. Compound structures are given in Table S5. Deconvoluted spectra, obtained using the maximum entropy algorithm in the MassHunter Workstation Qualitative Analysis B.07.00 program (Agilent), are shown.

Table S6. Calculated (based on anticipated binding mode) and observed masses (Da) and mass shifts (Da) for protein observed SPE-MS experiments with Ldt_{Mt2} and the indicated inhibitors. Mass shifts are relative to unmodified Ldt_{Mt2}. * Calculated mass for reaction at the inhibitor terminal amide group. ** Calculated mass for reaction at the inhibitor acrylamide group. Compound structures are given in Table S5. Deconvoluted SPE-MS spectra are shown in Figure S10.

Compound	Calculated mass (Da)	Observed mass (Da)
**1**	38334 (+347)	38334 (+347)
**2**	38249 (+262)	38249 (+262)
**3**	38161 (+174)	38161 (+174)
**4**	38257 (+270)	38257 (+270)
**5**	38287 (+300)	38287 (+300), 38303 (+316)
**6**	38276 (+289)	38276 (+289), 38582 (+595), 38889 (+902), 39196 (+1209)
**7**	38219 (+232)	38113 (+126), 38219 (+232), 38235 (+248)
**8**	38386 (+399)	38386 (+399)
**9**	38293 (+306)	38309 (+322), 38325 (+338)
**10**	38269 (+282)	38173 (+186), 38285 (+298)
**11**	38257 (+270)	38273 (+286), 38289 (+302)
**12**	38255 (+268)	38255 (+268)
**13**	38195 (+208)*, 38212 (+225)**	38195 (+208)
**14**	38175 (+188)*, 38191 (+204)**	38175 (+188)
**15**	38251 (+264)	38251 (+264)
**16**	38391 (+404)	38391 (+404)
**17**	38377 (+390)	38377 (+390), 38767 (+780)
**18**	38353 (+366)	37987 (+0)
**19**	38465 (+478)	38034 (+47), 38348 (+361)
**20**	38613 (+626)	38031 (+44), 38202 (+215), 38552 (+565), 38613 (+626)
**21**	38419 (+432)	38031 (+44), 38132 (+145), 38384 (+397), 38460 (+473), 38565 (+578)

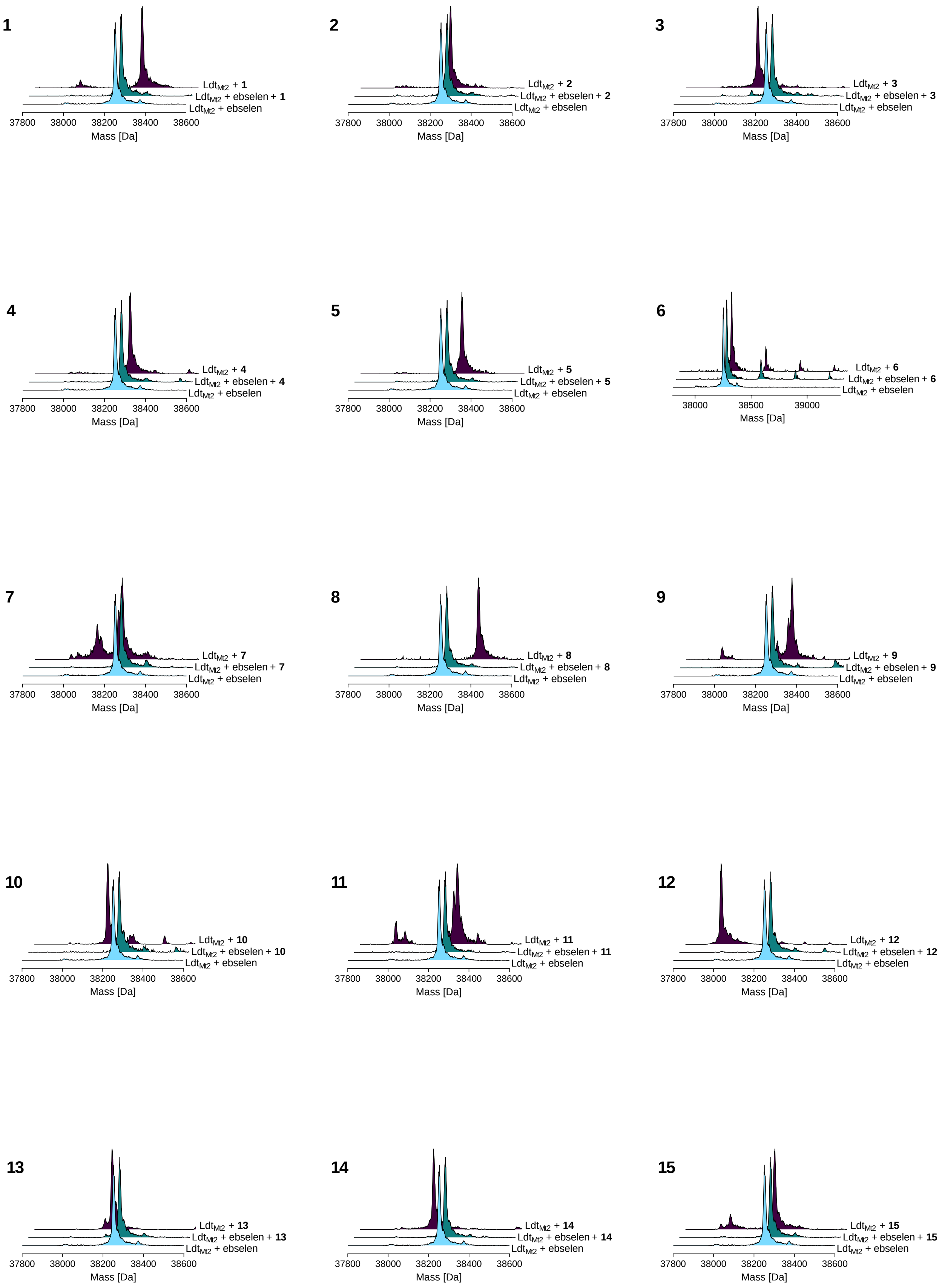

Figure S11. Protein observed SPE-MS based active-site selectivity assays (figure continues).

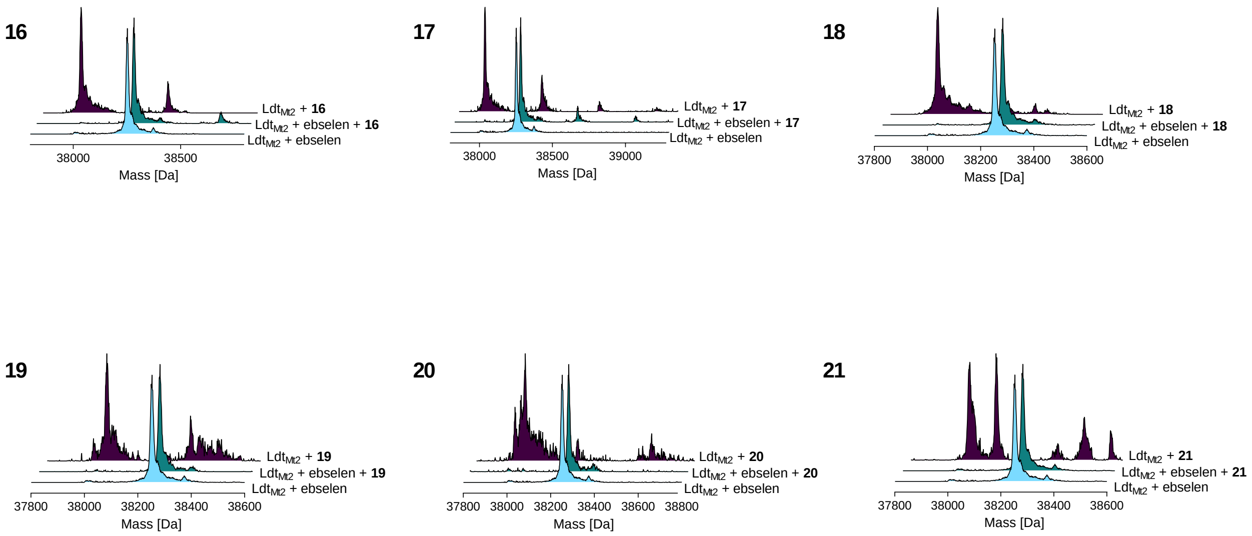

Figure S11. Protein observed SPE-MS based active-site selectivity assays. Ldt$_{Mt2}$ (1 µM) was preincubated with ebselen (10 µM, a known Ldt$_{Mt2}$ inhibitor[3], which reacts with the nucleophilic Cys354) for 1 h in 50 mM Tris, pH 7.5, prior to addition of inhibitors **1**-**15** (100 µM). Samples were analysed after an additional 24 h incubation at room temperature. The spectrum in blue corresponds to Ldt$_{Mt2}$ reacted with ebselen. The spectrum in green corresponds to Ldt$_{Mt2}$ reacted with the specified inhibitor, following preincubation with ebselen. The spectrum in purple corresponds to Ldt$_{Mt2}$ reacted with the specified inhibitor. Deconvoluted spectra, obtained using the maximum entropy algorithm in the MassHunter Workstation Qualitative Analysis B.07.00 program (Agilent), are shown.

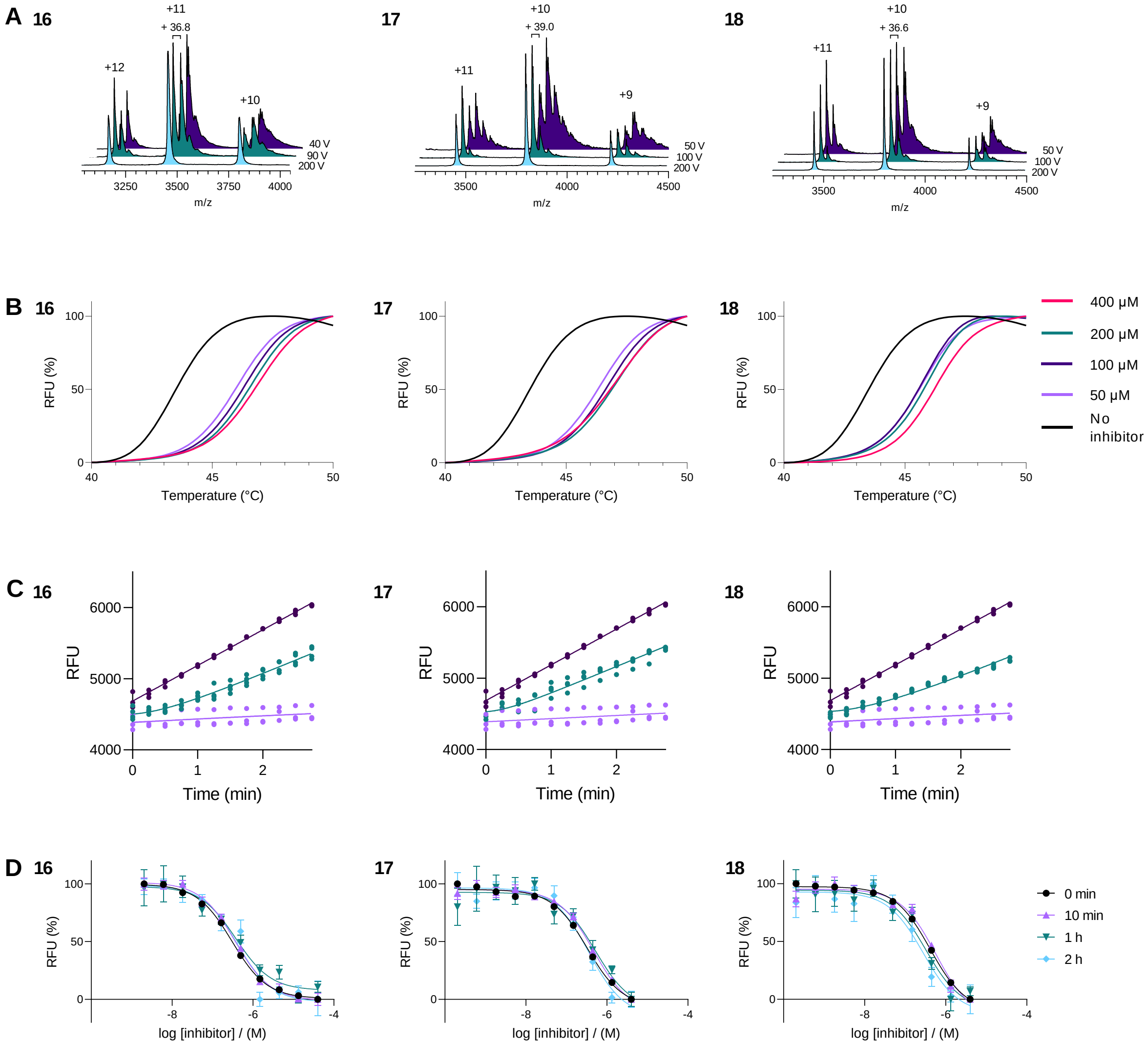

Figure S12. **Characterisation of the inhibition of Ldt$_{Mt2}$ by isatins 16-18. A.** Non-denaturing MS analyses of Ldt$_{Mt2}$ reaction with isatins **16**, **17**, and **18**. Ldt$_{Mt2}$ (5 µM) was combined with **16**, **17** or **18** (500 µM) in 500 mM ammonium acetate. Mass spectra were recorded at cone voltages ranging from 40 V to 200 V. The mass shifts induced by inhibitor binding are shown for the +11 charge state (**16**) or the +10 charge state (**17** and **18**) and correspond to the binding of a single inhibitor molecule. **B.** Thermal shift assays with isatins **16**-**18**. Ldt$_{Mt2}$ (5 µM) and the specified inhibitor (500 µM) were preincubated for 30 minutes prior to addition of SYPRO Orange (6 x concentrated, according to the manufacturer's (Invitrogen) protocol) in 50 mM Tris, pH 7.5. **C.** Jump dilution assays with isatins **16**-**18**. Ldt$_{Mt2}$ (10 µM) and the specified inhibitor (100 µM) in 50 mM HEPES, pH 7.2, 0.01% (*v/v*) Triton X-100 were incubated for 2 hours at room temperature, then 1000 x diluted and assayed with probe **2** (15 µM). The results imply that binding of the tested isatin inhibitors is reversible. The k_{off} and $t_{1/2}$ values are given in Table S7. **D**. Dose-response assays with isatins **16**-**18** at varying inhibitor pre-incubation times. Assays of Ldt$_{Mt2}$ were carried out with 300 nM Ldt$_{Mt2}$ and 15 µM probe **1** at room temperature in 50 mM sodium phosphate pH 7.5 with 0.007% (*v/v*) Tween-20. Average pIC$_{50}$ values are given in Table S8.

Table S7. Off rate (k_{off}) and half-life ($t_{1/2}$) of isatins 16 – 18 in complex with Ldt_{Mt2}, as determined by jump dilution assays.

Compound	k_{off} (s^{-1})	$t_{1/2}$ (min)
**16**	103 ± 16.5	0.41 ± 0.17
**17**	84.6 ± 6.03	0.49 ± 0.094
**18**	160 ± 15.5	0.26 ± 0.17

Table S8. Dose-response assays with isatins 16-18 at varying inhibitor pre-incubation times.

Compound	pIC_{50}			
	**0 min**	**10 min**	**60 min**	**120 min**
**16**	6.52 ± 0.06	6.44 ± 0.11	6.43 ± 0.32	6.39 ± 0.37
**17**	6.48 ± 0.14	6.33 ± 0.14	6.28 ± 0.45	6.45 ± 0.26
**18**	6.39 ± 0.07	6.24 ± 0.23	6.49 ± 0.35	6.56 ± 0.35

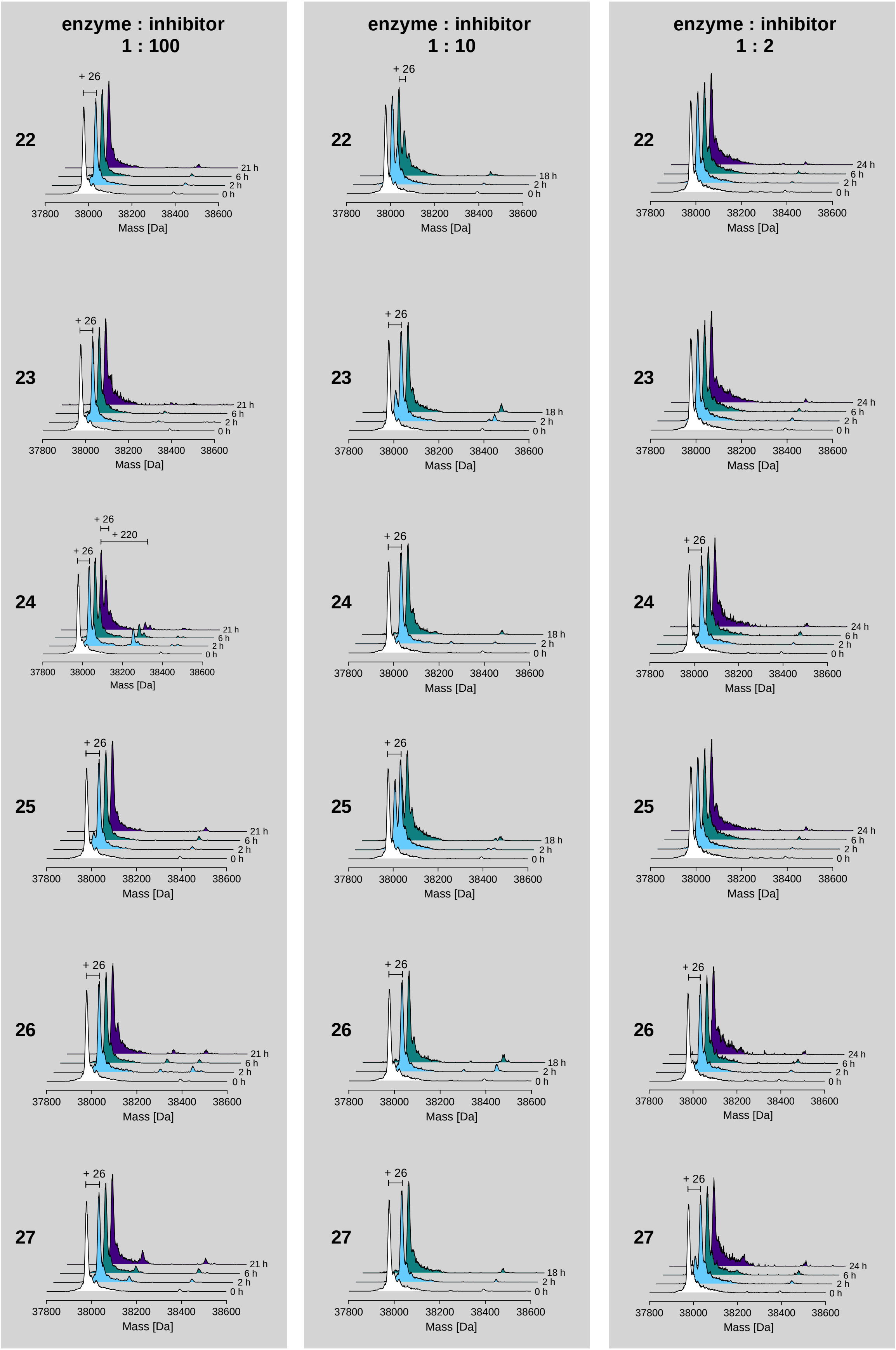

Figure S13. Protein observed SPE-MS analysis for the reaction of Ldt$_{Mt2}$ with inhibitors 22-39 (figure continues).

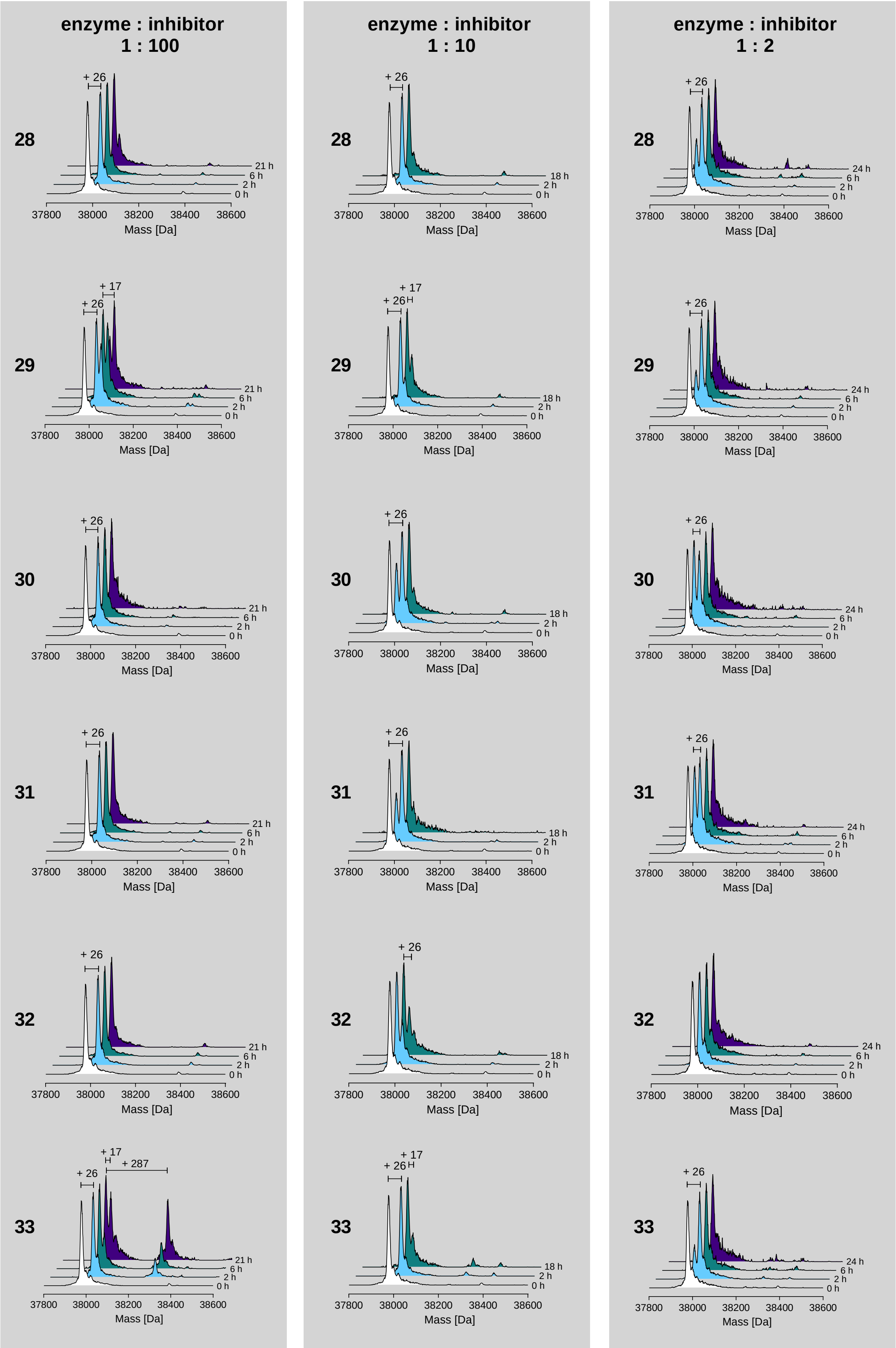

Figure S13. Protein observed SPE-MS analysis for the reaction of Ldt_{Mt2} with inhibitors 22-39 (figure continues).

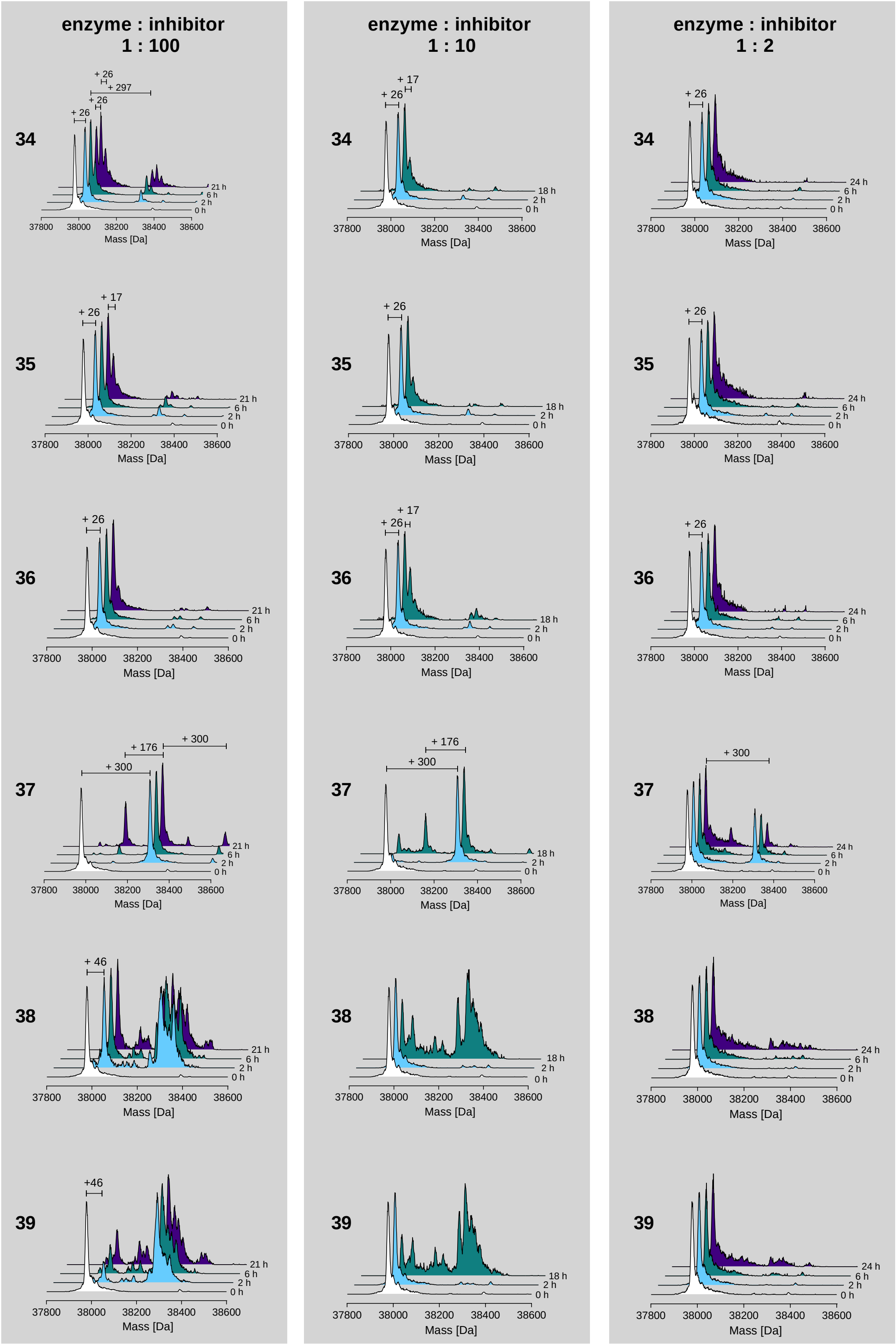

Figure S13. Protein observed SPE-MS analysis for the reaction of Ldt$_{Mt2}$ with inhibitors 22-39. 1 µM Ldt$_{Mt2}$ was incubated with the inhibitor at the specified ratio at room temperature in 50 mM Tris, pH 7.5. Samples were analysed after the indicated times. Compound structures are given in Table S5. Deconvoluted spectra, obtained using the maximum entropy algorithm in the MassHunter Workstation Qualitative Analysis B.07.00 program (Agilent), are shown.

Table S9. Calculated* and observed masses (Da) and mass shifts (Da) from protein observed SPE-MS experiments with Ldt_{Mt2} (1 µM) and the indicated nitrile inhibitors. Mass shifts are relative to unmodified Ldt_{Mt2}. *Calculated mass for cyanation of Ldt_{Mt2}. **Calculated mass for unfragmented adduct formation. ***Due to complexity of the obtained mass spectra, only major observed peaks are provided.

Compound	Calculated mass (Da)	Observed mass (Da)
**22**	38004 (+26)*, 38283 (+305)**	38004 (+26)
**23**	38004 (+26)*, 38283 (+305)**	38004 (+26)
**24**	38004 (+26)*, 38199 (+221)**	38004 (+26), 38030 (+52), 38224 (+246)
**25**	38004 (+26)*, 38199 (+221)**	38004 (+26)
**26**	38004 (+26)*, 38249 (+271)**	38004 (+26)
**27**	38004 (+26)*, 38222 (+244)**	38004 (+26)
**28**	38004 (+26)*, 38206 (+228)**	38004 (+26)
**29**	38004 (+26)*, 38280 (+302)**	38004 (+26), 38022 (+44)
**30**	38004 (+26)*, 38294 (+316)**	38004 (+26)
**31**	38004 (+26)*, 38281 (+303)**	38004 (+26)
**32**	38004 (+26)*, 38297 (+319)**	38004 (+26)
**33**	38004 (+26)*, 38264 (+286)**	38004 (+26), 38022 (+44), 38291 (+313)
**34**	38004 (+26)*, 38275 (+297)**	38004 (+26), 38030 (+52), 38056 (+78), 38301 (+323), 38327 (+349), 38353 (+375)
**35**	38004 (+26)*, 38275 (+297)**	38004 (+26), 38022 (+44)
**36**	38004 (+26)*, 38302 (+324)**	38004 (+26), 38022 (+44)
**37**	38004 (+26)*, 38278 (+300)**	38102 (+124), 38278 (+300), 38402 (+424), 38578 (+ 600)
**38**	38319 (+341)**	38024 (+46), 38277 (+299), 38293 (+315), 38331 (+353)***
**39**	38305 (+327)**	38024 (+46), 38125 (+147), 38157 (+179), 38252 (+274), 38277 (+299), 38296 (+318), 38316 (+338)***

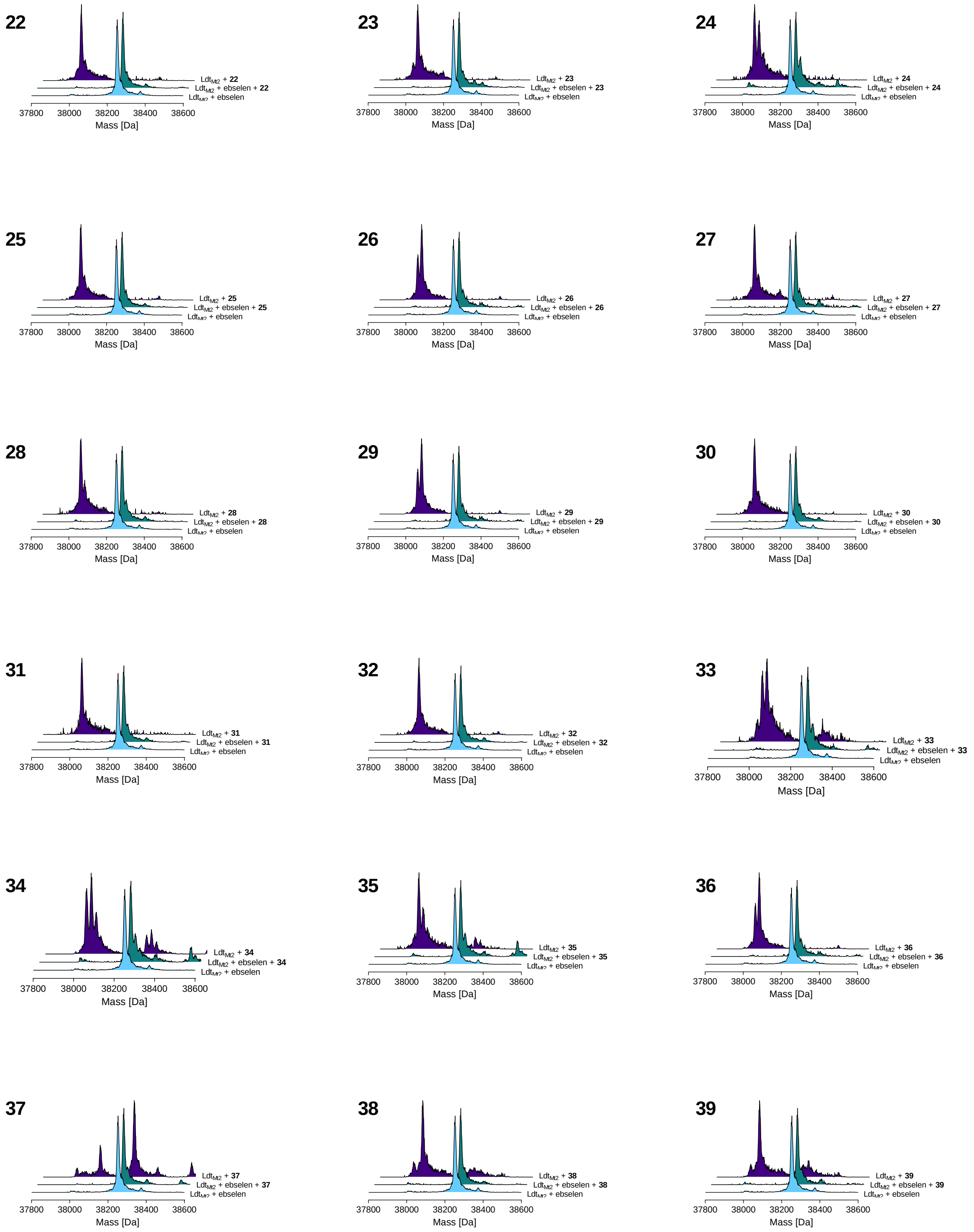

Figure S14. Protein observed SPE-MS based active-site selectivity assays with nitriles 22-36. Ldt$_{Mt2}$ (1 µM) was preincubated with ebselen (10 µM, a known Ldt$_{Mt2}$ inhibitor[3], which reacts with the nucleophilic Cys354) for 1 h in 50 mM Tris, pH 7.5, prior to addition of inhibitors **22**-**36** (100 µM). Samples were analysed after an additional 24 h incubation at room temperature. The spectrum in blue corresponds to Ldt$_{Mt2}$ in complex with ebselen. The spectrum in green corresponds to Ldt$_{Mt2}$ and the specified inhibitor upon preincubation with ebselen. The spectrum in purple corresponds to Ldt$_{Mt2}$ in complex with the specified inhibitor. Deconvoluted spectra, obtained using the maximum entropy algorithm in the MassHunter Workstation Qualitative Analysis B.07.00 program (Agilent), are shown.

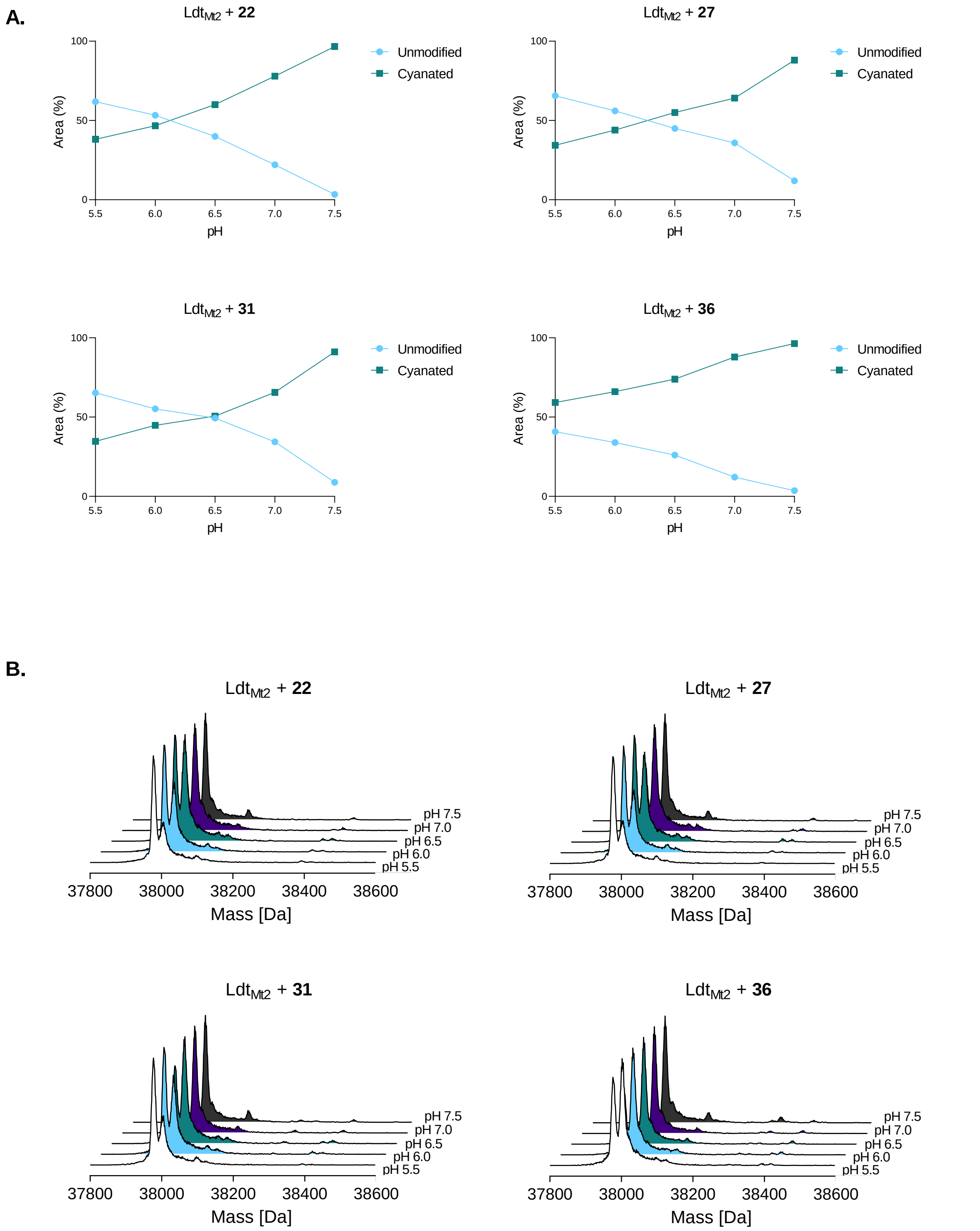

Figure S15. pH dependence of the cyanation of Ldt$_{Mt2}$ by representative cyanamides 22, 27, 31 and 36. MS spectra were taken 15 min after addition of the nitrile (10 µM) to Ldt$_{Mt2}$ (1 µM) in 50 mM Tris, pH 7.5. **(A)** Area of cyanated and unmodified Ldt$_{Mt2}$ peaks at the specified pH. **(B)** MS spectra of Ldt$_{Mt2}$ following reaction with selected cyanamides at the specified pH values.

Table S10. Inhibition of Ldt_{MT2} by known cyanating compounds.[4, 5] MW: Molecular weight. k_{chem}: apparent intrinsic thiol reactivity as measured with *L*-glutathione and probe **2**.

	Structure	MW	pIC_{50} Ldt_{Mt2}	k_{chem}
**40**	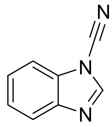	143.15	6.31 ± 0.03	0.90 ± 0.05
**41**	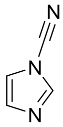	93.09	6.54 ± 0.01	3.69 ± 0.11
**42**	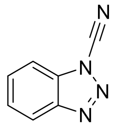	144.14	6.02 ± 0.05	0.90 ± 0.08
**43 (CDAP)**	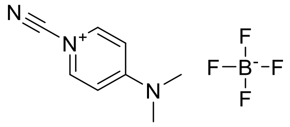	234.99	5.30 ± 0.08	N.D.
**44 (NTCB)**	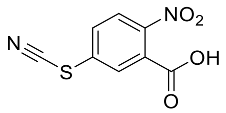	224.19	5.84 ± 0.04	N.D.
**45 (CDTP)**	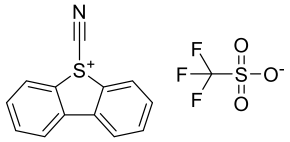	359.34	<4.4	N.D.

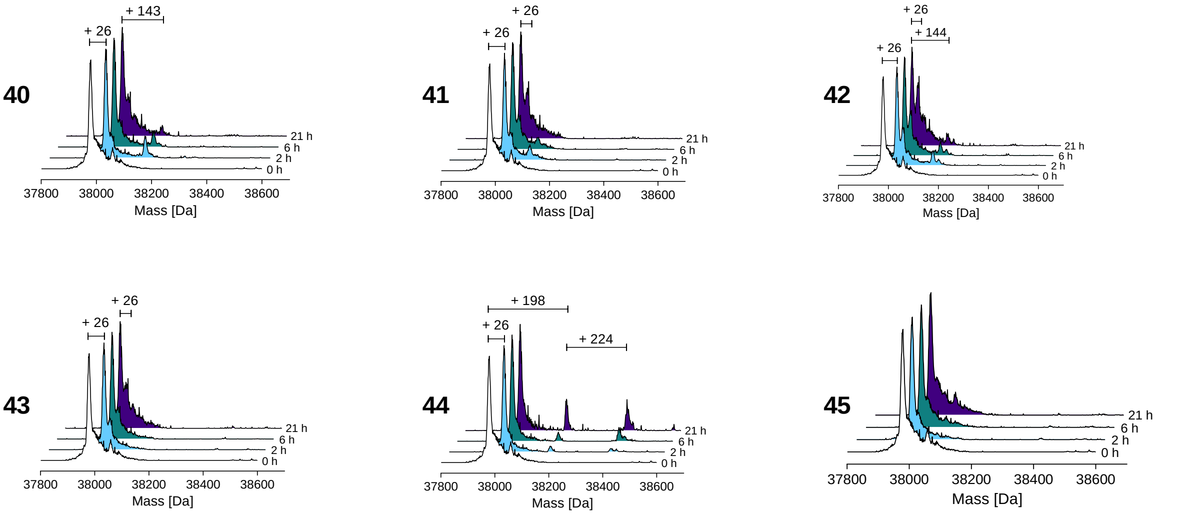

Figure S16. Protein observed SPE-MS analysis for the reaction of Ldt_{Mt2} with cyanating agents 40-45. 1 µM Ldt_{Mt2} was incubated with 100 µM cyanating agent at room temperature in 50 mM Tris, pH 7.5. Samples were analysed after the indicated times. Compound structures are given in Table S10. Deconvoluted spectra, obtained using the maximum entropy algorithm in the MassHunter Workstation Qualitative Analysis B.07.00 program (Agilent), are shown.

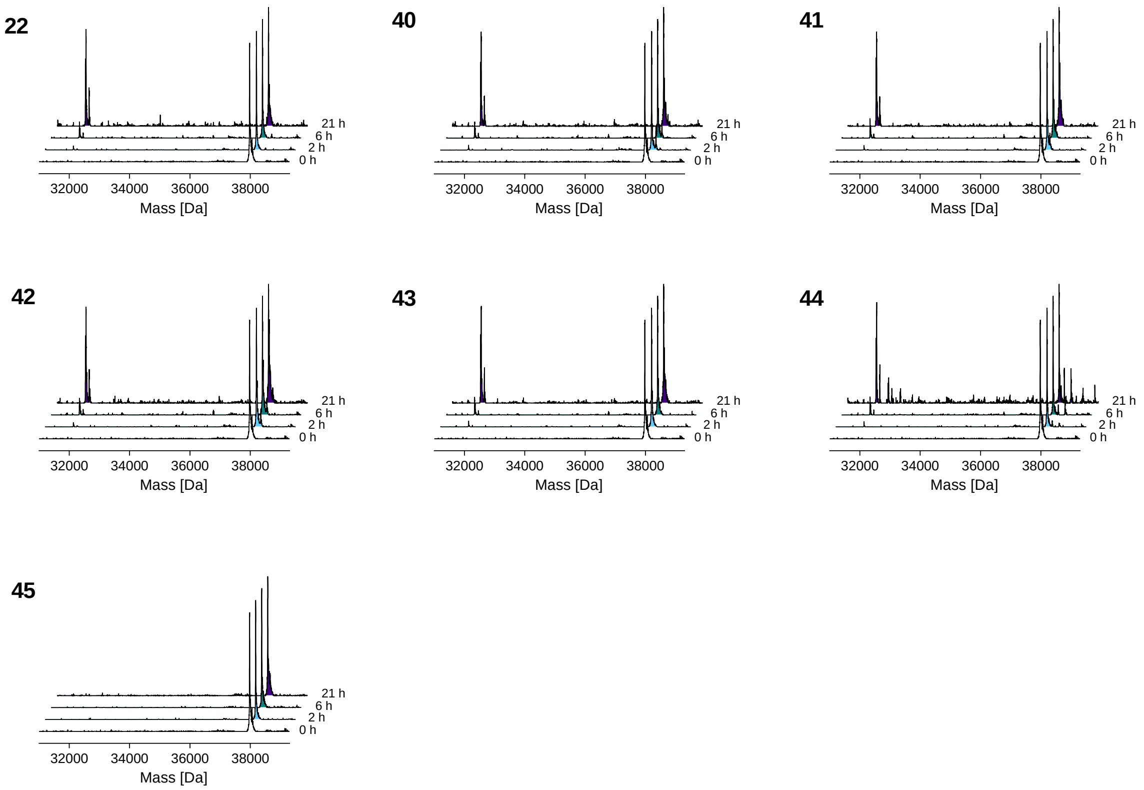

Figure S17. Protein observed SPE-MS analysis of the reaction of Ldt_{Mt2} with 22 (a representative of cyanamides 22-36) and 40-45, showing evidence for cleavage of Ldt_{Mt2} at Cys354 after prolonged incubation for 22 and 40-44. 1 µM Ldt_{Mt2} was incubated with the inhibitor at a 1 to 100 ratio at room temperature in 50 mM Tris, pH 7.5. Samples were analysed after the indicated times. Compound structures are given in Table S5 and Table S9. Deconvoluted spectra, obtained using the maximum entropy algorithm in the MassHunter Workstation Qualitative Analysis B.07.00 program (Agilent), are shown. Note, cleavage was not observed in the case of compound **45**.

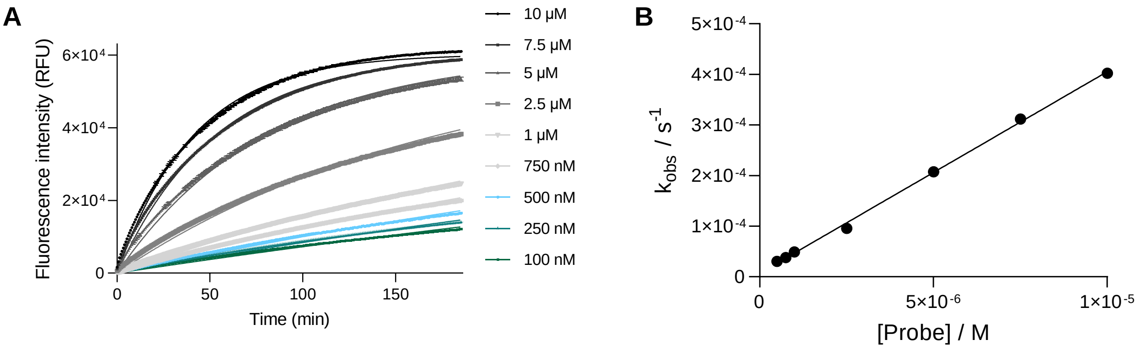

Figure S18. Assay set up for the determination of the second-order rate constant k_{inact}/K_I for irreversible inactivation of Ldt_{Mt2} via an endpoint assay using irreversibly reacting Probe 2. A. Determination of the observed kinetic rate constant (k_{obs}). Assays were carried out using 100 nM Ldt_{Mt2} at room temperature in 50 mM HEPES, pH 7.2 with 0.01% (*v/v*) Triton X-100, using varying Probe 2 concentrations, as specified. **B.** The obtained k_{obs} values at various Probe 2 concentrations were used to provide the $(k_{inact}/K_I)_{probe}$ value.

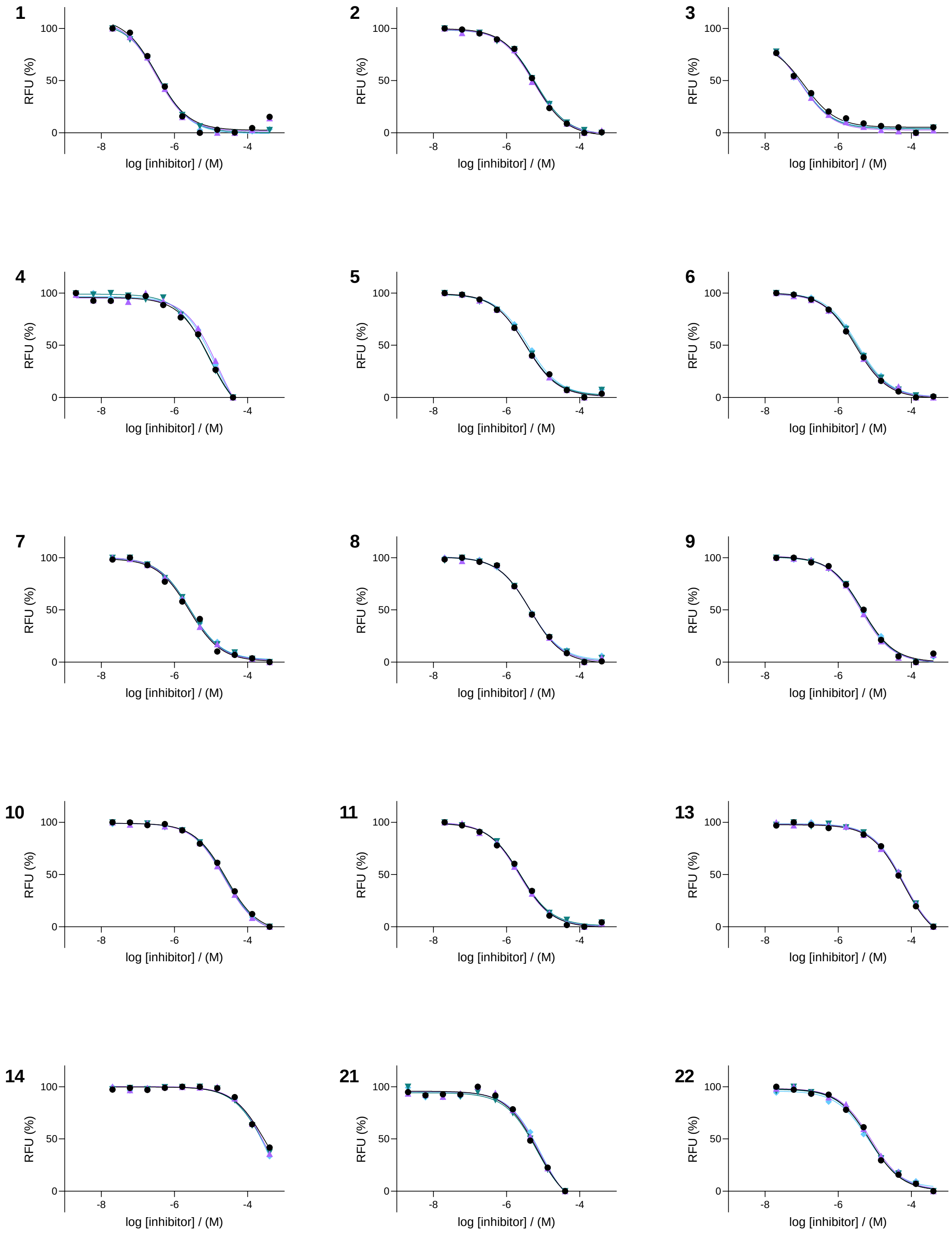

Figure S19. Determination of the second-order rate constant for covalent target inactivation (k_{inact}/K_I) of covalent inhibitors 1-11, 13-14 and 21-37 (figure continues).

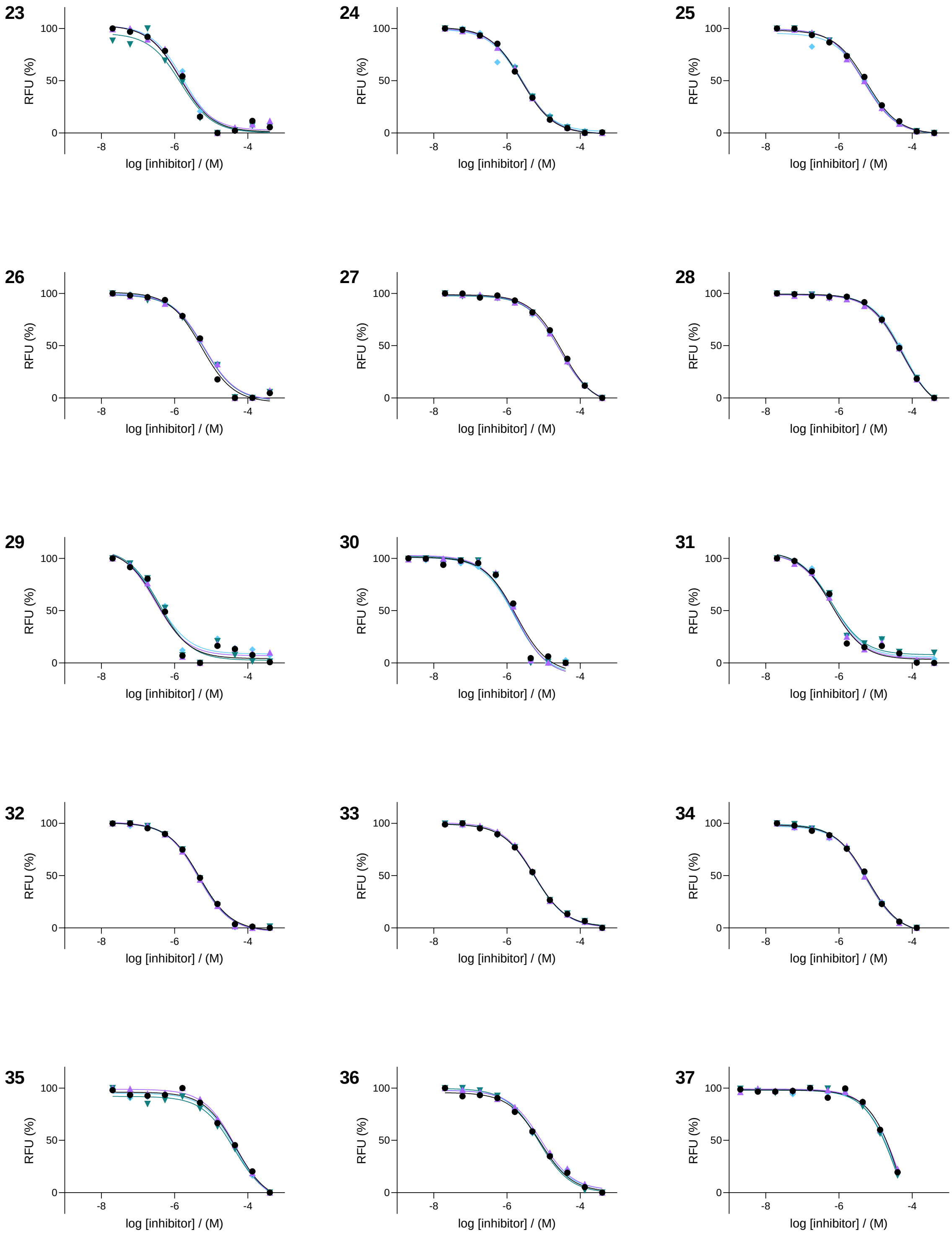

Figure S19. Determination of the second-order rate constant for covalent target inactivation (k_{inact}/K_I) of covalent inhibitors 1-11, 13-14 and 21-37. Inhibition assays were carried out using 100 nM Ldt_{Mt2} and 10 µM Probe **2** with 3 h incubation at rt in 50 mM HEPES, pH 7.2 with 0.01% (*v/v*) Triton X-100. Data points represent values of independent repeats (n=4). Average $(k_{inact}/K_I)_{inhibitor}$ values and compound structures are given in Table S5.

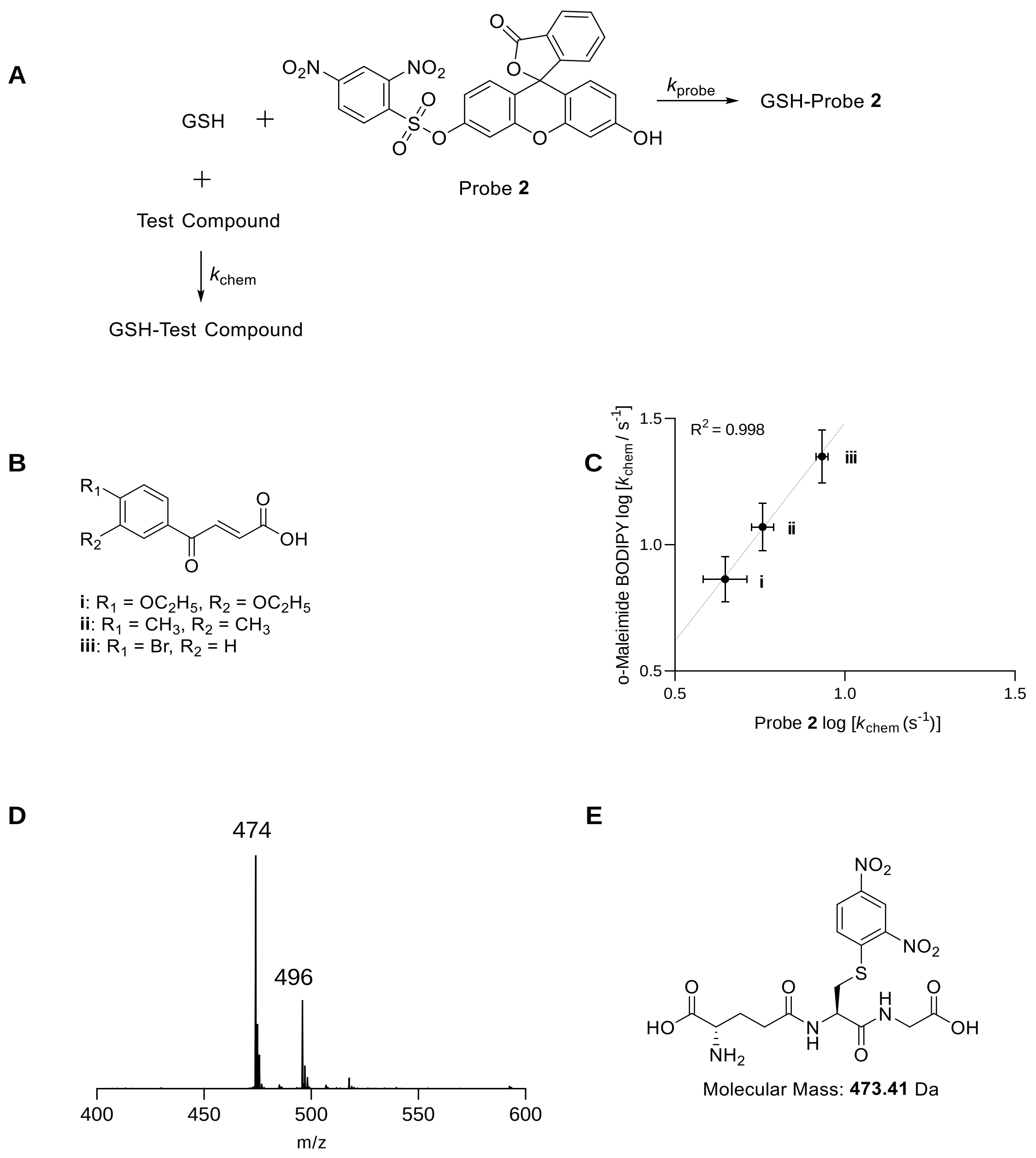

Figure S20. Analysis of the intrinsic thiol reactivity (*k*$_{chem}$) assay. (A) The assay measures competition between glutathione (GSH) and test compounds for reaction with probe **2**. **(B)** Compounds **i-iii** used for validation of the *k*$_{chem}$ assay. **(C)** *k*$_{chem}$ of **i-iii** obtained from the fluorogenic assay with probe **2** versus the *k*$_{chem}$ values reported from the fluorogenic assay with *o*-maleimide BODIPY.[6] **(D)** GSH reacts covalently and irreversibly with probe **2** under the tested conditions. GSH (2 µM) was incubated with probe **2** (200 µM) for 16 hours in 50 mM tris, pH 7.5, prior to addition of *N*-ethylmaleimide (2 mM) and incubation of an additional 24 h. Only the mass of GSH modified with probe **2** ($[M+H]^+$ 474, $[M+Na]^+$ 496) was observed. **(E)** Structure and molecular mass of the anticipated product from the reaction of GSH and probe **2**.

Table S11. k_{chem} of i-iii obtained from the fluorogenic assay with probe 2 versus the k_{chem} values reported from the fluorogenic assay with *o*-maleimide BODIPY.[6]

Compound	log(k_{chem}) probe 2 (mean ± SD)	log(k_{chem}) *o*-maleimide BODIPY (mean ± SD)
**i**	0.65 ± 0.06	0.86 ± 0.09
**ii**	0.76 ± 0.03	1.07 ± 0.09
**iii**	0.93 ± 0.02	1.34 ± 0.10

Table S12. Inhibitory activity of selected hit compounds against *M. tuberculosis* and cytotoxicity in HepG2 cells. MIC_{50} intracellular values represent the concentration of inhibitor that inhibited *M. tuberculosis* (strain H37Rv) growth in THP1 cells by 50%, as obtained from extrapolation of the dose-response curve. MIC_{50} extracellular values represent the concentration of inhibitor that inhibited *M. tuberculosis* (strain H37Rv) growth by 50%, as obtained from extrapolation of the dose-response curve.

	Structure	MIC_{50} intracellular (µM)	MIC_{50} extracellular (µM)	HepG2 IC_{50} (µM)
**1**	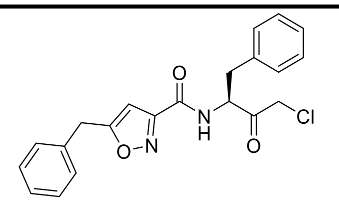	35.5 ± 4.9	>100	N.D.
**5**	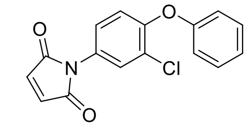	25.7 ± 0.30	>100	2.51 ± 0.12
**7**	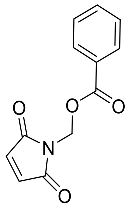	39.8 ± 0.0	>100	3.90 ± 0.88
**15**	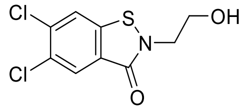	7.08 ± 0.16	>100	9.55 ± 0.22
**17**	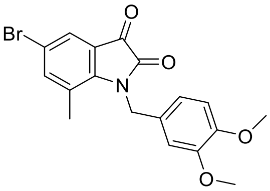	15.1 ± 0.70	>100	53.5 ± 6.7
**18**	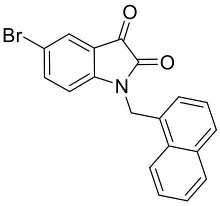	35.5 ± 5.4	>100	42.3 ± 3.4
**20**	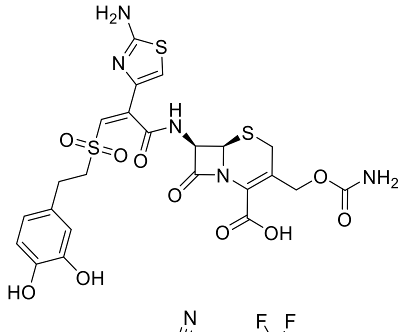	38.9 ± 1.8	>100	>100
**23**	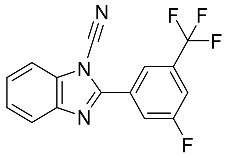	12.0 ± 1.5	>100	28.8 ± 0.0

	Structure	MIC$_{50}$ intracellular (µM)	MIC$_{50}$ extracellular (µM)	HepG2 IC$_{50}$ (µM)
**30**	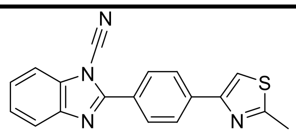	0.98 ± 1.2	>100	3.98 ± 0.10
**36**	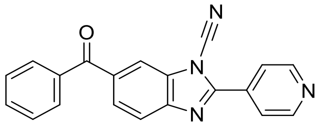	29.5 ± 0.0	>100	32.7 ± 0.38
**39**	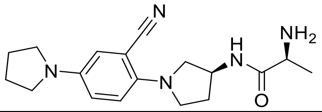	28.8 ± 0.0	>100	>100

Table S13. Data collection and refinement statistics for Ldt_{Mt2} inhibitor crystal structures.

**Datasets**	**Ldt_{Mt2} – 2 (PDB: 8A1L)**	**Ldt_{Mt2} – 3 (PDB: 8A1J)**	**Ldt_{Mt2} – 4 (PDB: 8A1M)**
**Data Collection**			
Beamline (Wavelength, Å)	DLS I03 (0.9763)	DLS I03 (0.9763)	DLS I03 (0.9763)
Detector	Eiger2 XE 16M	Eiger2 XE 16M	Eiger2 XE 16M
Data Processing	Xia2 dials	Xia2 3dii	Xia2 3dii
Space group	$P\ 1\ 2_1\ 1$	$P\ 1\ 2_1\ 1$	$P\ 1\ 2_1\ 1$
Cell dimensions			
a,b,c (Å)	61.76, 94.77, 75.74	61.19, 94.02, 75.29	61.70, 95.07, 75.45
α, β, γ (°)	90.00, 92.90, 90.00	90.00, 92.61, 90.00	90.00, 92.22, 90.00
No. of molecules/ASU	2	2	2
No. reflections	38420 (1817)*	27561 (1234)*	38184 (1886)*
Resolution (Å)	61.68-2.30 (2.34-2.30)*	47.01-2.54 (2.58-2.54)*	48.66-2.30 (2.34-2.30)*
R_{merge} (I)	0.162 (1.331)*	0.237 (1.859)*	0.131 (1.291)*
I/σI	9.8 (1.0)*	7.4 (1.0)*	9.6 (1.4)*
CC-1/2	1.0 (0.8)*	1.0 (0.6)*	1.0 (0.6)*
Completeness (%)	99.6 (96.1)*	97.9 (89.7)*	98.5 (98.7)*
Multiplicity	6.9 (7.2)*	6.8 (5.8)*	6.9 (6.8)*
Wilson B value ($Å^2$)	33.84	31.08	40.55
**Refinement**	PHENIX	PHENIX	PHENIX
R_{work}/R_{free}	0.2146/0.2476	0.2346/0.2663	0.2179/0.2501
No. atoms	5775	5616	5782
- Enzyme	5276	5231	5319
- Ligand	76	46	81
- Water	423	339	382
Average B-factors	47.06	52.20	53.20
- Enzyme	46.99	52.46	53.17
- Ligand	55.35	58.79	66.73
- Water	46.40	47.23	50.85
RMS[$] deviations			
- Bond lengths (Å)	0.003	0.003	0.003
- Bond angles (°)	0.51	0.58	0.53

[#] ASU = asymmetric unit.

[$] RMS = root mean square.

*Highest resolution shell in parentheses.

**Datasets**	**Ldt_{Mt2} – 8 (PDB: 8A1O)**	**Ldt_{Mt2} – 13 (PDB: 8A1N)**	**Ldt_{Mt2} – 15 (PDB: 8A1K)**
**Data Collection**			
Beamline (Wavelength, Å)	DLS I03 (0.9763)	DLS I03 (0.9763)	DLS I03 (0.9763)
Detector	Eiger2 XE 16M	Eiger2 XE 16M	Eiger2 XE 16M
Data Processing	Xia2 dials	Xia2 dials	Xia2 dials
Space group	$P\ 2_1\ 2_1\ 2$	$P\ 1\ 2_1\ 1$	$P\ 1\ 2_1\ 1$
Cell dimensions			
a,b,c (Å)	76.87, 93.00, 61.40	60.93, 94.63, 75.36	60.98, 95.17, 75.62
α, β, γ (°)	90.00, 90.00, 90.00	90.00, 92.35, 90.00	90.00, 92.35, 90.00
No. of molecules/ASU	1	2	2
No. reflections	31561 (1582)*	53680 (2613)*	86822 (4298)*
Resolution (Å)	46.50-1.95 (1.98-1.95)*	75.29-2.05 (2.08-2.05)*	60.93-1.75 (1.78-1.75)*
R_{merge}(I)	0.066 (0.593)*	0.090 (0.826)*	0.063 (0.759)*
I/σI	24.2 (3.5)*	13.2 (1.1)*	17.7 (1.0)*
CC-1/2	1.0 (0.9)*	1.0 (0.8)*	1.0 (0.8)*
Completeness (%)	96.2 (97.1)*	99.9 (97.9)*	100.0 (99.3)*
Multiplicity	12.6 (11.5)*	6.8 (6.8)*	6.6 (6.6)*
Wilson B value (Å^2)	24.43	31.68	23.14
**Refinement**	PHENIX	PHENIX	PHENIX
R_{work}/R_{free}	0.1939/0.2228	0.1954/0.2379	0.1837/0.2107
No. atoms	3166	5937	6401
- Enzyme	2696	5296	5367
- Ligand	42	42	43
- Water	428	599	991
Average B-factors	37.22	44.34	34.55
- Enzyme	35.97	44.07	32.96
- Ligand	58.19	53.53	40.01
- Water	43.07	46.05	42.92
RMS[$] deviations			
- Bond lengths (Å)	0.010	0.003	0.011
- Bond angles (°)	0.88	0.58	0.94

[#] ASU = asymmetric unit.

[$] RMS = root mean square.

*Highest resolution shell in parentheses.

**Datasets**	**Ldt$_{Mt2}$ – 31 (PDB: 8AHO)**
**Data Collection**	
Beamline (Wavelength, Å)	DLS I03 (0.9763)
Detector	Eiger2 XE 16M
Data Processing	Xia2 dials
Space group	P 1 2_1 1
Cell dimensions	
a,b,c (Å)	61.25, 94.26, 75.67
α, β, γ (°)	90.00, 92.69, 90.00
No. of molecules/ASU	2
No. reflections	38141 (3764)*
Resolution (Å)	48.69-2.30 (2.38-2.30)*
R_{merge}(I)	0.136 (0.781)*
I/σI	8.9 (1.3)*
CC-1/2	1.0 (0.9)*
Completeness (%)	99.8 (98.4)*
Multiplicity	6.9 (6.8)*
Wilson B value (Å^2)	39.27
**Refinement**	PHENIX
R_{work}/R_{free}	0.2101/0.2495
No. atoms	5701
- Enzyme	5267
- Ligand	41
- Water	393
Average B-factors	45.27
- Enzyme	45.18
- Ligand	66.22
- Water	44.40
RMS$^\$$ deviations	
- Bond lengths (Å)	0.002
- Bond angles (°)	0.54

[#] ASU = asymmetric unit.

[$] RMS = root mean square.

*Highest resolution shell in parentheses.

Experimental details for the HTS assay optimisation process

Optimisation of reagent concentrations and reaction volume

The specified volume of assay buffer (50 mM sodium phosphate pH 7.5 with 0.01% (*v/v*) Triton X-100) was added to a black polystyrene, flat-bottomed, small volume, clear bottomed 384-well microplate (Greiner Bio-One, part number 784076) using a MultiDrop Combi dispenser (ThermoFisher Scientific). To this was added the specified concentrations of Ldt_{Mt2} and probe **1**.[1] The fluorescence intensity was measured for the specified time using an Envision 2104 Multilabel Reader (Perkin Elmer) with λ_{ex}=485 nm, λ_{em}=540 nm and a general dual mirror. All reactions were carried out in quadruplicate, with no-enzyme control measurements included.

DMSO tolerance assay

A Ldt_{Mt2} stock solution of 600 nM (2x final concentration 300 nM) was prepared in the assay buffer (50 mM sodium phosphate pH 7.5 with 0.01% (*v/v*) Triton X-100). A solution of probe **1** (30 µM, 2x final concentration 15 µM) was prepared in the assay buffer. A dilution series of DMSO ranging from 33.3% to 0.003% (*v/v*) was added to a black polystyrene, flat-bottomed, small volume, clear bottomed 384-well microplate (Greiner Bio-One, part number 784076) using a HP D300 Digital Dispenser. To each well was added 5 µL of the 2x Ldt_{Mt2} stock and 5 µL of the 2x probe **1** stock using a MultiDrop Combi dispenser (ThermoFisher Scientific). The fluorescence intensity was measured every minute for 60 minutes using an Envision 2104 Multilabel Reader (Perkin Elmer) with λ_{ex}=485 nm, λ_{em}=540 nm and a general dual mirror. All reactions were carried out in quadruplicate, with no-enzyme control and no DMSO control measurements included.

Detergent optimisation

An Ldt_{Mt2} stock solution of 600 nM (2x final concentration 300 nM) was prepared in the assay buffer (50 mM sodium phosphate pH 7.5). Stock solutions of 20 x critical micelle concentration (CMC) of the detergents Triton X-100, Triton X-114, Tween-20 (Thermo Scientific), Tween-80, MEGA-8, CHAPS, BRIJ-35, IPEGAL-630, n-dodecyl-β-D-maltose, BSA and dBSA (prepared by incubation of BSA for 15 minutes at 80 °C, followed by 2 hours at room temperature and stored at -20 °C) were prepared. Using a HP D300 Digital Dispenser 11 concentrations of ½ step dilutions were added to a black polystyrene, flat-bottomed, small volume, clear bottomed 384-well microplate (Greiner Bio-One, part number 784076). To each well was added 5 µL of the 2x Ldt_{Mt2} stock and 5 µL of the 2x probe **1** stock solution using a MultiDrop Combi dispenser (ThermoFisher Scientific). The fluorescence intensity was measured every minute for 60 minutes using an Envision 2104 Multilabel Reader (Perkin Elmer) with λ_{ex}=485 nm, λ_{em}=540 nm and a general dual mirror. All reactions were carried out in quadruplicate, with no-enzyme control and no detergent control measurements included. Maximum tolerated concentrations were calculated by analysing the resulting slopes using Microsoft Excel. Buffers of the detergents in maximum tolerated concentrations, or 1 x CMC where no interference was observed, in 50 mM sodium phosphate pH 7.5 were prepared, omitting BRIJ-35, IPEGAL-630, BSA and dBSA due to interference at all concentrations. With each buffer, a Ldt_{Mt2} stock of 600 nM (2x final concentration 300 nM) and a probe **1** stock solution of 30 µM (2x final concentration 15 µM) was prepared. To each well was added 5 µL of the 2x Ldt_{Mt2} stock solution and 5 µL of the 2x probe **1** stock solution using a MultiDrop Combi dispenser (ThermoFisher Scientific). The fluorescence intensity was measured every minute for 60 minutes using a Envision 2104 Multilabel Reader (Perkin Elmer) with λ_{ex}=485 nm, λ_{em}=540 nm and a

general dual mirror. All reactions were carried out in quadruplicate, with no-enzyme control measurements included.

Plate reader protocol optimisation

A stock solution of Ldt_{Mt2} of 600 nM (2x final concentration 300 nM) was prepared in the assay buffer (50 mM sodium phosphate pH 7.5 with 0.007% (*v/v*) Tween-20). A stock solution of probe **1** of 30 µM (2x final concentration 15 µM) was prepared in the assay buffer. To each well was added 5 µL of the 2x Ldt_{Mt2} stock solution and 5 µL of the 2x probe **1** stock solution using a MultiDrop Combi dispenser (ThermoFisher Scientific). The fluorescence intensity was measured every minute for 60 minutes using a Envision 2104 Multilabel Reader (Perkin Elmer) with λ_{ex}=485 nm, λ_{em}=540 nm and a FITC mirror. The plate reader protocol was optimised for the coordinates of the corners, measurement height and gain.

Stability of stock solution concentrations

A stock solution of Ldt_{Mt2} of 600 nM (2x final concentration 300 nM) was prepared in the assay buffer (50 mM sodium phosphate pH 7.5 with 0.007% (*v/v*) Tween-20). A stock solution of probe **1** of 30 µM (2x final concentration 15 µM) was prepared in the assay buffer. The stock solutions were kept on ice for 7 hours, after which time 5 µL of the 2x Ldt_{Mt2} solution and 5 µL of the 2x probe **1** solution were added to a black polystyrene, flat-bottomed, small volume, clear bottomed 384-well microplate (Greiner Bio-One, part number 784076) using a MultiDrop Combi dispenser (ThermoFisher Scientific). The fluorescence intensity was measured every minute for 60 minutes using an Envision 2104 Multilabel Reader (Perkin Elmer) with λ_{ex}=485 nm, λ_{em}=540 nm and a FITC mirror.

Full plate analysis

A stock solution of Ldt_{Mt2} of 600 nM (2x final concentration 300 nM) was prepared in the assay buffer (50 mM sodium phosphate pH 7.5 with 0.007% (*v/v*) Tween-20). A stock solution of probe **1** of 30 µM (2x final concentration 15 µM) was prepared in the assay buffer. To a black polystyrene, flat-bottomed, small volume, clear bottomed 384-well microplate (Greiner Bio-One, part number 784076) was added 100 nL of DMSO to all wells using a HP D300 Digital Dispenser. To this was added 5 µL of the 2x Ldt_{Mt2} stock solution and 5 µL of the 2x probe **1** stock solution using a MultiDrop Combi dispenser (ThermoFisher Scientific). The fluorescence intensity was measured every 5 minutes for 10 hours using a Envision 2104 Multilabel Reader (Perkin Elmer) with λ_{ex}=485 nm, λ_{em}=540 nm and a FITC mirror.

Dose-response analysis with tool compounds

Inhibitor solutions (in DMSO, concentrations ranging from 100 µM to 1.69 nM)) were added to a black polystyrene, flat-bottomed, small volume, clear bottomed 384-well microplate (Greiner Bio-One, part number 784076), with DMSO concentrations normalised to 1% (100 nL) in each well using a Hewlett-Packard D300 Digital Dispenser.

The so obtained plates were stored at -20 °C until use. A stock solution of Ldt_{Mt2} of 600 nM (2x final concentration 300 nM) was prepared in the assay buffer (50 mM sodium phosphate pH 7.5 with 0.007% (*v/v*) Tween-20). A stock solution of probe **1** of 30 µM (2x final concentration 15 µM) was prepared in the assay buffer. To the inhibitor plates was added 5 µL of the 2x Ldt_{Mt2} stock solution using a MultiDrop Combi dispenser (ThermoFisher Scientific). The resulting solution was incubated for 10 minutes at room temperature without shaking. 5 µL of the 2x probe **1** stock solution was then added using a MultiDrop Combi dispenser (ThermoFisher Scientific). The plate was incubated for another 10

minutes at room temperature. The fluorescence intensity was then measured 5 minute intervals for 50 minutes using an Envision 2104 Multilabel Reader (Perkin Elmer) with λ_{ex}=485 nm, λ_{em}=540 nm and a FITC mirror. The slope was calculated using linear regression analysis. The normalised response was calculated using the following formula: Normalized response = 100 * ((slope – slope control 2) / (slope control 1 – slope control 2)). The dose-response analysis was performed in Prism (GraphPad), using the log(inhibitor) versus normalized response− variable slope function.

References

1. de Munnik, M.; Lohans, C. T.; Langley, G. W.; Bon, C.; Brem, J.; Schofield, C. J., A Fluorescence-Based Assay for Screening β-Lactams Targeting the Mycobacterium tuberculosis Transpeptidase LdtMt2. *ChemBioChem* **2020,** *21*, 368-372.
2. Van Berkel, S. S.; Brem, J.; Rydzik, A. M.; Salimraj, R.; Cain, R.; Verma, A.; Owens, R. J.; Fishwick, C. W. G.; Spencer, J.; Schofield, C. J., Assay Platform for Clinically Relevant Metallo-β-lactamases. *J. Med. Chem.* **2013,** *56*, 6945-6953.
3. de Munnik, M.; Lohans, C. T.; Lang, P. A.; Langley, G. W.; Malla, T. R.; Tumber, A.; Schofield, C. J.; Brem, J., Targeting the Mycobacterium tuberculosis transpeptidase LdtMt2 with cysteine-reactive inhibitors including ebselen. *Chem. Commun.* **2019,** *55*, 10214-10217.
4. Schörgenhumer, J.; Waser, M., New strategies and applications using electrophilic cyanide-transfer reagents under transition metal-free conditions. *Org. Chem. Front.* **2016,** *3* (11), 1535-1540.
5. Charpentier, J.; Früh, N.; Togni, A., Electrophilic Trifluoromethylation by Use of Hypervalent Iodine Reagents. *Chem. Rev.* **2015,** *115* (2), 650-682.
6. Sameshima, T.; Miyahisa, I.; Yamasaki, S.; Gotou, M.; Kobayashi, T.; Sakamoto, J., High-Throughput Quantitative Intrinsic Thiol Reactivity Evaluation Using a Fluorescence-Based Competitive Endpoint Assay. *Adv. Sci. Drug Disc.* **2017,** *22*, 1168-1174.
